# A 32-year trend analysis of lower respiratory infections in children under 5: insights from the global burden of disease study 2021

**DOI:** 10.3389/fpubh.2025.1483179

**Published:** 2025-01-22

**Authors:** Yan Wang, Ruiyang Han, Xiao Ding, Junli Chen, Wenjia Feng, Chunping Wang, Runguo Gao, Anning Ma

**Affiliations:** ^1^School of Public Health, Shandong Second Medical University, Weifang, China; ^2^Institute of Public Health Crisis Management, Shandong Second Medical University, Weifang, China

**Keywords:** lower respiratory infections, incidence, mortality, disability-adjusted life years, global burden of disease

## Abstract

**Objectives:**

Lower respiratory infections are the most significant health threat to children under 5 years old, leading to the highest disease burden across all age groups. This study aims to provide an up-to-date assessment of the global burden of lower respiratory infections in children under 5 years of age.

**Methods:**

This study utilizes data and methodologies from the Global Burden of Disease Study 2021 to analyze changes in the burden of lower respiratory infections from 1990 to 2021, focusing on incidence, mortality, and disability-adjusted life years. A jointpoint model is employed to calculate trends and the average annual percentage change in the disease burden among children under 5 years old over the period 1990–2021. Additionally, frontier analysis is used to visually depict the potential for burden reduction in each country or region based on their level of development.

**Results:**

In 2021, the global burden of lower respiratory infections in children under 5 years old included 37,828,159 incidence cases, 501,909.50 deaths, and 44,779,174.70 disability-adjusted life years. From 1990 to 2021, the global burden of LRIs in this age group showed a marked decline. Incidence, mortality, and disability-adjusted life years decreased by 54.02, 37.57, and 39.49%, respectively. The average annual percent change for age-standardized incidence rate, age-standardized mortality rate, and age-standardized disability-adjusted life years rate were −3.35, −4.53%, and −4.52%, respectively. The disease burden was notably higher in children under 1 year old compared to those aged 2–4 years and the overall under-5 age group, with significant gender differences observed. Additionally, there was a strong negative correlation between the burden of lower respiratory infections in children under 5 and the socio-demographic index. Frontier analysis indicated that countries or regions with higher socio-demographic index values showed greater potential for reducing the burden.

**Conclusion:**

The global burden of lower respiratory infections in children under 5 years old has declined significantly from 1990 to 2021. However, given the substantial disease burden, particularly in low-SDI countries, it is crucial to address risk factors and implement more effective interventions to further reduce the impact of lower respiratory infections on this vulnerable population.

## Introduction

Lower respiratory infections (LRIs) are caused by various bacterial and viral pathogens, including *Streptococcus pneumoniae*, *Haemophilus influenzae* type B, *Staphylococcus aureus*, the influenza virus, and respiratory syncytial virus. Common illnesses resulting from these infections, including pneumonia, bronchitis, and bronchopneumonia, are predominantly caused by pneumococcal bacteria and represent a significant global cause of mortality (1–3). The Global Burden of Disease (GBD) study attributes more than 344 million cases and over 2.18 million fatalities worldwide to LRIs (4). Alarmingly, more than 75% of these deaths occur in low-and middle-income countries (4, 5). Children under 5 years of age contribute approximately 13.32% of the global death toll, accounting for around 12.55% of disability-adjusted life years (DALYs) (6). Therefore, addressing LRIs in children under five is crucial for reducing the overall global health burden associated with these infections (7).

Research utilizing data from the GBD database underscores that LRIs remain a substantial global health challenge (8). The primary pathogens associated with illness and mortality are *Streptococcus pneumoniae* (4, 9) and respiratory syncytial virus (10), while malnutrition among children under five persists as a major risk factor for death (11). While increased vaccination rates and reduced air pollution have substantially lowered child mortality rates (12), low-and middle-income countries (LMICs) still bear a disproportionate burden of the LRIs (10). LRIs pose a significant threat to young children, particularly in developing countries, where they account for 20–40% of pediatric hospital visits (13). The inappropriate use of antibiotics has unfortunately led to a rise in antibiotic resistance, further complicating treatment strategies (14). In LMICs, frequent misuse of antibiotics—averaging more than 24 prescriptions per child by the age of five—exacerbates the challenges of managing LRIs and significantly elevates resistance rates (15).

In response to the global challenge of LRIs, numerous international efforts have been spearheaded by organizations such as the World Health Organization (WHO) and the United Nations Children’s Fund (UNICEF). Among these initiatives, notable examples include the Global Action Plan for the Prevention and Control of Pneumonia and Diarrhea (GAPPD) (16), and the Integrated Management of Childhood Illness (IMCI) initiative (17). Beginning in 2020, the COVID-19 pandemic prompted the widespread implementation of non-pharmaceutical interventions, including lockdowns, the closure of schools and public spaces, and mandatory mask-wearing. These measures significantly reduced the incidence of respiratory infections during 2020 and 2021 (18–21).

This study aims to utilize data from GBD 2021 to comprehensively assess the global burden and trends of LRIs in children under 5 years of age, with the goal of raising international awareness of this critical issue. The study focuses on an in-depth exploration of the epidemiology of LRIs, including comprehensive trend analyses at global, regional, and national levels. Additionally, it also investigates the relationship between the disease burden of LRIs and the socio-demographic index (SDI) through a frontier analytical perspective.

## Materials and methods

### Study data

The GBD 2021 study provided a comprehensive assessment of health losses associated with 369 diseases, injuries, and impairments and 87 risk factors in 204 countries and territories using the most recent epidemiological data and advanced standardized methods, all disaggregated by age and sex. Our study utilized data on the burden of disease due to LRIs in children under 5 years of age from the GBD 2021 study, with specific burden of disease indicators including, e.g., incidence, mortality, and DALYs (22).

The SDI reflects the socio-economic status of a country and is calculated from the geometric mean of three indicators: the average educational attainment of the population over 15 years of age, the total fertility rate of the population under 25 years of age, and the distribution of per capita income (5). According to the SDI, the data were categorized into five regions (23), which helped this study explore health outcomes in different socioeconomic contexts. Meanwhile, the detailed data for 204 countries and territories were further categorized into 21 geographic regions (23, 24). LRIs diseases and related health problems according to the International Statistical Classification, Tenth Revision (ICD-10) were used to classify LRIs into specific groups: A48.1, A70, B96.0–B96.1, B97.21, B97.4–B97.6, J09–J18.2, J18.8–J1 8.9, J19.6–J22.9, J85.1, J91.0, P23–P23.9, U04–U04.9, and Z25.1 (25).

### Descriptive analysis

In order to get a comprehensive picture of the burden of LRIs in children under 5 years of age, descriptive analyses were conducted at global, regional and national scales. The number of global cases of LRIs, age-standardized rates (ASRs) were visualized for the period of 1990–2021, which were presented separately. Data on ASRs included age-standardized incidence rates (ASIR), age-standardized mortality rates (ASMR), and age-standardized disability-adjusted life years (ASDR) (26), while 95% uncertainty intervals (UI) were reported for all data. The UI is derived from estimates within 1,000 sub-zones for each parameter, with the 95% UI representing the range between the 25th and 975th ranked values among these samples (10). In addition, the number of cases of ASIR, ASMR, and ASDR were compared between 1990 and 2021 at global, regional and national levels and for both sexes in five SDI classes (high, medium-high, medium, medium-low and low) (27).

### Joinpoint regression analysis

Investigating the temporal trends of diseases is essential for guiding epidemiological strategies and refining prevention efforts. The objective of this study was to analyze LRIs trends from broad, and localized perspectives. Standardization ensures accurate comparisons between groups with different age distributions or for assessing changes within a specific group’s age structure over time.

We utilized joinpoint regression analysis to uncover local trends in the burden of LRIs. This approach, carried out using the Joinpoint Trend Analysis software (2022), segments the overall trend into multiple subperiods by identifying inflection points. For each subsegment, the magnitude of the epidemiological trend was assessed by calculating the annual percentage change (APC) and its corresponding 95% certainty intervals (CI) (28, 29). he Monte Carlo permutation method (10), applied with 4,499 randomly permuted datasets, was used to estimate the average annual percentage change (AAPC) and its 95% CI. To adjust for multiple comparisons, the overall significance level was corrected using the Bonferroni method. An increasing trend was indicated when both the APC/AAPC estimate and the lower bound of the 95% CI exceeded 0. Conversely, a decreasing trend was observed if both the APC/AAPC estimate and the upper bound of the 95% CI fell below 0. When neither of these conditions was met, the trend for that period was considered stable (30, 31).

### Frontier analysis

A quantitative method known as “frontier analysis” was utilized to determine the lowest achievable ASDR for LRIs, taking into account the SDI as a measure of socio-economic development for each country or region. This method evaluates the relationship between respiratory infection rates and socio-economic progress, as described in previous studies (32). The concept of “effective differences” represents the gap between a country’s or region’s observed ASDR and the frontier; larger gaps signify greater potential for reducing LRI-related DALYs. To define the frontier for ASDRs based on SDI, a network analysis employing the free disposal hull methodology was applied, incorporating data from 1990 to 2021. Nonlinear boundaries reflecting the association between SDI and ASDRs were established through this approach (33).

To address uncertainty, 1,000 iterations of self-sampling, random sampling, and replacement were performed across all years and countries or regions. For each SDI value, average DALYs were calculated from the resampled data. Subsequently, a LOESS regression with a local polynomial of degree 1 and a span of 0.2 was applied to generate a smooth boundary (34). Using 2021 SDI and ASDR data, the effective difference for each country or region was calculated as the absolute distance from the frontier. Countries or regions with DALYs rates below the frontier were assigned a distance of zero. This analysis provides insights into the association between DALYs rates and the frontier, age-standardized for the year 2021 (35).

Statistical analysis for this study was conducted using R software (version 4.3.3), with a *p*-value of less than 0.05 considered statistically significant.

## Results

### Incidence, mortality, and DALYs due to LRIs in children under 5 years old by global and SDI regions in 2021

Even though regions with lower SDIs continue to experience disproportionately high rates, overall, the study observed substantial reductions in LRIs burden. In 2021, the global burden of LRIs in children under 5 years old remained significant. An estimated 37.8 million new cases were reported (95% UI: 33.5 ~ 43.0 million), resulting in approximately 501,910 deaths (95% UI: 407,757 ~ 605,405) and 44.8 million DALYs lost (95% UI: 36.4 ~ 54.0 million). Between 1990 and 2021, substantial progress was made, with reductions of 54.02% in incidence, 37.57% in mortality, and 39.49% in DALYs for this vulnerable age group. The average annual percentage change (AAPC) in age-standardized rates showed consistent declines across incidence (−3.35, 95% CI: −3.59 to −3.10), mortality (−4.53, 95% CI: −4.65 to −4.40), and DALYs (−4.52, 95% CI: −4.65 to −4.40), all statistically significant (*p* < 0.001).

**Figure 1:**
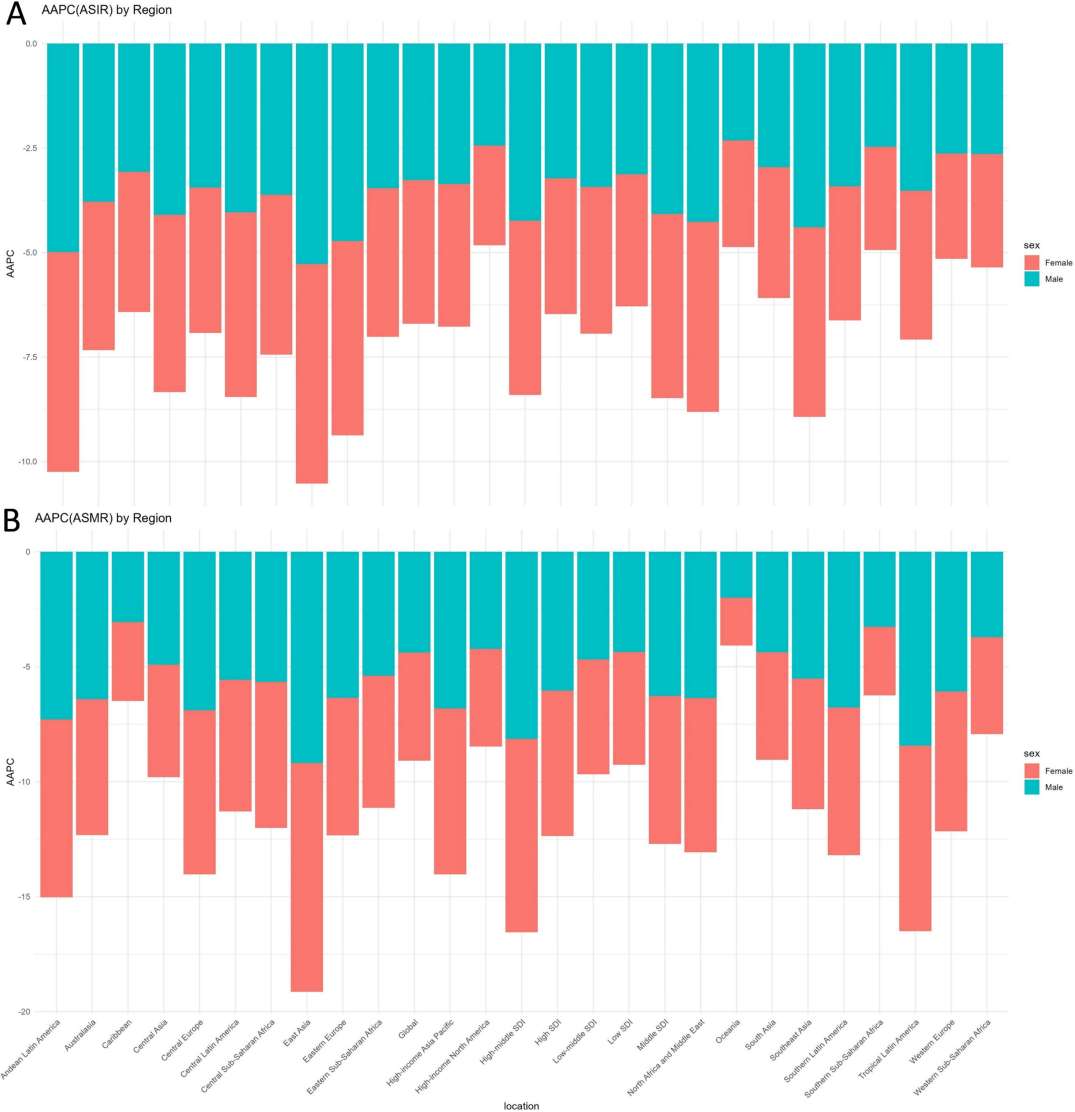
Global and regional changes trends in ASIR **(A)** and ASMR **(B)** of LRIs in children under 5: AAPC by sex, 1990–2019. ASIR, Age-Standardized Incidence Rate; ASMR, Age-Standardized Mortality Rate; AAPC, Average Annual Percentage Change; LRIs, Lower Respiratory Infections.

In 2021, the burden of LRIs in children under 5 years of age was concentrated in low-middle and low SDI regions, which accounted for more than 74% of global cases. These regions have the highest case numbers of incidence, mortality, and DALYs, as well as ASIR, ASMR, and ASDR. Between 1990 and 2021, significant progress has been made in controlling the global burden of disease in LRIs in children under 5 years of age, with significant declines in LRI-related indicators in all SDI regions. However, the rate and magnitude of improvement varied across regions. From 1990 to 2021, all SDI regions experienced significant reductions in ASIR, ASMR, and ASDR. The high-middle SDI region saw the largest decrease in ASIR at 68.71%, while the high SDI region had the largest declines in ASMR and ASDR, at 61.24 and 66.87%, respectively. Furthermore, the middle SDI region exhibited the fastest decline in ASIR, with an AAPC of −4.22% (95% CI: −4.37 to −4.06). The high-middle SDI region showed the most rapid decreases in ASMR and ASDR, with AAPCs of −8.25% (95% CI: −8.69 to −7.81) and −8.23% (95% CI: −8.67 to −7.79), respectively (all *p* < 0.001) (see Table 1; Figure 1).

**Table 1:** Incidence, mortality, DALYs cases, ASRs, and AAPC values of LRIs under 5 years old in the global, SDI, and GBD regions in 2021.

Characteristic	Incidence cases (95%UI)	ASIR per 100,000 (95% UI)	Percentage change (95%CI)	ASIR AAPC1990-2021 (%)	Mortality cases (95%UI)	ASMR per 100,000 (95% UI)	Percentage change (95%CI)	ASMR AAPC1990-2021 (%)	DALYs cases (95%UI)	ASDR per 100,000 (95% UI)	Percentage change (95%CI)	ASDR AAPC1990-2021 (%)
Overall	37,828,159 (33,469,075.80 ~ 43,025,156.91)	5,747.46 (5,085.16 ~ 6,537.07)	−54.02 (−56.6 ~ −51.77)	−3.35 (−3.59 ~ −3.1)	501,909.5 (407,756.99 ~ 605,404.87)	76.26 (61.95 ~ 91.98)	−37.57 (−43.77 ~ −30.73)	−4.53 (−4.65 ~ −4.4)	44,779,174.7 (36,403,622.57 ~ 53,964,530.53)	6,803.57 (5,531.02 ~ 8,199.16)	−39.49 (−45.45 ~ −32.91)	−4.52 (−4.65 ~ −4.4)
SDI regions												
Low SDI	14,041,192.62 (12,489,335.76 ~ 15,871,801.5)	8,480.25 (7,543 ~ 9,585.85)	−45.32 (−48.34 ~ −42.43)	−3.13 (−3.33 ~ −2.94)	253,931.28 (195,867.89 ~ 316,840.33)	153.36 (118.3 ~ 191.36)	−34.76 (−43.13 ~ −24.36)	−4.62 (−4.71 ~ −4.53)	22,599,130.82 (17,451,962.5 ~ 28,163,166.91)	13,648.86 (10,540.2 ~ 17,009.28)	−36.09 (−44.19 ~ −26.2)	−4.61 (−4.7 ~ −4.53)
Low-middle SDI	14,068,565.58 (12,385,268.8 ~ 16,009,193.9)	7,343.52 (6,464.87 ~ 8,356.49)	−50.55 (−53.98 ~ −47.24)	−3.45 (−3.7 ~ −3.2)	180,097.54 (148,585.78 ~ 212,833.65)	94.01 (77.56 ~ 111.1)	−29.89 (−37.59 ~ −20.08)	−4.74 (−4.97 ~ −4.51)	16,103,471.78 (13,288,822.85 ~ 19,026,544.06)	8,405.71 (6,936.51 ~ 9,931.49)	−32.21 (−39.9 ~ −22.83)	−4.73 (−4.97 ~ −4.5)
Middle SDI	6,852,145.15 (5,991,915.65 ~ 7,893,180.74)	3,879.66 (3,392.6 ~ 4,469.09)	−65.78 (−67.99 ~ −63.42)	−4.22 (−4.37 ~ −4.06)	60,449.81 (51,107.66 ~ 70,691.94)	34.23 (28.94 ~ 40.03)	−47.93 (−52.5 ~ −43.39)	−6.34 (−6.58 ~ −6.1)	5,408,861.23 (4,573,006 ~ 6,324,269.6)	3,062.48 (2,589.22 ~ 3,580.78)	−51.33 (−55.71 ~ −46.94)	−6.34 (−6.58 ~ −6.09)
High-middle SDI	2,243,455.18 (1,881,882.48 ~ 2,616,469.41)	3,202.88 (2,686.68 ~ 3,735.42)	−68.71 (−71.84 ~ −65.43)	−4.21 (−4.25 ~ −4.17)	6,005.37 (5,108.39 ~ 7,043.91)	8.57 (7.29 ~ 10.06)	−60.35 (−67.44 ~ −54.63)	−8.25 (−8.69 ~ −7.81)	539,556.46 (459,580.58 ~ 632,536.1)	770.3 (656.12 ~ 903.04)	−65.78 (−71.95 ~ −60.44)	−8.23 (−8.67 ~ −7.79)
High SDI	599,829.88 (512,810.76 ~ 701,571.39)	1,113.97 (952.36 ~ 1,302.92)	−62.44 (−64.6 ~ −60.39)	−3.23 (−3.34 ~ −3.12)	997.78 (892.94 ~ 1,080.26)	1.85 (1.66 ~ 2.01)	−61.24 (−66.65 ~ −57.39)	−6.14 (−6.65 ~ −5.62)	89,928.09 (80,790.15 ~ 97,272.24)	167.01 (150.04 ~ 180.65)	−66.87 (−71.37 ~ −63.5)	−6.12 (−6.57 ~ −5.67)
GBD regions												
Andean Latin America	244,929.76 (201,588.39 ~ 290,370.74)	3,978.84 (3,274.77 ~ 4,717.02)	−67.94 (−72.34 ~ −63.26)	−5.1 (−5.6 ~ −4.6)	2,194.28 (1,660.69 ~ 2,806.09)	35.65 (26.98 ~ 45.58)	−53.44 (−62.14 ~ −43.75)	−7.52 (−8.17 ~ −6.86)	196,075.73 (148,554.98 ~ 250,440.97)	3,185.22 (2,413.25 ~ 4,068.37)	−56.57 (−64.93 ~ −47.7)	−7.51 (−8.15 ~ −6.86)
Australasia	8,809.33 (7,392.29 ~ 10,561.28)	485.08 (407.05 ~ 581.55)	−66.83 (−69.57 ~ −63.9)	−3.79 (−4.21 ~ −3.36)	19.39 (16.1 ~ 22.87)	1.07 (0.89 ~ 1.26)	−56 (−62.28 ~ −49.66)	−6.2 (−8.31 ~ −4.04)	1,747.77 (1,454.74 ~ 2,060.09)	96.24 (80.1 ~ 113.44)	−64.03 (−69.5 ~ −58.5)	−6.18 (−8.28 ~ −4.04)
Caribbean	154,022.46 (134,855.55 ~ 174,276.35)	3,981.78 (3,486.28 ~ 4,505.38)	−58.92 (−61.57 ~ −56.24)	−3.2 (−3.29 ~ −3.11)	2,914.42 (2,041.42 ~ 3,855.32)	75.34 (52.77 ~ 99.67)	−35.35 (−50.68 ~ −16.42)	−3.23 (−3.53 ~ −2.92)	260,572.6 (182,508.57 ~ 344,860.12)	6,736.31 (4,718.2 ~ 8,915.31)	−36.74 (−51.62 ~ −18.52)	−3.22 (−3.53 ~ −2.92)
Central Asia	329,289.24 (298,311.61 ~ 368,137.9)	3,293.87 (2,984 ~ 3,682.47)	−66.02 (−68.47 ~ −63.02)	−4.31 (−4.51 ~ −4.11)	9,894.41 (8,195.06 ~ 12,018.37)	98.97 (81.97 ~ 120.22)	−45.59 (−51.54 ~ −40.3)	−4.91 (−5.19 ~ −4.63)	884,372.79 (732,758.11 ~ 1,073,959.58)	8,846.36 (7,329.76 ~ 10,742.79)	−48.28 (−54.08 ~ −43.16)	−4.91 (−5.19 ~ −4.62)
Central Europe	116,216.69 (102,088.39 ~ 132,680.49)	2,080.62 (1,827.68 ~ 2,375.37)	−65.6 (−68.34 ~ −62.9)	−3.45 (−3.75 ~ −3.16)	438.94 (371.72 ~ 508.27)	7.86 (6.65 ~ 9.1)	−56.05 (−60.87 ~ −51.87)	−6.99 (−7.95 ~ −6.01)	39,398.66 (33,439.18 ~ 45,568.74)	705.35 (598.66 ~ 815.81)	−63.74 (−68.22 ~ −59.56)	−6.98 (−7.94 ~ −6.01)
Central Latin America	486,979.29 (421,551.72 ~ 562,282.67)	2,423.94 (2,098.27 ~ 2,798.76)	−68.18 (−70.87 ~ −65.71)	−4.19 (−4.27 ~ −4.12)	5,154.89 (3,931.92 ~ 6,696.57)	25.66 (19.57 ~ 33.33)	−46.29 (−50.21 ~ −42.81)	−5.64 (−6.22 ~ −5.04)	461,181.9 (351,965.54 ~ 598,739.55)	2,295.53 (1,751.91 ~ 2,980.23)	−49.33 (−53.92 ~ −45.5)	−5.63 (−6.22 ~ −5.04)
Central Sub-Saharan Africa	1,184,774.35 (1,020,922.91 ~ 1,374,232.90)	5,623.97 (4,846.19 ~ 6,523.31)	−55.62 (−60 ~ −50.14)	−3.71 (−3.77 ~ −3.65)	17,420.39 (12,590.6 ~ 23,557.75)	82.69 (59.77 ~ 111.83)	−50.11 (−59.24 ~ −38.31)	−5.96 (−6.04 ~ −5.89)	1,553,341.09 (1,124,046.8 ~ 2,097,465.67)	7,373.51 (5,335.71 ~ 9,956.4)	−51.19 (−60.12 ~ −39.97)	−5.96 (−6.03 ~ −5.88)
East Asia	2,488,692.62 (2,075,859.19 ~ 2,940,477.91)	3,108 (2,592.44 ~ 3,672.22)	−74.89 (−78.24 ~ −71.65)	−5.26 (−5.35 ~ −5.17)	10,504.62 (8,258.93 ~ 13,010.75)	13.12 (10.31 ~ 16.25)	−53.78 (−64.85 ~ −45.03)	−9.44 (−9.91 ~ −8.97)	942,677.7 (741,333.49 ~ 1,166,294.06)	1,177.26 (925.81 ~ 1,456.53)	−60.03 (−69.7 ~ −52.21)	−9.43 (−9.89 ~ −8.95)
Eastern Europe	161,871.8 (134,048.97 ~ 190,744.88)	1,599.69 (1,324.74 ~ 1,885.03)	−75.04 (−77.94 ~ −71.63)	−4.69 (−4.84 ~ −4.55)	707.13 (640.47 ~ 773.41)	6.99 (6.33 ~ 7.64)	−54.58 (−58.14 ~ −51.95)	−6.17 (−6.94 ~ −5.39)	63,460.95 (57,517.78 ~ 69,356.02)	627.15 (568.42 ~ 685.41)	−60.79 (−64.25 ~ −57.86)	−6.17 (−6.94 ~ −5.39)
Eastern Sub-Saharan Africa	4,805,902.16 (4,239,811.28 ~ 5,475,722.44)	7,533.19 (6,645.85 ~ 8,583.12)	−46.93 (−50.56 ~ −43.24)	−3.5 (−3.83 ~ −3.17)	64,447.17 (49,146.21 ~ 80,239.54)	101.02 (77.04 ~ 125.77)	−42.07 (−50.32 ~ −31.34)	−5.57 (−5.86 ~ −5.28)	5,742,577.21 (4,385,989.28 ~ 7,144,385.48)	9,001.42 (6,874.98 ~ 11,198.73)	−43.42 (−51.55 ~ −33.29)	−5.56 (−5.85 ~ −5.28)
High-income Asia Pacific	103,039.15 (86,211.82 ~ 122,375.67)	1,596.97 (1,336.17 ~ 1,896.66)	−65.54 (−68.74 ~ −62.3)	−3.39 (−3.44 ~ −3.34)	67.45 (60.67 ~ 73.82)	1.05 (0.94 ~ 1.14)	−59.6 (−63.5 ~ −55.6)	−7 (−7.8 ~ −6.18)	6,156.09 (5,559.46 ~ 6,706.37)	95.41 (86.16 ~ 103.94)	−71.26 (−74.94 ~ −67.83)	−6.95 (−7.74 ~ −6.15)
High-income North America	189,499.05 (164,582.91 ~ 217,309.14)	924.45 (802.9 ~ 1,060.12)	−50.58 (−54.2 ~ −46.31)	−2.42 (−2.68 ~ −2.16)	334.14 (291.55 ~ 381.09)	1.63 (1.42 ~ 1.86)	−51.39 (−56.89 ~ −46.5)	−4.28 (−5.79 ~ −2.74)	30,080.14 (26,260.12 ~ 34,262.58)	146.74 (128.11 ~ 167.15)	−55.75 (−61.25 ~ −50.95)	−4.27 (−5.77 ~ −2.75)
North Africa and Middle East	1,665,999.23 (1,427,458.96 ~ 1,957,640.69)	2,725.02 (2,334.85 ~ 3,202.05)	−65.21 (−68.22 ~ −61.8)	−4.4 (−4.55 ~ −4.26)	20,252.43 (16,721.27 ~ 24,498.27)	33.13 (27.35 ~ 40.07)	−46.95 (−56.73 ~ −37.97)	−6.53 (−6.68 ~ −6.39)	1,810,545.41 (1,495,519.9 ~ 2,189,375.71)	2,961.45 (2,446.17 ~ 3,581.09)	−50.19 (−59.39 ~ −41.85)	−6.53 (−6.68 ~ −6.38)
Oceania	156,279.47 (136,471.39 ~ 178,495.67)	8,078.75 (7,054.79 ~ 9,227.2)	−46.77 (−52.43 ~ −38.51)	−2.43 (−2.53 ~ −2.33)	4,644.69 (3,611.13 ~ 5,961.47)	240.1 (186.67 ~ 308.17)	−22.25 (−35.42 ~ −6.74)	−2.04 (−2.39 ~ −1.68)	415,308.48 (322,939.91 ~ 533,148.6)	21,469.06 (16,694.14 ~ 27,560.72)	−23.12 (−36.01 ~ −8.01)	−2.04 (−2.39 ~ −1.68)
South Asia	15,268,815.47 (13,377,569.26 ~ 17,657,690.79)	9,627.63 (8,435.12 ~ 11,133.91)	−38.94 (−44.09 ~ −33.68)	−3.01 (−3.11 ~ −2.9)	154,298.94 (125,520.06 ~ 189,368.07)	97.29 (79.15 ~ 119.4)	−21.39 (−32.99 ~ −6.44)	−4.6 (−4.93 ~ −4.27)	13,817,654.63 (11,246,228.25 ~ 16,948,155.41)	8,712.61 (7,091.22 ~ 10,686.52)	−24.7 (−36.12 ~ −10.92)	−4.54 (−4.87 ~ −4.21)
Southeast Asia	2,235,455.34 (1,959,402.07 ~ 2,549,064.94)	3,971.7 (3,481.24 ~ 4,528.89)	−70.12 (−71.72 ~ −68.24)	−4.45 (−4.64 ~ −4.26)	26,524.66 (21,513.98 ~ 32,717.15)	47.13 (38.22 ~ 58.13)	−41.23 (−49.72 ~ −32.22)	−5.59 (−5.71 ~ −5.47)	2,370,653.38 (1,922,730.71 ~ 2,922,979.46)	4,211.91 (3,416.09 ~ 5,193.22)	−44 (−52.28 ~ −35.4)	−5.58 (−5.7 ~ −5.46)
Southern Latin America	84,900.72 (70,598.05 ~ 102,300.64)	1,984.37 (1,650.08 ~ 2,391.06)	−57.1 (−61.65 ~ −52.12)	−3.33 (−3.84 ~ −2.82)	252.34 (205.79 ~ 306.66)	5.9 (4.81 ~ 7.17)	−54.2 (−59.15 ~ −49.71)	−6.61 (−10.14 ~ −2.93)	22,641.09 (18,475.3 ~ 27,520.4)	529.19 (431.82 ~ 643.23)	−59.14 (−63.21 ~ −55.06)	−6.61 (−10.14 ~ −2.93)
Southern Sub-Saharan Africa	550,672.97 (485,069.56 ~ 633,250.15)	6,858.45 (6,041.38 ~ 7,886.93)	−36.38 (−40.92 ~ −31.14)	−2.53 (−2.85 ~ −2.2)	8,279.06 (6,704.42 ~ 9,964.55)	103.11 (83.5 ~ 124.11)	−28.37 (−38.36 ~ −18.49)	−3.11 (−3.52 ~ −2.7)	739,861.2 (599,622.08 ~ 889,845.94)	9,214.73 (7,468.1 ~ 11,082.74)	−29.53 (−39.39 ~ −19.93)	−3.13 (−3.53 ~ −2.74)
Tropical Latin America	799,029.38 (678,742.04 ~ 941,244.21)	4,643.47 (3,944.43 ~ 5,469.93)	−61.79 (−65.55 ~ −57.31)	−3.53 (−4.04 ~ −3.02)	1,918.34 (1,441.6 ~ 2,429.06)	11.15 (8.38 ~ 14.12)	−69.54 (−73.95 ~ −64.35)	−8.24 (−8.69 ~ −7.79)	172,164.94 (129,592.67 ~ 217,879.11)	1,000.52 (753.11 ~ 1,266.18)	−72.34 (−76.84 ~ −67.54)	−8.23 (−8.68 ~ −7.78)
Western Europe	123,687.6 (102,887.58 ~ 149,171.23)	582.63 (484.65 ~ 702.67)	−55.57 (−58.51 ~ −52.6)	−2.57 (−2.69 ~ −2.44)	181.95 (162.17 ~ 200.25)	0.86 (0.76 ~ 0.94)	−60.4 (−63.03 ~ −57.82)	−6.12 (−7.56 ~ −4.65)	16,420.25 (14,635.2 ~ 18,029.15)	77.35 (68.94 ~ 84.93)	−67.04 (−69.64 ~ −64.4)	−6.09 (−7.54 ~ −4.63)
Western Sub-Saharan Africa	6,669,292.92 (5,933,232.5 ~ 7,462,945.63)	8,340.98 (7,420.43 ~ 9,333.57)	−35.74 (−39.76 ~ −31.49)	−2.67 (−2.87 ~ −2.48)	171,459.88 (123,879.2 ~ 223,431.66)	214.44 (154.93 ~ 279.44)	−28.72 (−41.11 ~ −15.78)	−3.95 (−4.16 ~ −3.73)	15,232,282.64 (11,037,624.11 ~ 19,824,814.1)	19,050.33 (13,804.26 ~ 24,794)	−29.82 (−41.96 ~ −17.32)	−3.94 (−4.16 ~ −3.73)

DALYs, Disability-Adjusted Life Years; ASRs, Age-Standardized Rates; AAPC, Average Annual Percentage Change; LRIs, Lower Respiratory Infections; SDI, Socio-Economic Index; GBD, Global Burden of Disease.

### Incidence, mortality, and DALYs due to LRIs in children under 5 years old by GBD regions in 2021

In 2021, the burden of LRIs among children under 5 years old was notably concentrated in South Asia and Western Sub-Saharan Africa. South Asia accounted for over 40% of global LRIs cases, while Western Sub-Saharan Africa reported the highest mortality and DALYs. Substantial progress has been achieved since 1990, with many regions experiencing significant reductions in LRI-related indicators, particularly East Asia, which showed the fastest rates of decline across all metrics. South Asia had the highest incidence of LRIs among children under five in 2021, with 15,268,815 cases, representing over 40% of the global total. The Western Sub-Saharan Africa region recorded the highest mortality and DALYs cases, with 171,460 deaths and 15,232,283 DALYs, accounting for over 34% of the global burden. Regionally, South Asia exhibited the highest ASIR at 9,627.63, while Oceania reported the highest ASMR and ASDR at 240.10 and 1,469.06, respectively.

Between 1990 and 2021, Eastern Europe, East Asia, and Southeast Asia experienced the most significant reductions in ASIR, with declines exceeding 70%. The largest decreases in ASMR and ASDR occurred in Tropical Latin America, Western Europe, and the High-Income Asia Pacific region. East Asia demonstrated the fastest reductions across all metrics, with AAPCs of −5.26% for ASIR, −9.44% for ASMR, and −9.43% for ASDR. All changes were statistically significant (*p* < 0.001) (see Table 1; Figure 1).

**Figure 2:**
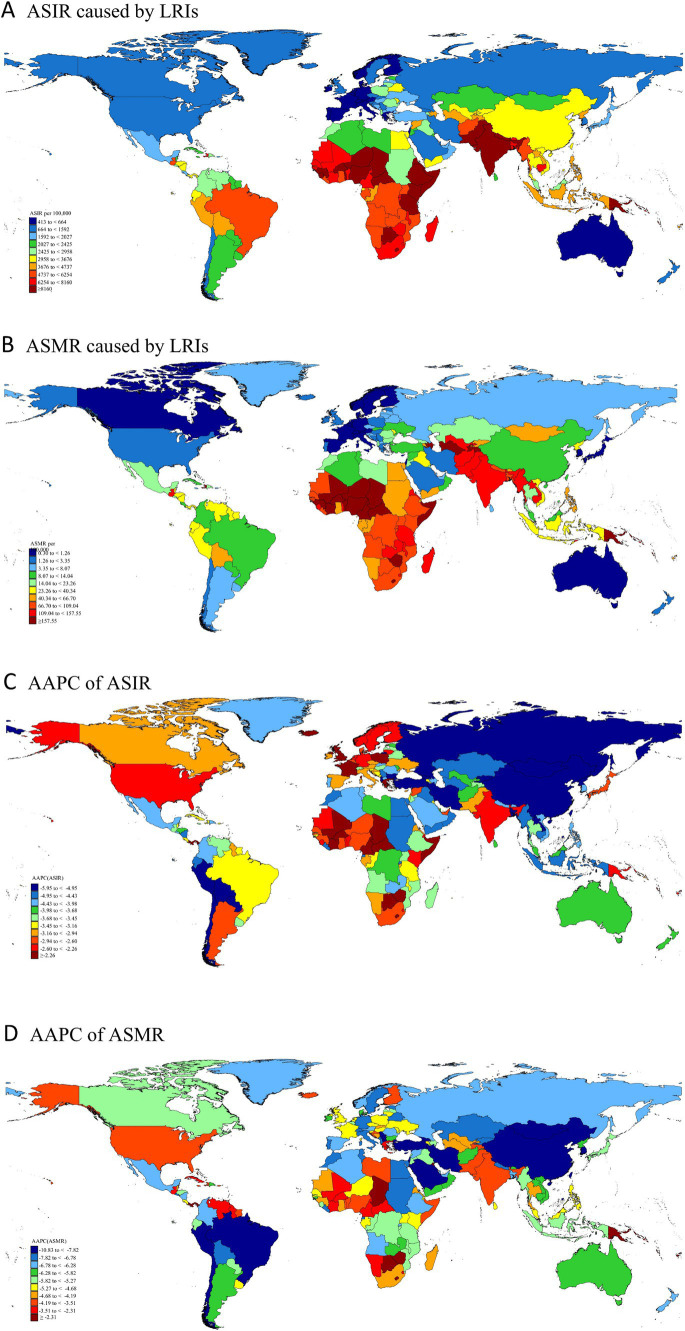
Global distribution and temporal changes in LRIs: ASIR **(A)**, ASDR **(B)**, and AAPC **(C,D)** in children under 5, 1990–2021. LRIs, Lower Respiratory Infections; ASIR, Age-Standardized Incidence Rate; ASMR, Age-Standardized Mortality Rate; AAPC, Average Annual Percentage Change.

The burden of disease for LRIs in children under 5 years of age has progressed significantly across countries globally since 1990, but previous progress across countries has been markedly inconsistent. At the national level, India accounted for over 20% of the global burden of LRIs among children under five, leading in incidence, mortality, and DALYs, with 10,384,348 cases, 1,026 deaths, and 9,396,066 DALYs, representing more than 20% of the global total in 2021. Pakistan recorded the highest ASIR, while Chad exhibited the highest ASMR and ASDR. Between 1990 and 2021, the largest relative declines in ASIR, ASDR, and ASMR occurred in Bulgaria, Chile, and the Republic of Korea, with reductions exceeding 80%. Turkey exhibited the fastest decrease in ASIR, with an AAPC of −5.94%. The Islamic Republic of Iran demonstrated the most rapid reductions in ASMR and ASDR, with AAPCs of −10.82% and −10.81%, respectively (see Supplementary Table S1; Figure 2).

### Age group trends in incidence, mortality, and DALYs due to LRIs in children under 5 years old (1990–2021)

From 1990 to 2021, the global burden of LRIs in children under 1 year of age and 2–4 years of age has declined significantly, with children in the under 1 year age group consistently experiencing the highest burden of disease in terms of the rates of incidence, mortality, and DALYs. These trends are reflected in all five SDI regions, with specific patterns varying over time. Globally, between 1990 and 2021, incidence, mortality, and DALYs due to LRIs declined by 48 and 55.62%, 40.48 and 31.07%, and 40.9 and 39.84%, respectively, for children under 1 and 2–4 years of age. In 2021, the burden of LRIs for the under-1 age group remained the highest, with a incidence rate of 10096.63 (95% UI: 9088.13–11303.85), a mortality rate of 282.67 (95% UI: 237.82–331.97), and DALYs of 25,385.43 (95% UI: 21,363.14–29,806.57).

**Figure 3:**
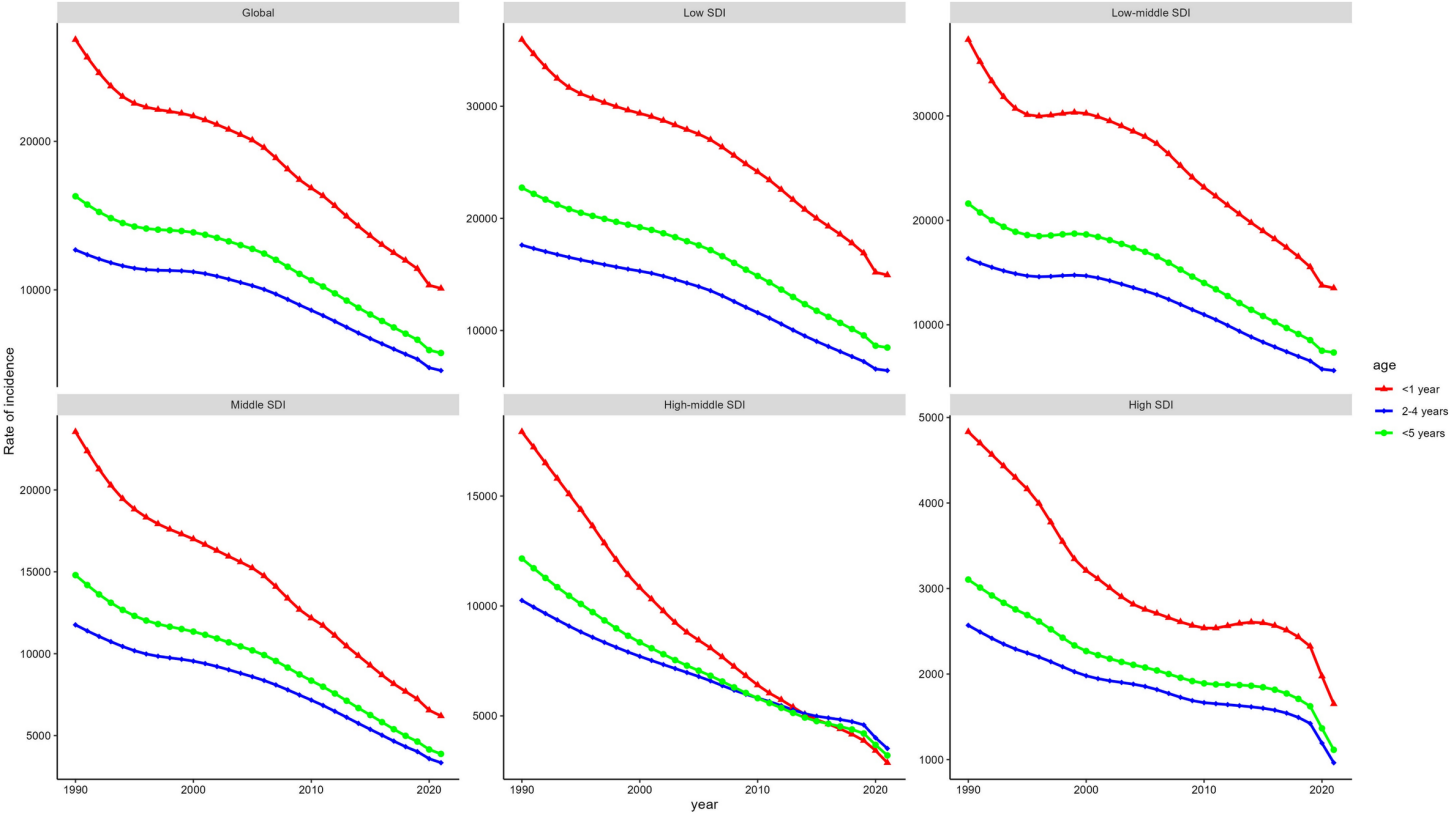
Global and regional incidence of LRIs in 3 age groups in 2021: insights from global and five SDI regions. LRIs, Lower Respiratory Infections; SDI, Socio-Economic Index.

Across the five SDI regions, trends in the burden of LRIs for children under 1, 2–4, and under 5 years of age mirrored global patterns. However, the middle and high SDI regions showed a unique variation: prior to 2014, the highest prevalence was among children under 1 year of age, but beginning in 2014, the prevalence among children 2–4 years of age exceeded the combined prevalence among children under 1 year of age and children under 5 years of age (see Figure 3; Supplementary Figures S1, S2).

**Figure 4:**
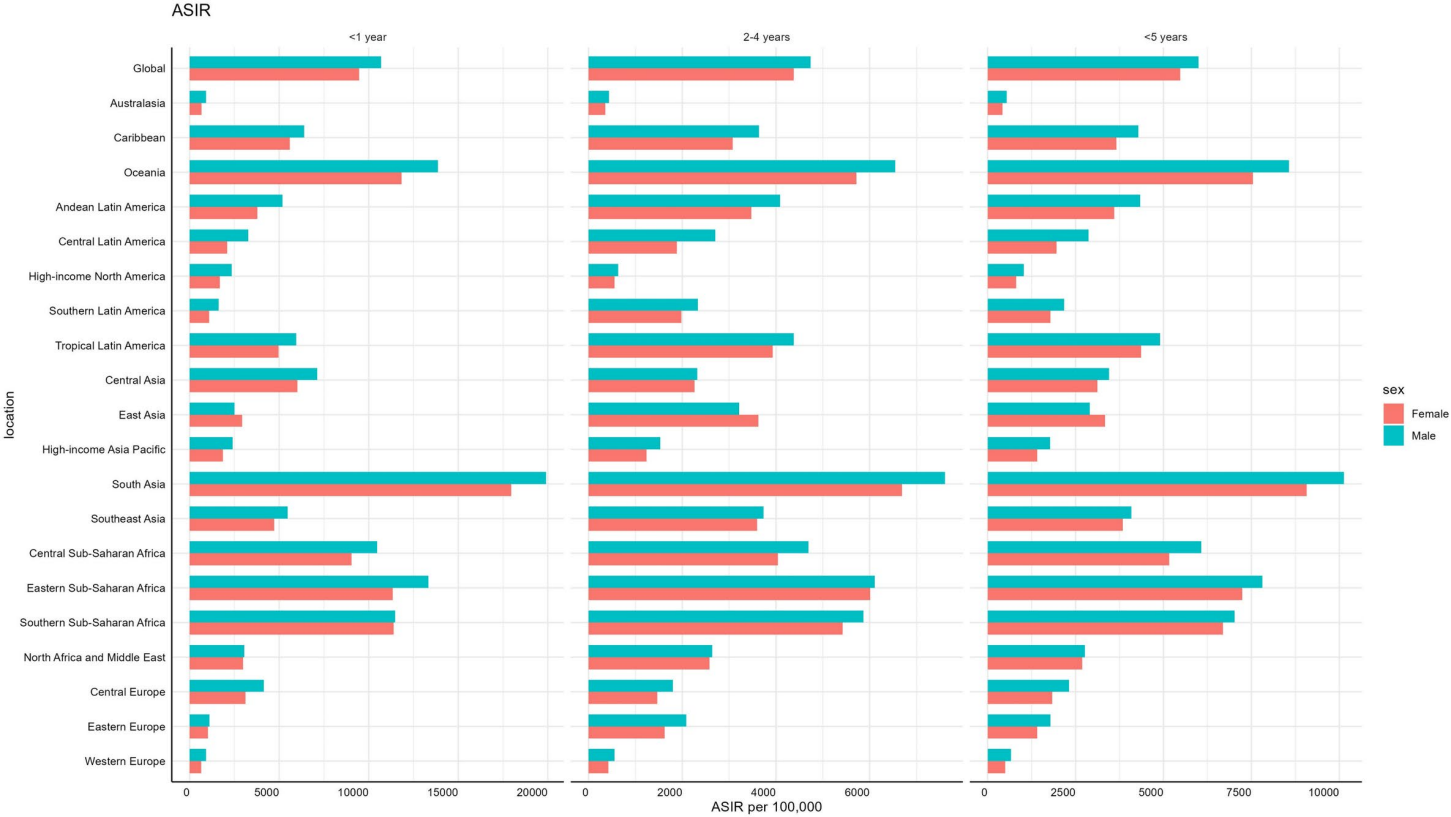
Age-specific and gender-based incidence rates of LRIs globally and regionally in children under 5 in 2021. LRIs, Lower Respiratory Infections.

In 21 GBD regions, gender differences in LRIs in children under 1 year, 2–4 years, and under 5 years of age were striking. In all 3 age groups, males generally had higher incidence rate than females, with notable exceptions in South Asia and sub-Saharan Africa, where females had higher mortality and DALYs rates. For children under 1 year old, the global incidence rate of LRIs was higher in males at 10,688.80 compared to females at 9,463.63 per 100,000. Similar patterns were observed in the 2–4 years age group, with males recording an incidence rate of 4,737.85 compared to females at 4,380.43 per 100,000. Among children under 5 years old, the incidence rate for males was 6,001.45, consistently higher than that of females. Gender disparities extended to mortality and DALYs rates for children under 1 year old, where males generally had higher rates globally. However, in South Asia and Southern Sub-Saharan Africa, females experienced higher mortality and DALYs rates than males (see Figure 4; see Supplementary Figures S3, S4).

### Relationship of ASRs with SDI

The burden of LRIs in children under 5 years of age was significantly and linearly related to the SDI both globally and in the 21 GBD regions. The ASIR, ASMR, and ASDR of LRIs in children under 5 years of age tended to increase significantly with decreasing SDI. South Asia and Sub-Saharan Africa had the highest burden, while Australasia and Western Europe had the lowest. This association highlights the disparity between the disease burden of LRIs and socioeconomic development in children under 5 years of age.

**Figure 5:**
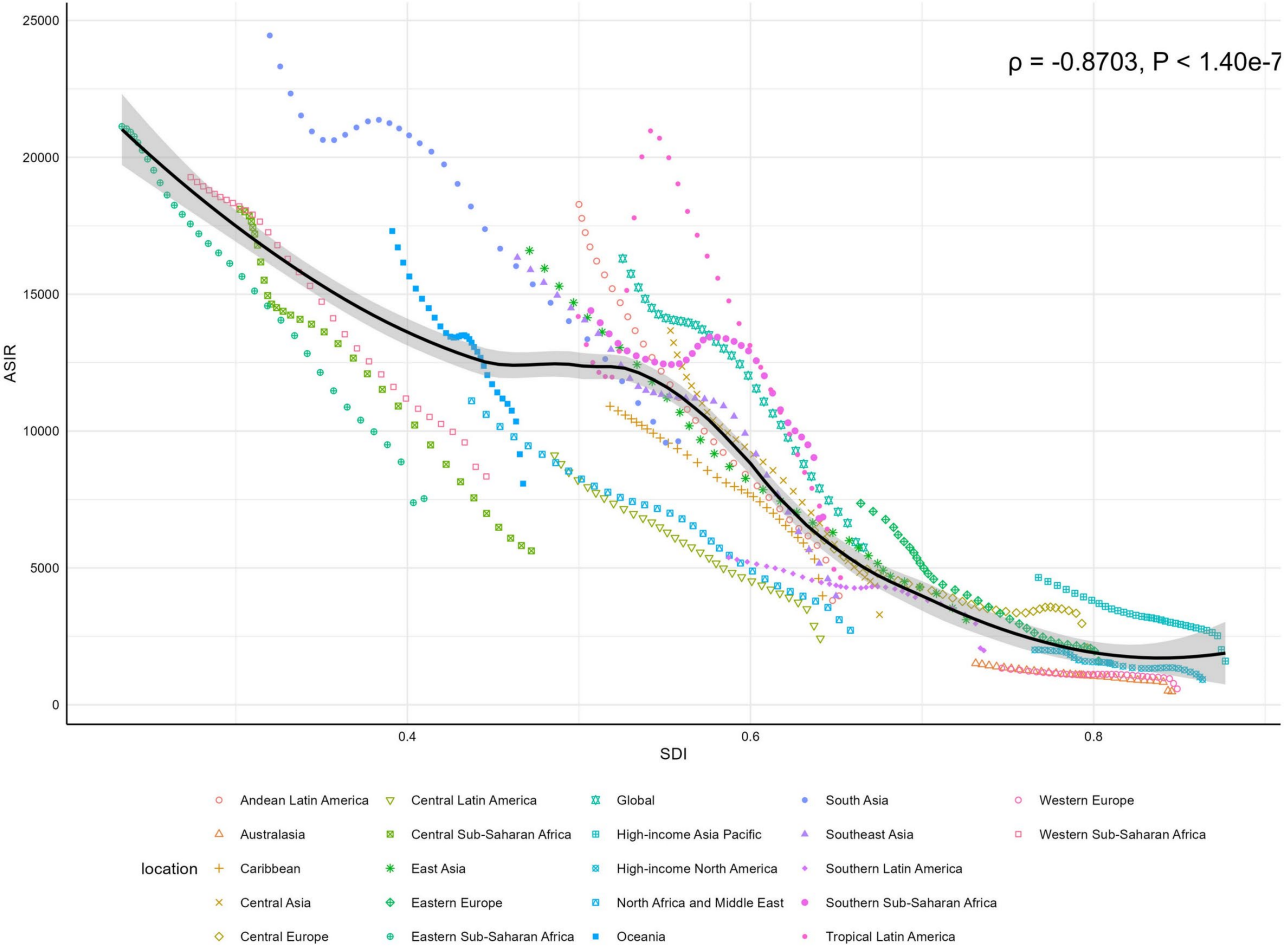
Global and regional correlations between ASIR of LRIs and SDI in children under 5, 1990–2021. ASIR, Age-Standardized Incidence Rate; LRIs, Lower Respiratory Infections; SDI, Socio-Economic Index.

Globally, a significant negative correlation was observed between SDI and ASIR (*ρ* = −0.8703, *p* < 0.001). South Asia recorded the highest ASIR at 24,450.88 per 100,000, with an SDI of 0.320, while the lowest ASIR was in Australasia at 485.08 per 100,000, where the SDI was 0.846. In 2021, six regions exceeded the global average ASIR, including South Asia, Western Sub-Saharan Africa, Oceania, Eastern Sub-Saharan Africa, and Southern Sub-Saharan Africa (see Figure 5). Similarly, linear associations were evident between SDI and both ASMR and ASDR globally and across regions (see Supplementary Figures S5, S6). Western Sub-Saharan Africa had the highest ASMR (737.36 per 100,000) and ASDR (65,396.63 per 100,000) with an SDI of 0.274. In contrast, the lowest ASMR (0.86 per 100,000) and ASDR (77.35 per 100,000) were observed in Western Europe, where the SDI was 0.849. Seven regions exceeded the global averages for ASMR and ASDR in 2021, including Central Sub-Saharan Africa, South Asia, Central Asia, Eastern Sub-Saharan Africa, Southern Sub-Saharan Africa, Western Sub-Saharan Africa, and Oceania (see Supplementary Figures S7, S8).

**Figure 6:**
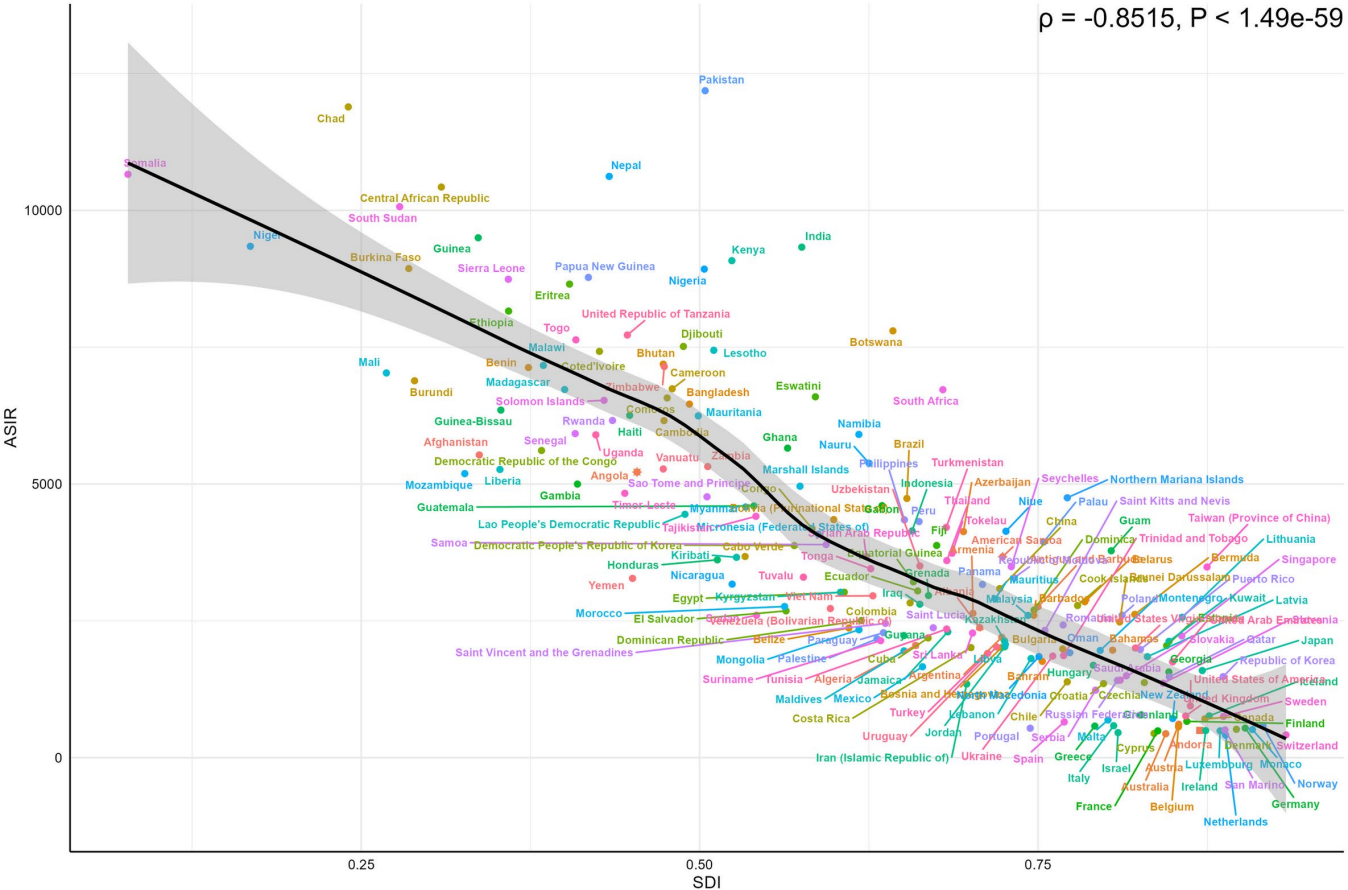
Country-level correlations between ASIR of LRIs and SDI in children under 5 in 2021. ASIR, Age-Standardized Incidence Rate; LRIs, Lower Respiratory Infections; SDI, Socio-Demographic Index.

At the national level, a clear relationship was observed between the SDI and the burden of LRIs in 2021. Higher SDI values were strongly associated with lower ASIR, ASMR, and ASDR. This negative correlation highlights significant disparities across countries, with lower-SDI regions facing a disproportionately higher burden of LRIs. A strong negative correlation was identified between ASIR and SDI values (*ρ* = −0.8515, *p* < 0.001), as illustrated in Figure 6. Among the 204 countries and territories analyzed, Pakistan, with an SDI of 0.504, recorded the highest ASIR at 12,185.93 per 100,000, while the Netherlands, with an SDI of 0.888, reported the lowest ASIR at 413.01 per 100,000. Similarly, ASMR and ASDR were significantly negatively correlated with SDI. Chad, with an SDI of 0.240, exhibited the highest ASMR at 358.31 per 100,000 and the highest ASDR at 31,793.76 per 100,000. Conversely, Andorra, with an SDI of 0.869, recorded the lowest ASMR at 0.338 per 100,000 and the lowest ASDR at 30.66 per 100,000. These relationships are further detailed in Supplementary Figures S7, S8.

### Frontier analysis

**Figure 7:**
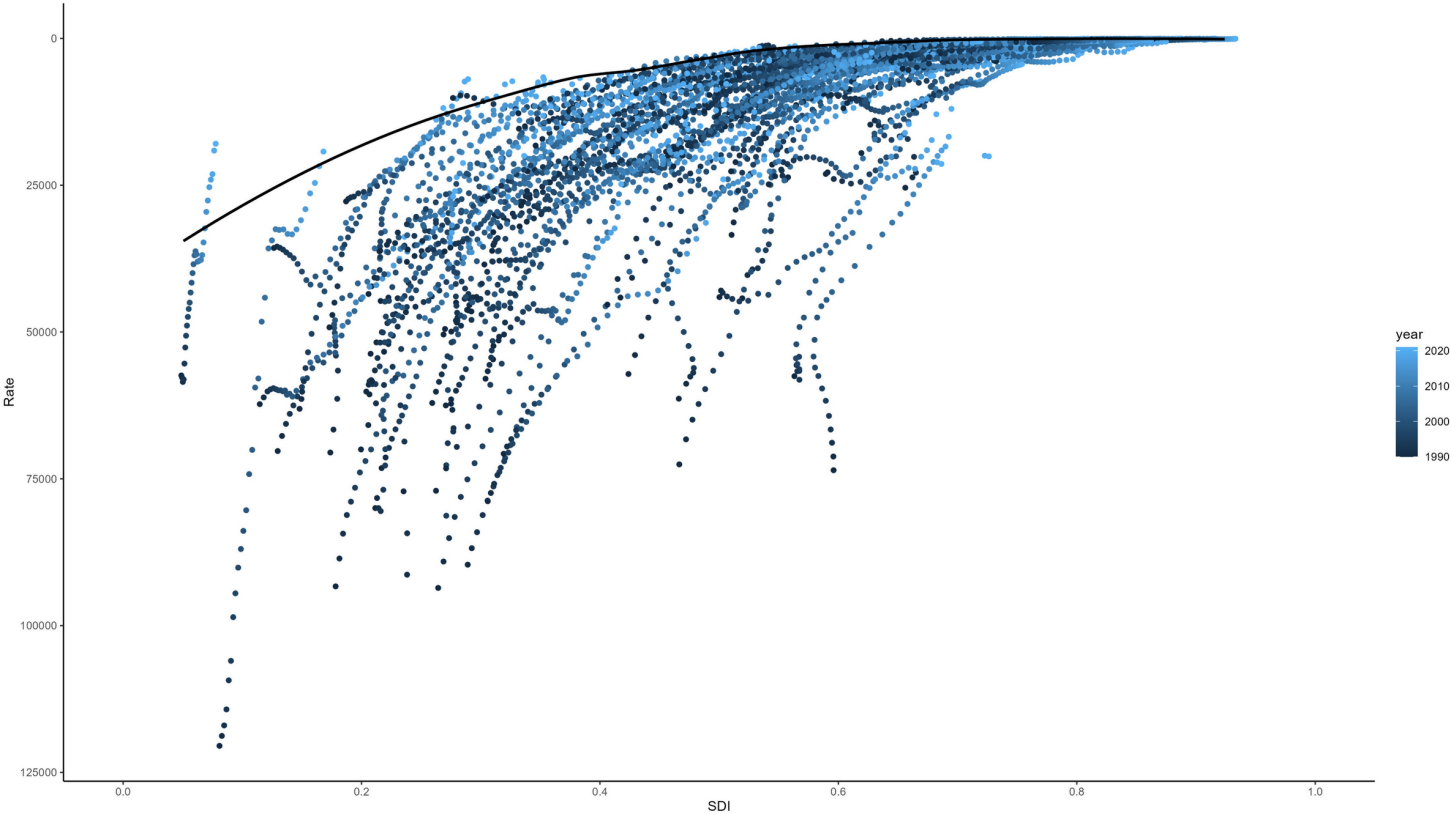
Frontier analysis of SDI and ASDR trends in LRIs in children under 5, 1990–2021. SDI, Socio-Demographic Index; ASDR, Age-Standardized DALYs Rate; LRIs, Lower Respiratory Infections.

**Figure 8:**
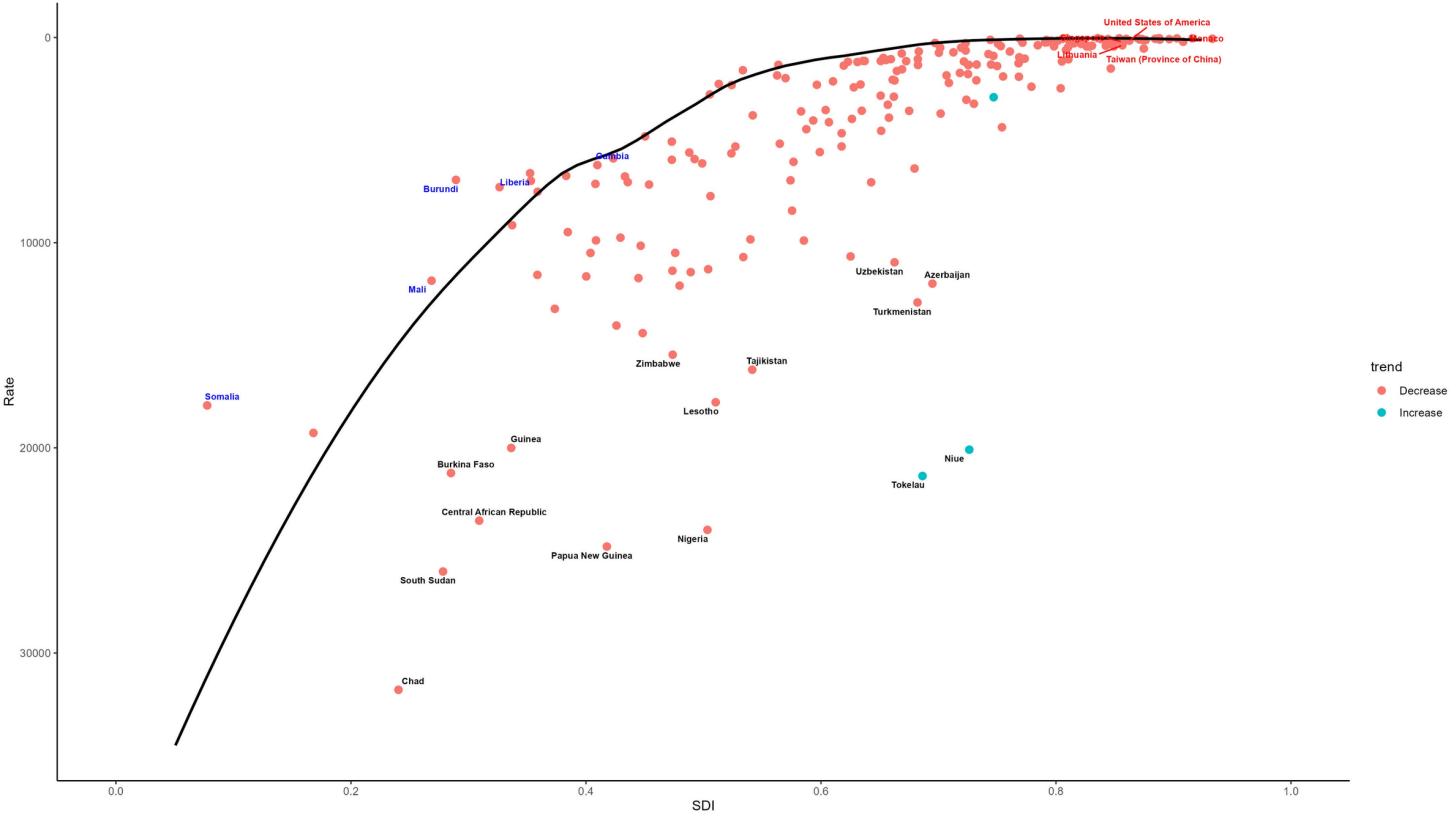
Frontier analysis of SDI and ASDR of LRIs in children under 5 in 2021. SDI, Socio-Demographic Index; ASDR, Age-Standardized DALYs Rate; LRIs, Lower Respiratory Infections.

A frontier analysis was conducted to explore the relationship between the ASDR for LRIs and the SDI in children under 5 years of age across countries from 1990 to 2021. This method identifies best-performing nations based on their SDI, highlighting disparities that can be addressed to reduce the burden of LRIs. While most countries show minimal variation in effective differences relative to their SDI, significant gaps persist in specific nations, underscoring opportunities for targeted interventions. The frontier lines derived from the analysis represent countries and regions with the lowest DALYs rates, indicating optimal performance relative to their SDIs (see Figure 7) Effective differences, defined as the gap between actual DALYs and potentially achievable DALYs, reveal disparities that could be minimized with appropriate social and population resources (see Figure 8).

In general, these effective differences are relatively small for a given SDI and exhibit limited variation as SDI increases. However, the 10 countries with the largest effective differences—ranging from 12,876.41 to 21,087.19—are Tokelau, Nigeria, Niue, Papua New Guinea, Lesotho, Tajikistan, the Central African Republic, Chad, South Sudan, and Guinea. These countries have significantly higher DALY rates for LRIs compared to peers with similar SDI levels, highlighting the need for focused efforts to improve LRIs outcomes. Conversely, the 10 countries with the lowest DALYs rates—ranging from 30.66 to 57.27—are Andorra, Cyprus, San Marino, Norway, Austria, Israel, Sweden, Germany, Spain, and Switzerland. These nations demonstrate superior performance in managing LRI-related health burdens, serving as benchmarks for others to emulate.

This figure provides a comprehensive frontier analysis of the SDI and ASDR for LRIs in children under five in 2021. The analysis identifies notable disparities across countries and regions, highlighting the largest gaps in effective differences and trends over time. Key findings emphasize the relationship between socio-economic development and LRIs burden, with color-coded markers illustrating trends and rankings. The color-coding further illustrates trends over time: a red point signifies an increase in the ASDR for LRIs in children under five from 1990 to 2021, whereas a blue point indicates a decrease over the same period. This visualization underscores the varied trajectories of LRIs burden in relation to socio-economic development. The analysis identifies the 15 countries with the highest effective differences in DALYs for LRIs, ranked from the smallest to the largest gap. These countries, such as Tokelau, Niue, Nigeria, Turkmenistan, and Azerbaijan, are marked in black on the figure. Countries with low SDIs (below 0.5) but minimal effective differences, such as Gambia, Liberia, Burundi, Mali, and Somalia, are indicated in blue. In contrast, high-SDI countries (above 0.85) with relatively high effective differences, including Monaco, the United States, Singapore, Taiwan (province of China), and Lithuania, are marked in red.

## Discussion

This study provides a comprehensive evaluation of the global burden and trends of LRIs in children under 5 years of age. The findings reveal a significant global decline in the burden of LRIs in this age group from 1990 to 2021, including a 54.02% reduction in incidence, a 37.57% reduction in mortality, and a 39.49% reduction in DALYs. However, the data also reveal notable gender disparities in the burden of LRIs, both globally and across the 21 GBD regions. Specifically, males under five consistently exhibit a higher incidence of LRIs compared to females. Despite the overall decline, the pace of progress in reducing LRI-related mortality has varied significantly across countries. Over the past 32 years, mortality and DALY rates due to LRIs in children under five have increased in certain countries, such as Niue, Tokelau, and Dominica (36). LRIs remain the leading infectious cause of death in this age group. In contrast, countries like Iran, Turkey, Saudi Arabia, and North Macedonia have experienced the largest relative decreases in the disease burden of LRIs, with the AAPC in both mortality and DALYs exceeding 10%.

The factors influencing changes in LRIs mortality rates vary significantly across countries and regions. Children under 5 years of age are widely regarded as a critical demographic for assessing the overall health and well-being of a population (37). Policymakers have long considered the mortality rate of children under five to be a key indicator of global health progress, with the reduction of child mortality as a central objective for global investment in children’s health. This study emphasizes the need for continued efforts to address disparities in the burden of LRIs and to improve global health outcomes for children (36). The risk of morbidity and mortality is significantly higher in children under five compared to other age groups. This age group is particularly susceptible to air pollutants due to the unique characteristics of their respiratory and immune systems (38). Elevated concentrations of various air pollutants, including indoor pollutants, are strongly associated with increased morbidity and mortality from LRIs (39).

To effectively mitigate the health impact of LRIs, strategies must be tailored to the unique policy environments of each country. The primary factors contributing to the decline in LRIs burden among children under five are likely associated with targeted policy interventions. For instance, Iran’s first national family planning program, launched in 1967, incorporated comprehensive maternal and child health care initiatives aimed at reducing maternal mortality, decreasing under-five mortality, preventing malnutrition, and fostering an environment conducive to child growth and development. Although the program faced temporary disruptions, it ultimately achieved nationwide coverage, benefiting more than 75% of the population. This initiative is widely regarded as one of the most successful globally, significantly improving the health outcomes of women and children (40, 41). Further strengthening these efforts, the “2021 Family Law” in Iran underscores the importance of protecting the health and rights of pregnant women and children. Along with structural economic changes and social reforms, compulsory education was established, and new family laws were introduced to enhance the legal status of women. These developments have led to improved health and living conditions, broader vaccine coverage, increased access to clean water, enhanced education for women, and ongoing improvements in healthcare systems. In rural areas, improvements in basic infrastructure and essential health services have resulted in rapid reductions in the under-five mortality rate (42). These successes underscore the critical role of well-designed and context-specific policies in reducing the LRIs burden and improving child health outcomes globally.

Turkey, one of the largest countries in both Europe and the Middle East, launched the Health Transformation Program (HTP) in 2005. The HTP rapidly expanded health insurance coverage, improved access to maternal and child health services, reduced health disparities related to poverty and socioeconomic inequality, advanced child healthcare and immunization, and significantly decreased mortality rates among children under five, infants, and newborns from infectious diseases, including LRIs (43–45).

LRIs are classified as poverty-related diseases (46). The Sustainable Development Goals (SDGs) emphasize the potential for preventing and eradicating LRIs in children under the age of five. Over 90% of countries possess the capacity to achieve these goals by optimizing their existing health systems (47). Since 1990, the global health community has made significant strides in reducing under-five mortality through interventions such as expanded immunization, improved nutrition and access to micronutrients, skilled birth attendants during delivery and postnatal care, and enhanced access to safe water and sanitation. Despite these advancements, a considerable number of children continue to die, and the burden of pediatric LRIs remains disproportionately distributed across geographic regions and income levels (36).

The relationship between the SDI and health outcomes of LRIs in children under five is strongly inverse, highlighting the beneficial effect of a higher SDI in reducing the disease burden within this age group. This phenomenon can be attributed to factors such as limited health resources, underdeveloped health infrastructure, low education levels, and high poverty rates in low-SDI countries (48). Despite improvements in global health, low-SDI countries continue to bear a disproportionate health burden (49). Through investments in resources, technological advancements, and effective health management strategies, high-SDI countries have significantly reduced disease burden, providing valuable models for others to emulate (50, 51). International health cooperation and support have been pivotal in addressing global health inequities. Organizations such as the WHO, GAVI, and the Global Fund have assisted low-SDI countries in reducing morbidity and mortality from infectious diseases, progressively narrowing global health disparities by providing vaccines, medicines, and technical support (52, 53). However, despite these advances, deeply ingrained structural challenges persist. Strengthening both international cooperation and local efforts, particularly in improving health systems in low-SDI countries, is essential. This includes enhancing health infrastructure, expanding vaccine coverage, and strengthening health worker training. Through sustained global cooperation, health outcomes in low-SDI countries can be markedly improved. Such progress is crucial for achieving the SDGs, particularly the goal of good health and well-being (54).

Frontier analysis using SDI and DALYs in children under five provides valuable insights into the global burden of LRIs from 1990 to 2021. Generally, as SDI increases, DALYs for children under five tend to decrease. However, a closer examination of the 2021 data reveals nuanced variations in DALYs across different countries. Notably, countries such as Turkmenistan, Azerbaijan, and Uzbekistan have made significant strides in reducing the burden of LRIs in children under five, bringing them closer to the optimal frontier. In contrast, countries such as Somalia, Mali, and Burundi, which face considerable social and political challenges, exhibit better-than-expected DALYs for LRIs in children under five, relative to their lower SDIs, approaching the optimal benchmark of the frontier. This variation highlights the diversity of healthcare outcomes across regions (55). While SDI is a critical factor influencing the burden of LRIs in children, other elements—such as environmental conditions, genetics, and the quality of healthcare systems—also play significant roles in shaping these outcomes (32).

Despite our efforts to provide a comprehensive analysis, the accuracy and consistency of the data across regions may vary, potentially introducing biases or inaccuracies into the results. Furthermore, as our study spans from 1990 to 2021, it may be influenced by evolving diagnostic standards and advancements in medical technology. Consequently, while our study offers valuable insights into global trends regarding the disease burden of LRIs in children under five, it is essential to interpret the findings with caution, acknowledging the potential biases that may arise in a broader context.

## Conclusion

The global burden of LRIs in children under 5 years old has declined significantly from 1990 to 2021. Additionally, there is a strong negative correlation between the burden of LRIs and the SDI at the national level. Given the high disease burden of LRIs in this age group, it is essential to address the risk factors associated with LRIs, enhance community health education, and improve vaccine coverage for vaccine-preventable diseases. These measures are crucial for further reducing the disease burden linked to LRIs in young children.

## Data Availability

Publicly available datasets were analyzed in this study. This data can be found at: https://vizhub.healthdata.org/gbd-results/.

## References

[1] Rudd KE Johnson SC Agesa KM Shackelford KA Tsoi D Kievlan DR. Global, regional, and national sepsis incidence and mortality, 1990–2017: analysis for the global burden of disease study. Lancet. (2020) 395:200–11. doi: 10.1016/S0140-6736(19)32989-7, PMID: 31954465 PMC6970225

[2] Tregoning JS Schwarze J. Respiratory viral infections in infants: causes, clinical symptoms, virology, and immunology. Clin Microbiol Rev. (2010) 23:74–98. doi: 10.1128/CMR.00032-09, PMID: 20065326 PMC2806659

[3] Vos T Lim SS Abbafati C Abbas KM Abbasi M Abbasifard M. Global burden of 369 diseases and injuries in 204 countries and territories, 1990–2019: a systematic analysis for the global burden of disease study 2019. Lancet. (2020) 396:1204–22. doi: 10.1016/S0140-6736(20)30925-9, PMID: 33069326 PMC7567026

[4] GBD 2021 Lower Respiratory Infections and Antimicrobial Resistance Collaborators. Global, regional, and national incidence and mortality burden of non-COVID-19 lower respiratory infections and aetiologies, 1990–2021: a systematic analysis from the global burden of disease study 2021. Lancet Infect Dis. (2024) 24:974–1002. doi: 10.1016/S1473-3099(24)00176-2 38636536 PMC11339187

[5] Wu M Wu Q Liu D Zu W Zhang D Chen L. The global burden of lower respiratory infections attributable to respiratory syncytial virus in 204 countries and territories, 1990-2019: findings from the global burden of disease study 2019. Intern Emerg Med. (2023) 19:59–70. doi: 10.1007/s11739-023-03438-x, PMID: 37789183

[6] Schumacher AE Kyu HH Aali A Abbafati C Abbas J Abbasgholizadeh R. Global age-sex-specific mortality, life expectancy, and population estimates in 204 countries and territories and 811 subnational locations, 1950–2021, and the impact of the COVID-19 pandemic: a comprehensive demographic analysis for the global burden of disease study 2021. Lancet. (2024) 403:1989–2056. doi: 10.1016/S0140-6736(24)00476-8, PMID: 38484753 PMC11126395

[7] Ruan Z Qi J Qian ZM Zhou M Yang Y Zhang S. Disease burden and attributable risk factors of respiratory infections in China from 1990 to 2019. Lancet Reg Health West Pac. (2021) 11:100153. doi: 10.1016/j.lanwpc.2021.100153, PMID: 34327361 PMC8315661

[8] GBD 2015 Eastern Mediterranean Region Lower Respiratory Infections Collaborators. Burden of lower respiratory infections in the eastern Mediterranean region between 1990 and 2015: findings from the global burden of disease 2015 study. Int J Public Health. (2018) 63:97–108. doi: 10.1007/s00038-017-1007-0 28776246 PMC5973986

[9] Troeger C Blacker B Khalil IA Rao PC Cao J Zimsen SRM. Estimates of the global, regional, and national morbidity, mortality, and aetiologies of lower respiratory infections in 195 countries, 1990–2016: a systematic analysis for the global burden of disease study 2016. Lancet Infect Dis. (2018) 18:1191–210. doi: 10.1016/S1473-3099(18)30310-4, PMID: 30243584 PMC6202443

[10] Li Y Wang X Blau DM Caballero MT Feikin DR Gill CJ. Global, regional, and national disease burden estimates of acute lower respiratory infections due to respiratory syncytial virus in children younger than 5 years in 2019: a systematic analysis. Lancet (London, England). (2022) 399:2047–64. doi: 10.1016/S0140-6736(22)00478-0, PMID: 35598608 PMC7613574

[11] GBD 2019 LRI Collaborators. Age-sex differences in the global burden of lower respiratory infections and risk factors, 1990-2019: results from the global burden of disease study 2019. Lancet Infect Dis. (2022) 22:1626–47. doi: 10.1016/S1473-3099(22)00510-2 35964613 PMC9605880

[12] GBD 2017 Lower Respiratory Infections Collaborators. Quantifying risks and interventions that have affected the burden of lower respiratory infections among children younger than 5 years: an analysis for the global burden of disease study 2017. Lancet Infect Dis. (2020) 20:60–79. doi: 10.1016/S1473-3099(19)30410-4 31678026 PMC7185492

[13] Ashrafi-Asgarabad A Bokaie S Razmyar J Akbarein H Nejadghaderi SA Carson-Chahhoud K. The burden of lower respiratory infections and their underlying etiologies in the Middle East and North Africa region, 1990-2019: results from the global burden of disease study 2019. BMC Pulm Med. (2023) 23:2. doi: 10.1186/s12890-022-02301-7, PMID: 36600241 PMC9811697

[14] World Health Organization, United Nations Children’s Fund (UNICEF). Ending preventable child deaths from pneumonia and diarrhoea by 2025: The integrated global action plan for pneumonia and diarrhoea (GAPPD). Geneva: World Health Organization. (2013). 56. Available at: https://iris.who.int/handle/10665/79200 (Accessed November 20, 2023)

[15] Fink G D’Acremont V Leslie HH Cohen J. Antibiotic exposure among children younger than 5 years in low-income and middle-income countries: a cross-sectional study of nationally representative facility-based and household-based surveys. Lancet Infect Dis. (2020) 20:179–87. doi: 10.1016/S1473-3099(19)30572-9, PMID: 31843383

[16] Qazi S Aboubaker S Mac Lean R Fontaine O Mantel C Goodman T. Ending preventable child deaths from pneumonia and diarrhoea by 2025. Development of the integrated global action plan for the prevention and control of pneumonia and diarrhoea. Arch Dis Child. 100:S23–8. doi: 10.1136/archdischild-2013-305429, PMID: 25613963

[17] Thomson J Chavan A. Handbook IMCI: integrated management of childhood illness. (2007). Available at: https://adc.bmj.com/content/92/2/187.2.short (Accessed November 11, 2024).

[18] Chow EJ Uyeki TM Chu HY. The effects of the COVID-19 pandemic on community respiratory virus activity. Nat Rev Microbiol. (2023) 21:195–210. doi: 10.1038/s41579-022-00807-9, PMID: 36253478 PMC9574826

[19] Chuang Y-C Lin K-P Wang L-A Yeh T-K Liu P-Y. The impact of the COVID-19 pandemic on respiratory syncytial virus infection: A narrative review. Infect Drug Resist. (2023) 16:661–75. doi: 10.2147/IDR.S396434, PMID: 36743336 PMC9897071

[20] Islam N Sharp SJ Chowell G Shabnam S Kawachi I Lacey B. Physical distancing interventions and incidence of coronavirus disease 2019: natural experiment in 149 countries. BMJ. (2020) 370:m2743. doi: 10.1136/bmj.m2743 32669358 PMC7360923

[21] Sullivan SG Carlson S Cheng AC Chilver MB Dwyer DE Irwin M. Where has all the influenza gone? The impact of COVID-19 on the circulation of influenza and other respiratory viruses, Australia, March to September 2020. Euro Surveill. (2020) 25:2001847. doi: 10.2807/1560-7917.ES.2020.25.47.2001847, PMID: 33243355 PMC7693168

[22] Murray CJL Abbafati C Abbas KM Abbasi M Abbasi-Kangevari M Abd-Allah F. Five insights from the global burden of disease study 2019. Lancet. (2020) 396:1135–59. doi: 10.1016/S0140-6736(20)31404-5, PMID: 33069324 PMC7116361

[23] Global Burden of Disease Collaborative Network. Global burden of disease study 2019 (GBD 2019) socio-demographic index (SDI) 1950–2019 (2020). doi: 10.6069/D8QB-JK35,

[24] GBD 2021 Diabetes Collaborators. Global, regional, and national burden of diabetes from. To 2021, with projections of prevalence to 2050: a systematic analysis for the global burden of disease study 2021. Lancet. (1990) 402:203–34. doi: 10.1016/S0140-6736(23)01301-6, PMID: 37356446 PMC10364581

[25] WHO. International statistical classification of diseases and related health problems 10th revision. (2016). Available at: https://icd.who.int/browse10/2016/en (Accessed June 12, 2024).

[26] Liu Z Jiang Y Yuan H Fang Q Cai N Suo C. The trends in incidence of primary liver cancer caused by specific etiologies: results from the global burden of disease study 2016 and implications for liver cancer prevention. J Hepatol. (2019) 70:674–83. doi: 10.1016/j.jhep.2018.12.001, PMID: 30543829

[27] Cao F Xu Z Li X-X Fu Z-Y Han R-Y Zhang J-L. Trends and cross-country inequalities in the global burden of osteoarthritis, 1990-2019: A population-based study. Ageing Res Rev. (2024) 99:102382. doi: 10.1016/j.arr.2024.102382, PMID: 38917934

[28] Clegg LX Hankey BF Tiwari R Feuer EJ Edwards BK. Estimating average annual per cent change in trend analysis. Stat Med. (2009) 28:3670–82. doi: 10.1002/sim.3733., PMID: 19856324 PMC2843083

[29] Cao F He YS Wang Y Zha CK Lu JM Tao LM. Global burden and cross-country inequalities in autoimmune diseases from 1990 to 2019. Autoimmun Rev. (2023) 22:103326. doi: 10.1016/j.autrev.2023.103326 36958621

[30] Arnold M Rutherford MJ Bardot A Ferlay J Andersson TM-L Myklebust TÅ. Progress in cancer survival, mortality, and incidence in seven high-income countries 1995-2014 (ICBP SURVMARK-2): a population-based study. Lancet Oncol. (2019) 20:1493–505. doi: 10.1016/S1470-2045(19)30456-5, PMID: 31521509 PMC6838671

[31] Kim HJ Fay MP Feuer EJ Midthune DN. Permutation tests for joinpoint regression with applications to cancer rates. Stat Med. (2000) 19:335–51. doi: 10.1002/(sici)1097-0258(20000215)19:3<335::aid-sim336>3.0.co;2-z, PMID: 10649300

[32] Pan H Zhao Z Deng Y Zheng Z Huang Y Huang S. The global, regional, and national early-onset colorectal cancer burden and trends from 1990 to 2019: results from the global burden of disease study 2019. BMC Public Health. (2022) 22:1896. doi: 10.1186/s12889-022-14274-7, PMID: 36221047 PMC9555189

[33] Hu J Ke R Teixeira W Dong Y Ding R Yang J. Global, regional, and National Burden of CKD due to glomerulonephritis from 1990 to 2019: A systematic analysis from the global burden of disease study 2019. Clin J Am Soc Nephrol. (2023) 18:60–71. doi: 10.2215/CJN.0000000000000017, PMID: 36719159 PMC10101559

[34] Luh J Cronk R Bartram J. Assessing Progress towards public health, human rights, and international development goals using frontier analysis. PLoS One. (2016) 11:e0147663. doi: 10.1371/journal.pone.0147663, PMID: 26812524 PMC4727803

[35] Xie Y Bowe B Mokdad AH Xian H Yan Y Li T. Analysis of the global burden of disease study highlights the global, regional, and national trends of chronic kidney disease epidemiology from 1990 to 2016. Kidney Int. (2018) 94:567–81. doi: 10.1016/j.kint.2018.04.011, PMID: 30078514

[36] Sharrow D Hug L You D Alkema L Black R Cousens S. Global, regional, and national trends in under-5 mortality between 1990 and 2019 with scenario-based projections until 2030: a systematic analysis by the UN inter-agency Group for Child Mortality Estimation. Lancet Glob Health. (2022) 10:e195–206. doi: 10.1016/S2214-109X(21)00515-5, PMID: 35063111 PMC8789561

[37] Keeley B Little C Zuehlke E. The state of the World’s children 2019: Children, food and nutrition--growing well in a changing world. United Nations Plaza, New York, NY: UNICEF (2019).

[38] He M Zhong Y Chen Y Zhong N Lai K. Association of short-term exposure to air pollution with emergency visits for respiratory diseases in children. Iscience. (2022) 25:104879. doi: 10.1016/j.isci.2022.104879, PMID: 36065191 PMC9440288

[39] Peters A Dockery DW Heinrich J Wichmann HE. Short-term effects of particulate air pollution on respiratory morbidity in asthmatic children. Eur Respir J. (1997) 10:872–9. doi: 10.1183/09031936.97.10040872 9150327

[40] Eslami M d’Arcangues C. Aiming for quality in Iran’s national family planning program - two decades of sustained efforts. Contraception. (2016) 93:209–15. doi: 10.1016/j.contraception.2015.11.013, PMID: 26593406

[41] Meidani Z Moravveji A Gohari S Ghaffarian H Zare S Vaseghi F. Development and testing requirements for an integrated maternal and child health information system in Iran: A design thinking case study. Methods Inf Med. (2022) 61:e64–72. doi: 10.1055/a-1860-8618, PMID: 35609871 PMC9788911

[42] Asadisarvestani K Sobotka T. A pronatalist turn in population policies in Iran and its likely adverse impacts on reproductive rights, health and inequality: a critical narrative review. Sex Reprod Health Matters. (2023) 31:2257075. doi: 10.1080/26410397.2023.2257075, PMID: 37830775 PMC10578100

[43] Atun R Aydın S Chakraborty S Sümer S Aran M Gürol I. Universal health coverage in Turkey: enhancement of equity. Lancet. (2013) 382:65–99. doi: 10.1016/S0140-6736(13)61051-X, PMID: 23810020

[44] Kaya ÇA Akman M Ünalan PC Çifçili S Uzuner A Akdeniz E. Comparison of preventive health service provision before and after reorganization of primary care in Turkey: a community-based study. Prim Health Care Res Dev. (2019) 20:e119. doi: 10.1017/S1463423619000069, PMID: 32323643 PMC6692006

[45] Cullu F Vural M. An overview on child health Care in Turkey. J Pediatr. (2016) 177:S213–6. doi: 10.1016/j.jpeds.2016.04.057, PMID: 27666270

[46] Li Y Nair H. Trends in the global burden of lower respiratory infections: the knowns and the unknowns. Lancet Infect Dis. (2022) 22:1523–5. doi: 10.1016/S1473-3099(22)00445-5, PMID: 35964616

[47] King C McCollum ED. Trends in the global burden of paediatric lower respiratory infections. Lancet Infect Dis. (2020) 20:4–5. doi: 10.1016/S1473-3099(19)30557-2, PMID: 31678028 PMC7952014

[48] Bhutta ZA Sommerfeld J Lassi ZS Salam RA Das JK. Global burden, distribution, and interventions for infectious diseases of poverty. Infect Dis Poverty. (2014) 3:21. doi: 10.1186/2049-9957-3-21, PMID: 25110585 PMC4126350

[49] Cheng F Xiao J Shao C Huang F Wang L Ju Y. Burden of thyroid Cancer from 1990 to 2019 and projections of incidence and mortality until 2039 in China: findings from global burden of disease study. Front Endocrinol (Lausanne). (2021) 12:738213. doi: 10.3389/fendo.2021.738213, PMID: 34690931 PMC8527095

[50] Koch AM. Missing the Care in Health Care. Am J Nurs. (2021) 121:48–52. doi: 10.1097/01.NAJ.0000737300.54432.87, PMID: 33625011

[51] Safiri S Kolahi AA Hoy D Smith E Bettampadi D Mansournia MA. Global, regional and national burden of rheumatoid arthritis 1990-2017: a systematic analysis of the global burden of disease study 2017. Ann Rheum Dis. (2019) 78:1463–71. doi: 10.1136/annrheumdis-2019-215920, PMID: 31511227

[52] Schwalbe N. The global alliance for vaccines and immunization (GAVI). Vaccine. (2012) 30:x. doi: 10.1016/S0264-410X(12)01438-7 23510771

[53] Head MG Brown RJ Newell M-L Scott JAG Batchelor J Atun R. The allocation of US$105 billion in global funding from G20 countries for infectious disease research between 2000 and 2017: a content analysis of investments. Lancet Glob Health. (2020) 8:e1295–304. doi: 10.1016/S2214-109X(20)30357-0, PMID: 32971052 PMC7505652

[54] Kruk ME Gage AD Arsenault C Jordan K Leslie HH Roder-DeWan S. High-quality health systems in the sustainable development goals era: time for a revolution. Lancet Glob Health. (2018) 6:e1196–252. doi: 10.1016/S2214-109X(18)30386-3, PMID: 30196093 PMC7734391

[55] Geng J Zhao J Fan R Zhu Z Zhang Y Zhu Y. Global, regional, and national burden and quality of care of multiple myeloma, 1990-2019. J Glob Health. (2024) 14:04033. doi: 10.7189/jogh.14.04033, PMID: 38299781 PMC10832550

