# Supplementary material for: A 32-year trend analysis of lower respiratory infections in children under 5: insights from the global burden of disease study 2021
Source: Front Public Health. 2025 Jan 22;13:1483179. doi: 10.3389/fpubh.2025.1483179 (PMC11794078; doi:10.3389/fpubh.2025.1483179)
Supplement: Supplementary file 1 [file Supplementary_file_1.docx]

Table S1 Incidence, mortality, DALYs cases, ARSs, and AAPC values in 204 countries/territories in 2021

| Characteristic | Incidence cases（95%UI） | ASIR per 100,000 (95% UI) | Percentage change （95%CI） | ASIR AAPC1990-2021(%) | Mortality cases（95%UI） | ASMR per 100,000 (95% UI) | Percentage change （95%CI） | ASMR AAPC1990-2021(%) | DALYs cases（95%UI） | ASDR per 100,000 (95% UI) | Percentage change （95%CI） | ASDR AAPC1990-2021(%) |
| --- | --- | --- | --- | --- | --- | --- | --- | --- | --- | --- | --- | --- |
| Afghanistan | 303,160.51(260,420.81~353,904.14) | 5,530.67(4,750.95~6,456.4) | -59.22(-63.91~-53.8) | -4(-4.07~-3.92) | 5,608.45(4,099.12~7,666.14) | 102.32(74.78~139.86) | -51.84(-63.11~-38.23) | -6.37(-6.64~-6.1) | 501,426.31(366,494.23~685,287.45) | 9,147.71(6,686.09~12,501.95) | -52.96(-63.97~-39.74) | -6.36(-6.62~-6.1) |
| Albania | 3,386.56(2,961.84~3,891.3) | 2,374.82(2,076.98~2,728.77) | -79.47(-81.67~-76.86) | -5.38(-5.62~-5.14) | 29.51(20.3~40.35) | 20.69(14.23~28.29) | -75.93(-82.99~-67.27) | -7.81(-8.28~-7.33) | 2,632.99(1,812.95~3,596.4) | 1,846.38(1,271.33~2,521.97) | -77.34(-83.95~-69.16) | -7.82(-8.29~-7.34) |
| Algeria | 96,438.06(81,025.48~115,986.5) | 2,049.84(1,722.23~2,465.35) | -65.73(-70.32~-60.19) | -4.44(-4.52~-4.35) | 554.35(387.71~745.94) | 11.78(8.24~15.86) | -61.5(-73.94~-44.73) | -6.96(-7.25~-6.68) | 49,624.49(34,733.48~66,699.66) | 1,054.79(738.28~1,417.73) | -63.56(-75.23~-48.7) | -6.96(-7.25~-6.68) |
| American Samoa | 136.11(117.13~158.76) | 3,669.63(3,157.91~4,280.16) | -57.02(-61.52~-52.71) | -2.59(-2.68~-2.5) | 1.26(0.9~1.65) | 33.98(24.15~44.53) | -20.3(-35.71~-4.49) | -2.22(-2.48~-1.95) | 112.56(80.18~147.34) | 3,034.75(2,161.56~3,972.42) | -24.53(-39.79~-8.38) | -2.22(-2.48~-1.96) |
| Andorra | 12.57(10.11~15.35) | 497.91(400.44~608.16) | -65(-68.58~-60.81) | -3.25(-3.4~-3.09) | 0.01(0~0.01) | 0.34(0.19~0.5) | -68.24(-80.67~-50.86) | -9.1(-10.05~-8.13) | 0.77(0.43~1.14) | 30.66(17.08~45.25) | -82.36(-89.64~-70.36) | -9.05(-10~-8.1) |
| Angola | 293,937.45(252,268.89~340,834.95) | 5,217.95(4,478.25~6,050.47) | -54.59(-60.1~-47.64) | -3.97(-4.03~-3.91) | 4,530.59(3,084.84~6,479.4) | 80.43(54.76~115.02) | -48.94(-64.93~-28.59) | -6.78(-7.05~-6.5) | 403,684.92(274,859.17~575,970.42) | 7,166.18(4,879.27~10,224.57) | -50.11(-65.75~-30.22) | -6.76(-7.04~-6.49) |
| Antigua and Barbuda | 145.35(124.43~170.27) | 2,757.04(2,360.13~3,229.61) | -51.84(-56.18~-47) | -2.45(-2.64~-2.26) | 0.82(0.69~0.96) | 15.54(13.13~18.16) | 3.03(-7.25~13.95) | -1.03(-1.56~-0.49) | 73.32(62.04~85.68) | 1,390.64(1,176.67~1,625.21) | -0.36(-10.69~10.42) | -1.04(-1.56~-0.5) |
| Argentina | 65,887.52(54,148.38~79,989.43) | 2,198.99(1,807.2~2,669.64) | -47.55(-54.85~-40.03) | -2.75(-3.24~-2.25) | 211.31(170.26~259.3) | 7.05(5.68~8.65) | -40.83(-48.27~-33.71) | -4.21(-4.98~-3.44) | 18,948.87(15,258.78~23,255.91) | 632.42(509.26~776.16) | -46.57(-53.22~-39.73) | -4.21(-4.98~-3.44) |
| Armenia | 4,911.1(3,945.6~6,093.3) | 2,637.42(2,118.91~3,272.29) | -64.57(-70.61~-56.93) | -3.6(-3.72~-3.47) | 77.25(60.25~99.28) | 41.48(32.36~53.32) | -30.44(-38.71~-19.93) | -5.15(-5.78~-4.52) | 6,904.98(5,390.25~8,867.39) | 3,708.19(2,894.74~4,762.07) | -38.02(-45.82~-28.11) | -5.16(-5.78~-4.52) |
| Australia | 6,573.61(5,448.6~7,916.26) | 437.23(362.41~526.54) | -66.12(-69.13~-62.55) | -3.73(-4.05~-3.41) | 12.89(10.19~15.84) | 0.86(0.68~1.05) | -52.01(-60.1~-43.72) | -5.95(-8.5~-3.33) | 1,162.29(921.11~1,424.4) | 77.31(61.27~94.74) | -61.58(-68.92~-54.3) | -5.94(-8.47~-3.34) |
| Austria | 2,640.82(2,136.85~3,217.17) | 611.13(494.51~744.51) | -56.41(-61.66~-49.92) | -3.03(-3.13~-2.94) | 1.91(1.61~2.22) | 0.44(0.37~0.51) | -60.03(-65.04~-54.86) | -6.55(-8.64~-4.43) | 174.04(146.85~201.69) | 40.28(33.98~46.67) | -67.62(-71.73~-62.68) | -6.51(-8.55~-4.43) |
| Azerbaijan | 29,784.2(26,544.5~33,417.21) | 4,130.69(3,681.39~4,634.55) | -71.15(-73.71~-68.06) | -4.24(-4.5~-3.98) | 966.62(761.56~1,220.78) | 134.06(105.62~169.31) | -51.35(-58.18~-41.8) | -5.55(-5.94~-5.15) | 86,497.36(68,171.36~109,192.24) | 11,996.1(9,454.51~15,143.59) | -53.46(-59.99~-44.01) | -5.55(-5.94~-5.15) |
| Bahamas | 413.52(348.04~486.36) | 1,963.95(1,652.98~2,309.92) | -68.34(-71.51~-65.18) | -3.74(-3.9~-3.58) | 2.74(2~3.75) | 13.01(9.49~17.8) | -33.29(-41.03~-24.79) | -4.22(-6.21~-2.19) | 245.68(179.36~335.61) | 1,166.83(851.83~1,593.95) | -39.5(-47.53~-30.7) | -4.22(-6.21~-2.19) |
| Bahrain | 1,648.64(1,323.77~2,025.53) | 1,759.67(1,412.93~2,161.94) | -57.85(-63.2~-51.2) | -2.9(-2.97~-2.82) | 4.38(3.24~5.7) | 4.67(3.46~6.09) | -52.74(-64.68~-38.46) | -7.13(-8.11~-6.14) | 393.12(292.3~511.26) | 419.59(311.98~545.7) | -60.05(-70.14~-47) | -7.12(-8.09~-6.13) |
| Bangladesh | 927,799.32(798,011.64~1081,163.3) | 6,459.17(5,555.61~7,526.86) | -67.96(-72.4~-62.97) | -5.19(-5.54~-4.84) | 9,511.46(6,988.8~12,564.09) | 66.22(48.65~87.47) | -33.37(-49.44~-14.61) | -6.5(-7.03~-5.97) | 851,143.78(625,638.41~1124,145.86) | 5,925.51(4,355.58~7,826.1) | -36.56(-52.21~-18.75) | -6.49(-7.02~-5.96) |
| Barbados | 354.01(296.08~416.76) | 2,599.74(2,174.32~3,060.57) | -59.41(-63.75~-54.69) | -2.95(-3.19~-2.71) | 1.35(0.93~1.92) | 9.95(6.82~14.07) | -32.81(-38.47~-26.09) | -2.04(-2.65~-1.43) | 121.77(83.67~171.93) | 894.26(614.43~1,262.6) | -36.48(-43.49~-28.95) | -2.03(-2.65~-1.42) |
| Belarus | 13,315.17(10,687.94~16,491.82) | 2,849.03(2,286.88~3,528.73) | -64.79(-70.91~-56.01) | -3.62(-3.97~-3.27) | 19.58(14.44~26.01) | 4.19(3.09~5.57) | -52.39(-58.75~-45.1) | -7.29(-8.49~-6.07) | 1,769.42(1,311.13~2,350.93) | 378.6(280.54~503.03) | -61.78(-68.28~-54.76) | -7.27(-8.47~-6.05) |
| Belgium | 3,343.31(2,712.86~4,127.63) | 564.81(458.3~697.31) | -58.26(-62.98~-52.85) | -2.72(-2.99~-2.46) | 7.44(5.83~9.21) | 1.26(0.98~1.56) | -47.46(-54.24~-39.37) | -5.46(-7.41~-3.47) | 669.63(524.41~828.51) | 113.12(88.59~139.97) | -55.68(-61.95~-47.88) | -5.44(-7.39~-3.45) |
| Belize | 903.44(770.13~1,058.6) | 2,369.24(2,019.64~2,776.13) | -63.33(-67.82~-54.7) | -3.66(-3.72~-3.6) | 9.1(7.28~11.18) | 23.86(19.09~29.33) | -26.12(-32.7~-18.71) | -4.68(-5.21~-4.15) | 814.9(652.22~1,001.08) | 2,137.05(1,710.43~2,625.28) | -31.71(-38.52~-24.25) | -4.68(-5.21~-4.14) |
| Benin | 166,957.46(145,306.62~193,598.13) | 7,127.74(6,203.42~8,265.08) | -33.99(-41.37~-25.22) | -2.57(-2.65~-2.49) | 3,485.5(2,400.31~4,976.85) | 148.8(102.47~212.47) | -40.25(-55.17~-19.89) | -4.43(-4.93~-3.94) | 309,757.59(213,861.82~441,736.02) | 13,224.15(9,130.17~18,858.57) | -41.12(-55.7~-21.02) | -4.43(-4.92~-3.94) |
| Bermuda | 66.77(55.52~79.28) | 2,617.23(2,176.23~3,107.44) | -54.05(-58.79~-49.08) | -2.48(-2.68~-2.27) | 0.09(0.06~0.11) | 3.47(2.43~4.39) | -16.21(-36.89~-1.26) | -3.87(-4.48~-3.26) | 7.96(5.61~10.08) | 312.12(219.85~395.02) | -32.32(-48.76~-19.14) | -3.87(-4.47~-3.26) |
| Bhutan | 4,387.51(3,785.15~5,190.96) | 7,189.06(6,202.07~8,505.53) | -50.6(-61.35~-29.78) | -4.43(-4.68~-4.19) | 34.66(22.95~48.12) | 56.79(37.6~78.84) | -31.24(-57.88~20.1) | -5.92(-6.56~-5.28) | 3,098(2,056.12~4,294.7) | 5,076.16(3,369~7,036.98) | -35.72(-60.84~11.73) | -5.91(-6.54~-5.28) |
| Bolivia (Plurinational State of) | 51,938.73(44,467.64~60,606.26) | 4,349.3(3,723.68~5,075.11) | -72.58(-76.4~-68.62) | -5.18(-5.55~-4.82) | 746.92(550.41~960.88) | 62.55(46.09~80.46) | -57.91(-67.03~-43.99) | -7.28(-7.56~-6.99) | 66,672.64(49,152.2~85,717.42) | 5,583.11(4,115.96~7,177.9) | -60.08(-68.73~-46.74) | -7.27(-7.56~-6.98) |
| Bosnia and Herzegovina | 3,039.39(2,502.71~3,692.22) | 2,012.31(1,656.98~2,444.52) | -65.5(-69.93~-60.51) | -3.46(-3.58~-3.33) | 4.51(3.23~6.12) | 2.99(2.14~4.06) | -28.81(-51.25~1.83) | -4.55(-5.76~-3.33) | 407.94(293.35~552.29) | 270.09(194.22~365.66) | -39.59(-58.22~-13.53) | -4.54(-5.74~-3.33) |
| Botswana | 18,364.9(15,749.98~21,536.18) | 7,798.32(6,687.94~9,144.95) | -29.69(-37.66~-21.61) | -1.87(-2.37~-1.36) | 186.88(125.78~271.42) | 79.36(53.41~115.25) | -17.18(-37.17~5.28) | -2.32(-2.71~-1.92) | 16,619.36(11,212~24,120.82) | 7,057.11(4,760.97~10,242.47) | -18.45(-37.77~3.19) | -2.32(-2.71~-1.93) |
| Brazil | 784,188.87(665,988.88~924,644.01) | 4,736.14(4,022.27~5,584.43) | -61.61(-65.45~-57.06) | -3.49(-4~-2.98) | 1,835.32(1,387.74~2,328.23) | 11.08(8.38~14.06) | -69.62(-74.22~-64.33) | -8.3(-8.74~-7.86) | 164,760.25(124,776.64~208,708.9) | 995.08(753.59~1,260.51) | -72.46(-76.94~-67.54) | -8.29(-8.73~-7.85) |
| Brunei Darussalam | 766.15(666.94~885.46) | 2,479.83(2,158.71~2,866) | -48.75(-53.66~-43.24) | -2.21(-2.26~-2.17) | 1.63(1.2~2.23) | 5.28(3.88~7.21) | -53.53(-62.69~-40.68) | -3.09(-3.53~-2.65) | 146.89(108.24~200.04) | 475.45(350.34~647.48) | -54.02(-63.14~-41.38) | -3.07(-3.51~-2.64) |
| Bulgaria | 6,013.68(5,242.98~6,982.79) | 1,986.8(1,732.18~2,306.98) | -80.52(-82.92~-77.34) | -5.02(-5.18~-4.85) | 42.48(34.15~50.36) | 14.03(11.28~16.64) | -56.27(-61.71~-50.64) | -5.91(-8~-3.78) | 3,804.6(3,060.97~4,506.99) | 1,256.96(1,011.28~1,489.02) | -61.62(-67.21~-56.14) | -5.91(-7.99~-3.78) |
| Burkina Faso | 366,985.3(318,626.23~421,224.35) | 8,936.77(7,759.14~10,257.59) | -21.06(-30.41~-10.04) | -2.29(-2.46~-2.12) | 9,795.49(6,815.49~12,766.6) | 238.54(165.97~310.89) | -29.45(-45.06~-10.78) | -3.81(-4.2~-3.41) | 871,812.17(607,262.21~1136,207.1) | 21,230.23(14,787.96~27,668.74) | -30.57(-45.67~-12.46) | -3.8(-4.19~-3.41) |
| Burundi | 148,764.96(128,669.97~173,448.87) | 6,886.03(5,955.88~8,028.6) | -50.31(-56.72~-43.77) | -3.24(-3.48~-2.99) | 1,678.5(1,018.29~2,529.82) | 77.69(47.13~117.1) | -49.93(-64.66~-35.8) | -5.83(-6.07~-5.58) | 149,919.61(91,160.13~225,885.08) | 6,939.48(4,219.62~10,455.77) | -50.89(-65.17~-37.03) | -5.81(-6.06~-5.56) |
| Cabo Verde | 1,619.5(1,398.16~1,871.56) | 3,675.73(3,173.37~4,247.83) | -49.88(-56.08~-43.34) | -3.83(-4.05~-3.6) | 7.85(5.02~11) | 17.81(11.39~24.97) | -25.66(-52.14~10.21) | -5.79(-6.65~-4.92) | 703.34(450.72~985.39) | 1,596.35(1,022.99~2,236.52) | -31.69(-57.14~-0.24) | -5.79(-6.64~-4.92) |
| Cambodia | 107,672.16(93,506.35~124,100.18) | 6,157.81(5,347.66~7,097.34) | -68.25(-72.06~-64.27) | -4.93(-5.01~-4.84) | 2,222.64(1,679.82~2,815.05) | 127.11(96.07~160.99) | -43.72(-53.46~-32.95) | -6.33(-6.58~-6.09) | 198,791.59(150,234.13~251,958.84) | 11,368.97(8,591.95~14,409.62) | -45.94(-55.54~-35.39) | -6.32(-6.56~-6.08) |
| Cameroon | 327,783.7(283,056.31~373,645.2) | 6,739.37(5,819.76~7,682.3) | -23.41(-32.61~-12.21) | -2.23(-2.5~-1.97) | 6,615.9(4,686.92~8,901.91) | 136.03(96.37~183.03) | -28.81(-45.65~-6.75) | -3.68(-3.99~-3.38) | 588,290.87(416,908.88~790,522.56) | 12,095.51(8,571.82~16,253.48) | -29.82(-46.38~-8.5) | -3.68(-3.98~-3.37) |
| Canada | 13,485.36(11,350.28~16,085.21) | 709.72(597.35~846.55) | -59.74(-63.98~-55.47) | -2.94(-3.1~-2.79) | 15.91(13.05~19.02) | 0.84(0.69~1) | -62.83(-68.13~-56.45) | -5.4(-7.16~-3.62) | 1,441.21(1,182.48~1,716.52) | 75.85(62.23~90.34) | -67.37(-72.03~-61.96) | -5.38(-7.13~-3.61) |
| Central African Republic | 87,450.86(75,140.12~101,474.92) | 10,425.18(8,957.6~12,097.02) | -26.32(-35.48~-16.31) | -1.95(-2~-1.9) | 2,218.33(1,405.78~3,186.68) | 264.45(167.59~379.89) | -38.87(-54.1~-20.91) | -3.58(-3.74~-3.43) | 197,534.29(125,418.13~283,205.5) | 23,548.44(14,951.33~33,761.46) | -39.56(-54.55~-21.93) | -3.58(-3.74~-3.43) |
| Chad | 432,250.77(377,521.36~488,145.26) | 11,889.67(10,384.26~13,427.13) | -15.17(-23.38~-6.1) | -1.63(-1.68~-1.57) | 13,026.28(9,639.48~16,798.73) | 358.31(265.15~462.07) | -1.9(-24.56~26.14) | -2.17(-2.51~-1.83) | 1155,867.12(855,050.4~1489,572.18) | 31,793.76(23,519.37~40,972.8) | -2.84(-25.15~24.68) | -2.17(-2.5~-1.84) |
| Chile | 15,083.27(12,336.21~18,299.96) | 1,386.38(1,133.88~1,682.04) | -75.31(-79.59~-70.3) | -4.95(-5.13~-4.78) | 30.32(26.16~34.96) | 2.79(2.4~3.21) | -80.85(-82.58~-78.87) | -9.42(-11.86~-6.91) | 2,728.44(2,365.16~3,146.93) | 250.78(217.39~289.25) | -83.73(-85.32~-81.97) | -9.41(-11.82~-6.93) |
| China | 2398,985.19(1997,575.87~2834,649.53) | 3,088.76(2,571.93~3,649.69) | -74.89(-78.28~-71.57) | -5.29(-5.38~-5.21) | 10,116.26(8,015.55~12,493.84) | 13.02(10.32~16.09) | -53.63(-64.58~-44.83) | -9.53(-9.99~-9.06) | 907,895.03(719,150.88~1120,026.16) | 1,168.94(925.93~1,442.06) | -59.95(-69.36~-51.95) | -9.51(-9.98~-9.04) |
| Colombia | 97,371.48(82,323.33~115,528.65) | 2,828.29(2,391.2~3,355.69) | -72.19(-77.01~-67.1) | -4.37(-4.6~-4.15) | 420.83(288.57~602.1) | 12.22(8.38~17.49) | -61.04(-66.05~-54.99) | -6.6(-7.32~-5.88) | 37,745.39(25,911.91~53,912.69) | 1,096.37(752.65~1,565.97) | -63.7(-69.17~-57.66) | -6.59(-7.3~-5.87) |
| Comoros | 5,343.91(4,624.04~6,151.78) | 6,572.51(5,687.14~7,566.11) | -48.65(-55.63~-34.36) | -3.48(-3.99~-2.97) | 95.79(70.86~124.89) | 117.81(87.16~153.6) | -37.13(-52.47~-15.56) | -4.89(-5.58~-4.2) | 8,535.58(6,318.89~11,146.06) | 10,497.96(7,771.64~13,708.61) | -38.97(-53.96~-18.49) | -4.89(-5.58~-4.2) |
| Congo | 26,475.85(22,254.34~30,939.83) | 4,183.64(3,516.57~4,889.02) | -54.13(-59.6~-47.09) | -3.41(-3.56~-3.26) | 255.29(169.43~355.29) | 40.34(26.77~56.14) | -53.79(-66.02~-37.69) | -5.77(-6.01~-5.53) | 22,798.73(15,120.3~31,700.57) | 3,602.59(2,389.27~5,009.24) | -55.33(-67.08~-39.37) | -5.76(-6~-5.53) |
| Cook Islands | 31.25(25.77~37.51) | 2,779.71(2,291.95~3,336.14) | -79.9(-82.5~-77.14) | -5.15(-5.21~-5.08) | 0.3(0.21~0.37) | 26.93(18.55~32.56) | 1.15(-37.74~30.1) | -4.55(-5.73~-3.35) | 26.98(18.6~32.58) | 2,399.46(1,654.4~2,897.31) | -15.53(-49.64~8.62) | -4.56(-5.73~-3.37) |
| Costa Rica | 6,199.53(5,151.35~7,471.71) | 2,010.87(1,670.88~2,423.51) | -63.7(-67.84~-59.21) | -3.5(-4.03~-2.98) | 25.53(20.53~31.19) | 8.28(6.66~10.12) | -53.82(-61.5~-44.77) | -5.11(-6.85~-3.35) | 2,289.52(1,842.44~2,794.83) | 742.62(597.61~906.52) | -57.25(-64.61~-48.4) | -5.11(-6.84~-3.35) |
| Coted'Ivoire | 323,235.07(283,638.93~368,793.3) | 7,423.2(6,513.86~8,469.46) | -21.8(-31.53~-11.6) | -2.15(-2.31~-2) | 6,860.07(4,733.2~9,382.76) | 157.54(108.7~215.48) | -27.5(-41.91~-8.84) | -3.59(-4.03~-3.15) | 611,281.62(422,697.79~835,636.75) | 14,038.28(9,707.39~19,190.67) | -28.89(-42.87~-10.56) | -3.59(-4.03~-3.16) |
| Croatia | 2,466.63(2,018.42~2,987.76) | 1,353.45(1,107.51~1,639.39) | -62.18(-66.17~-57.55) | -2.94(-3.08~-2.81) | 8.71(6.41~11.12) | 4.78(3.52~6.1) | -32.38(-44.25~-19.32) | -3.75(-5.07~-2.41) | 781.59(575.61~997.21) | 428.86(315.84~547.17) | -40.95(-51.98~-28.81) | -3.75(-5.06~-2.41) |
| Cuba | 11,912.67(10,160.18~14,066.92) | 2,187.96(1,866.08~2,583.62) | -66.39(-70.65~-61.51) | -3.5(-3.59~-3.42) | 47.2(37.98~56.78) | 8.67(6.97~10.43) | 5.62(-6.74~17.68) | -3.33(-5.78~-0.82) | 4,236.84(3,412.24~5,087.17) | 778.16(626.71~934.34) | -15.16(-27.34~-1.85) | -3.33(-5.76~-0.84) |
| Cyprus | 331.62(269.91~405.18) | 441.91(359.68~539.94) | -62.27(-65.97~-57.93) | -3.02(-3.11~-2.94) | 0.28(0.19~0.41) | 0.38(0.25~0.54) | -67.88(-82.95~-47.85) | -9.05(-9.4~-8.69) | 25.66(17.48~36.94) | 34.19(23.29~49.23) | -76.37(-87.4~-62.22) | -9(-9.35~-8.65) |
| Czechia | 7,710.55(6,410.58~9,319.39) | 1,371.82(1,140.54~1,658.06) | -65.78(-68.99~-61.92) | -3.15(-3.39~-2.91) | 27.53(21.74~33.19) | 4.9(3.87~5.91) | -15.51(-25.37~-5.71) | -5.33(-6.45~-4.19) | 2,471.11(1,957.6~2,975.91) | 439.65(348.29~529.46) | -38.04(-48.74~-28.24) | -5.32(-6.43~-4.19) |
| Democratic People's Republic of Korea | 58,627.64(49,687.37~69,596.84) | 3,874.62(3,283.77~4,599.56) | -77.67(-80~-75.34) | -5.05(-5.13~-4.96) | 335.11(202.81~474.31) | 22.15(13.4~31.35) | -49.51(-67.28~-34.82) | -6.03(-6.27~-5.79) | 29,977.47(18,143.39~42,416.82) | 1,981.17(1,199.07~2,803.27) | -53.07(-69.34~-38.82) | -6.04(-6.28~-5.8) |
| Democratic Republic of the Congo | 761,063.69(644,951.08~901,099.92) | 5,612.26(4,756.02~6,644.92) | -58.32(-63.18~-52.36) | -3.79(-3.87~-3.7) | 10,248.93(6,968.49~15,051.97) | 75.58(51.39~111) | -52.88(-64.37~-38.97) | -6.06(-6.23~-5.89) | 914,374.98(623,052.57~1339,542.58) | 6,742.81(4,594.53~9,878.09) | -53.91(-65.27~-40.32) | -6.05(-6.22~-5.88) |
| Denmark | 1,610.58(1,318.34~1,971.78) | 518.38(424.32~634.64) | -59.17(-63.46~-54.56) | -2.8(-2.89~-2.72) | 2.77(2.24~3.36) | 0.89(0.72~1.08) | -62.17(-68.9~-55.32) | -5.92(-6.82~-5.02) | 250.08(202.65~302.38) | 80.49(65.23~97.32) | -67.89(-73.61~-61.96) | -5.9(-6.78~-5) |
| Djibouti | 10,946.39(9,477.92~12,621.52) | 7,513.18(6,505.28~8,662.93) | -32.89(-40.84~-25.57) | -2.54(-2.81~-2.28) | 91.61(60.39~134.27) | 62.88(41.45~92.16) | -37.93(-56.29~-15.1) | -4.88(-5.46~-4.29) | 8,168.5(5,381.15~11,963.87) | 5,606.55(3,693.42~8,211.55) | -40.31(-57.89~-18.24) | -4.88(-5.46~-4.3) |
| Dominica | 93.81(80.43~109.79) | 2,696.8(2,312.24~3,156.35) | -44.35(-49.33~-39.48) | -1.96(-2~-1.92) | 1.13(0.78~1.63) | 32.53(22.39~46.94) | -15.83(-33.52~7.02) | 0.75(0.19~1.31) | 101.22(69.68~145.94) | 2,909.93(2,003.1~4,195.62) | -14.01(-31.7~10.12) | 0.74(0.18~1.31) |
| Dominican Republic | 25,882.03(22,043.55~30,230.58) | 2,505.22(2,133.68~2,926.13) | -69.63(-73.75~-65.42) | -4.62(-4.72~-4.52) | 157.89(99.23~226.01) | 15.28(9.6~21.88) | -65.06(-78.64~-51.25) | -6.53(-7.67~-5.37) | 14,155.29(8,921.33~20,244.48) | 1,370.15(863.53~1,959.54) | -66.28(-79.28~-53.09) | -6.52(-7.66~-5.36) |
| Ecuador | 50,607.33(42,341.16~59,877.22) | 3,046.57(2,548.95~3,604.62) | -61.36(-66.42~-55.25) | -4.38(-4.52~-4.23) | 382.57(266.87~527.79) | 23.03(16.07~31.77) | -46.84(-54.55~-37.96) | -5.87(-6.99~-4.73) | 34,173.22(23,874.18~47,117.19) | 2,057.23(1,437.23~2,836.46) | -49.73(-57.64~-40.83) | -5.87(-6.99~-4.74) |
| Egypt | 394,030.54(327,347.76~471,888.86) | 3,022.31(2,510.84~3,619.5) | -67.99(-73.05~-62.46) | -4.99(-5.09~-4.89) | 6,014.93(4,600.48~7,793.54) | 46.14(35.29~59.78) | -31.67(-47.77~-12.94) | -7.39(-8~-6.78) | 538,250.66(411,607.17~697,734.21) | 4,128.51(3,157.13~5,351.79) | -38.06(-52.7~-20.88) | -7.38(-7.99~-6.77) |
| El Salvador | 16,108.11(13,516.43~19,142.68) | 2,681.43(2,250.01~3,186.58) | -66.92(-71.07~-62.45) | -4.64(-5.51~-3.77) | 89.38(58.47~128.04) | 14.88(9.73~21.31) | -33.39(-52.05~-12.71) | -7.18(-7.58~-6.79) | 8,009.97(5,240.83~11,459.74) | 1,333.38(872.41~1,907.64) | -40.91(-57.98~-23.73) | -7.18(-7.57~-6.78) |
| Equatorial Guinea | 6,016.74(5,137.41~7,049.98) | 3,211.7(2,742.32~3,763.23) | -67.49(-72.16~-62.53) | -4.98(-5.09~-4.87) | 81.91(47.32~138.14) | 43.72(25.26~73.74) | -67.32(-78.2~-50.05) | -7.98(-8.74~-7.22) | 7,324.81(4,244.34~12,351.29) | 3,909.93(2,265.6~6,593.04) | -68.29(-78.71~-51.66) | -7.97(-8.72~-7.2) |
| Eritrea | 79,428.7(69,098.32~92,143.72) | 8,652.02(7,526.75~10,037.05) | -41.06(-47.56~-34.05) | -3.1(-3.35~-2.86) | 1,086.5(752.15~1,496.43) | 118.35(81.93~163) | -14.9(-35.34~12.53) | -4.63(-5.14~-4.12) | 96,402.11(66,827.3~132,669.12) | 10,500.9(7,279.38~14,451.4) | -17.93(-37.9~8.47) | -4.63(-5.14~-4.12) |
| Estonia | 1,416(1,173.8~1,714.55) | 2,046.97(1,696.84~2,478.55) | -67.38(-71.8~-62.55) | -3.78(-3.88~-3.69) | 2.29(1.93~2.71) | 3.31(2.79~3.91) | -30.47(-39.57~-20.33) | -7.09(-7.89~-6.28) | 206.71(174.59~243.7) | 298.82(252.38~352.29) | -51.43(-59.45~-43.1) | -7.07(-7.87~-6.26) |
| Eswatini | 9,257.36(8,064.56~10,686.18) | 6,592.8(5,743.33~7,610.37) | -34.61(-42.28~-26.81) | -2.62(-3.17~-2.08) | 155.52(110.74~209.9) | 110.76(78.87~149.48) | -10.41(-30.84~16.92) | -2.68(-3.11~-2.26) | 13,897.66(9,898.91~18,757.08) | 9,897.48(7,049.7~13,358.21) | -11.97(-31.98~14.99) | -2.67(-3.1~-2.25) |
| Ethiopia | 1302,645.52(1143,586.97~1488,970.48) | 8,159.32(7,163.04~9,326.4) | -49.49(-53.67~-45.47) | -3.74(-3.88~-3.61) | 13,507.69(9,947.58~17,151.63) | 84.61(62.31~107.43) | -50.37(-62.3~-31.44) | -6.84(-7.19~-6.5) | 1200,808.1(885,714.43~1525,167.46) | 7,521.45(5,547.81~9,553.13) | -51.9(-63.52~-34.19) | -6.84(-7.18~-6.49) |
| Fiji | 3,529.75(3,022.06~4,100.02) | 3,876.92(3,319.3~4,503.28) | -50.96(-55.78~-46.05) | -2.46(-2.55~-2.36) | 36.49(25.28~50.52) | 40.08(27.76~55.49) | -33.55(-45.98~-13.53) | -2.23(-2.67~-1.79) | 3,254.74(2,257.9~4,503.63) | 3,574.85(2,479.97~4,946.58) | -34.63(-47.31~-15.75) | -2.24(-2.68~-1.8) |
| Finland | 1,611.27(1,284.59~1,989.91) | 663.62(529.08~819.57) | -52.88(-59.26~-46.81) | -2.48(-2.58~-2.38) | 1.84(1.54~2.17) | 0.76(0.63~0.89) | -43.39(-49.83~-35.64) | -5.28(-7.23~-3.28) | 166.06(139.37~195.6) | 68.39(57.4~80.56) | -56.54(-62.4~-49.77) | -5.25(-7.17~-3.29) |
| France | 17,310.95(13,881.54~21,484.72) | 489.85(392.81~607.96) | -55.67(-60.48~-51.1) | -2.26(-2.43~-2.1) | 30.59(25.58~36.16) | 0.87(0.72~1.02) | -53.96(-59.64~-47.37) | -4.98(-7.37~-2.52) | 2,752.66(2,298.65~3,260.11) | 77.89(65.05~92.25) | -60.24(-65.51~-54.15) | -4.96(-7.34~-2.53) |
| Gabon | 9,829.76(8,362.04~11,503.79) | 4,603.27(3,915.93~5,387.21) | -54.19(-60.16~-47.31) | -3.4(-3.45~-3.35) | 85.35(50.59~129.68) | 39.97(23.69~60.73) | -49.71(-60.97~-34.29) | -5.27(-5.58~-4.96) | 7,623.36(4,531.12~11,580.12) | 3,570.01(2,121.92~5,422.95) | -51.48(-62.52~-37.17) | -5.27(-5.58~-4.96) |
| Gambia | 17,874.03(15,584.37~20,841.9) | 4,998.48(4,358.17~5,828.44) | -55.81(-61.12~-49.4) | -3.85(-4.07~-3.63) | 250.04(187.76~330.63) | 69.92(52.51~92.46) | -44.65(-59.78~-26.58) | -5.63(-6.52~-4.73) | 22,234.78(16,715.79~29,437.69) | 6,217.96(4,674.58~8,232.26) | -46.69(-60.98~-29.63) | -5.62(-6.5~-4.73) |
| Georgia | 3,808.91(3,319.51~4,354) | 1,565.56(1,364.4~1,789.61) | -79.6(-82.15~-76.71) | -5.46(-5.63~-5.29) | 35.9(27.77~46.79) | 14.76(11.41~19.23) | -77.65(-80.14~-74.92) | -9.14(-10.36~-7.9) | 3,207.54(2,483.1~4,174.09) | 1,318.38(1,020.62~1,715.66) | -80.62(-82.98~-77.88) | -9.14(-10.36~-7.9) |
| Germany | 21,858.22(17,615.28~26,970.97) | 540.58(435.65~667.03) | -54.3(-58.99~-48.24) | -2.65(-2.78~-2.51) | 19.79(17.22~22.5) | 0.49(0.43~0.56) | -75.93(-79.23~-72.47) | -7.13(-8.45~-5.8) | 1,794.5(1,564.78~2,033.14) | 44.38(38.7~50.28) | -79.51(-82.36~-76.5) | -7.09(-8.39~-5.77) |
| Ghana | 262,422.61(229,254.34~302,941.37) | 5,655.5(4,940.68~6,528.72) | -29.97(-38.03~-20.99) | -3(-3.18~-2.81) | 2,698.66(1,706.87~3,879.21) | 58.16(36.78~83.6) | -32.56(-48.49~-13.4) | -4.65(-4.99~-4.32) | 240,232.33(152,377.1~345,400.66) | 5,177.27(3,283.89~7,443.76) | -34.4(-49.75~-16.09) | -4.65(-4.98~-4.31) |
| Greece | 2,439.48(1,996.7~2,940.96) | 577.24(472.47~695.9) | -52.15(-56.69~-46.92) | -2.3(-2.5~-2.1) | 5.61(4.52~6.84) | 1.33(1.07~1.62) | -15.77(-27.77~-3.59) | -3.19(-5.71~-0.61) | 504.8(407.72~614.63) | 119.45(96.48~145.44) | -28.59(-39.69~-17.14) | -3.19(-5.69~-0.62) |
| Greenland | 31.63(27.05~36.86) | 782.64(669.34~912.06) | -73.12(-76.37~-69.37) | -4.11(-4.47~-3.76) | 0.2(0.15~0.26) | 4.94(3.72~6.35) | -62.35(-72.78~-48.24) | -5.94(-6.46~-5.42) | 17.78(13.4~22.86) | 440.04(331.52~565.65) | -65.12(-74.85~-51.73) | -5.95(-6.47~-5.42) |
| Grenada | 203.39(175.14~239.44) | 2,961.75(2,550.41~3,486.59) | -63.17(-66.78~-59.22) | -3.31(-3.39~-3.23) | 1.19(0.9~1.5) | 17.3(13.16~21.82) | -44.83(-49.2~-39.84) | -3.51(-5.05~-1.96) | 106.48(81.18~134.18) | 1,550.52(1,182.05~1,953.95) | -48(-52.86~-42.71) | -3.51(-5.04~-1.96) |
| Guam | 483.09(417.56~558.46) | 3,780.38(3,267.53~4,370.18) | -43.91(-48.91~-38.1) | -1.84(-1.89~-1.79) | 3.54(2.61~4.57) | 27.69(20.41~35.74) | -22.85(-35.18~-9.26) | -1.28(-1.56~-1) | 316.44(233.58~407.94) | 2,476.28(1,827.83~3,192.28) | -23.71(-36.54~-10.3) | -1.29(-1.56~-1.01) |
| Guatemala | 71,715.85(61,453.7~83,023.43) | 4,599.33(3,941.19~5,324.52) | -64.68(-69.88~-58.85) | -3.73(-3.85~-3.62) | 1,717.35(1,333.64~2,206.15) | 110.14(85.53~141.49) | 14.31(6.3~23.17) | -3.64(-4.42~-2.86) | 153,414.72(119,184.26~197,028.76) | 9,838.9(7,643.61~12,635.99) | 9.7(1.28~18.72) | -3.63(-4.41~-2.85) |
| Guinea | 213,772.51(186,949.65~242,921.52) | 9,498.99(8,307.12~10,794.23) | -33.6(-41.34~-23.88) | -2.84(-2.97~-2.71) | 5,059.6(3,431.87~7,006.34) | 224.82(152.5~311.33) | -37.72(-54.52~-14.67) | -4.84(-5.47~-4.2) | 450,224.65(305,775.37~623,286.46) | 20,005.75(13,587.14~27,695.76) | -38.85(-55.26~-16.48) | -4.83(-5.46~-4.19) |
| Guinea-Bissau | 21,123.67(18,579~23,804.42) | 6,350.02(5,585.06~7,155.88) | -41.27(-48.66~-33.17) | -3.43(-3.57~-3.28) | 261.2(180.4~350.46) | 78.52(54.23~105.35) | -52.2(-65.14~-34.77) | -6.33(-7~-5.65) | 23,235.56(16,091.02~31,158.9) | 6,984.87(4,837.14~9,366.72) | -53.27(-65.81~-36.55) | -6.31(-6.98~-5.64) |
| Guyana | 1,662.39(1,433.96~1,933.67) | 2,231.03(1,924.45~2,595.1) | -60.73(-64.18~-56.83) | -3.31(-3.45~-3.17) | 23.61(17.28~32.01) | 31.68(23.19~42.96) | 0.78(-14.77~20.14) | -3.22(-4.73~-1.69) | 2,112.3(1,547.21~2,862.91) | 2,834.83(2,076.44~3,842.18) | -3.67(-19.65~15.33) | -3.22(-4.73~-1.7) |
| Haiti | 98,167.77(85,928.32~111,770.9) | 6,253.33(5,473.67~7,119.86) | -59.91(-64.03~-55.95) | -3.53(-3.58~-3.48) | 2,530.61(1,741.75~3,385.26) | 161.2(110.95~215.64) | -39.49(-54.93~-19.94) | -4.2(-4.42~-3.98) | 226,181.46(155,717.1~302,312.33) | 14,407.87(9,919.25~19,257.44) | -40.7(-55.85~-21.71) | -4.19(-4.41~-3.97) |
| Honduras | 39,596.89(32,855.48~47,908.41) | 3,614.43(2,999.07~4,373.11) | -68.53(-73.31~-62.17) | -4.2(-4.43~-3.96) | 276.96(187.55~379.68) | 25.28(17.12~34.66) | -43.48(-61.58~-22.71) | -6.11(-6.36~-5.86) | 24,803.92(16,832.88~33,987.42) | 2,264.12(1,536.52~3,102.4) | -47.35(-64.38~-28.53) | -6.1(-6.35~-5.86) |
| Hungary | 7,673.44(6,397.2~9,181.95) | 1,688.67(1,407.81~2,020.64) | -68.72(-71.88~-65.08) | -3.66(-3.78~-3.54) | 12.05(9.01~15.79) | 2.65(1.98~3.48) | -44.73(-54.97~-32.16) | -4.84(-5.53~-4.14) | 1,087.64(815.85~1,422.64) | 239.35(179.54~313.08) | -55.41(-63.87~-45.25) | -4.83(-5.52~-4.13) |
| Iceland | 167.78(126.94~212.89) | 763.61(577.72~968.91) | -41.98(-52.84~-31.29) | -1.69(-1.94~-1.44) | 0.3(0.23~0.39) | 1.39(1.05~1.78) | -28.38(-41.14~-13.09) | -4.37(-5.68~-3.05) | 27.31(20.74~34.98) | 124.28(94.38~159.2) | -42.93(-54.97~-30.08) | -4.37(-5.68~-3.04) |
| India | 10384,347.59(9034,194.55~12147,522.97) | 9,327.01(8,114.33~10,910.66) | -28.45(-35.94~-20.79) | -2.69(-2.94~-2.43) | 104,825.51(80,185.64~134,269.3) | 94.15(72.02~120.6) | -11.86(-28.09~10.85) | -4.43(-4.73~-4.12) | 9396,065.74(7191,969.34~12031,071.79) | 8,439.36(6,459.68~10,806.07) | -16.3(-31.49~4.54) | -4.42(-4.72~-4.11) |
| Indonesia | 907,341.21(793,680.93~1042,691.05) | 4,141.87(3,623.03~4,759.72) | -76.09(-78.23~-73.76) | -4.63(-5.32~-3.94) | 8,024.79(6,148.3~10,399.51) | 36.63(28.07~47.47) | -40.99(-52.17~-27.53) | -5.56(-5.8~-5.32) | 717,650.03(550,137.32~929,717.37) | 3,275.96(2,511.29~4,244.01) | -43.38(-54.12~-30.47) | -5.56(-5.8~-5.32) |
| Iran (Islamic Republic of) | 82,758.43(68,830.59~98,567.18) | 1,344.54(1,118.26~1,601.38) | -74.53(-78.19~-70.44) | -5.38(-5.46~-5.31) | 183.8(115.11~259.65) | 2.99(1.87~4.22) | -60.45(-80.1~-44.91) | -10.82(-11.56~-10.07) | 16,496.06(10,410.08~23,263.46) | 268.01(169.13~377.95) | -69.47(-84.75~-57.03) | -10.81(-11.55~-10.07) |
| Iraq | 120,381.09(100,531.68~145,716.64) | 2,803.84(2,341.52~3,393.94) | -66.47(-70.81~-61.13) | -4.29(-4.49~-4.1) | 1,007.22(746.43~1,364.08) | 23.46(17.39~31.77) | -44.17(-62~-25.01) | -5.75(-6.11~-5.38) | 89,866.25(66,596.24~121,778.88) | 2,093.11(1,551.12~2,836.4) | -47.34(-63.87~-28.81) | -5.76(-6.12~-5.39) |
| Ireland | 1,477.45(1,206.27~1,833.7) | 495.12(404.25~614.51) | -64.12(-69.21~-58.28) | -3.16(-3.28~-3.04) | 3.89(3.22~4.62) | 1.3(1.08~1.55) | -57.03(-63.57~-49.94) | -5.8(-7.52~-4.05) | 349.44(288.95~415.44) | 117.1(96.83~139.22) | -64.56(-70.04~-58.83) | -5.78(-7.5~-4.02) |
| Israel | 4,199.33(3,422.21~5,118.63) | 457.31(372.68~557.42) | -66.4(-70.96~-62.17) | -3.53(-3.62~-3.44) | 4.17(3.15~5.2) | 0.45(0.34~0.57) | -64.58(-71.66~-56.31) | -8.11(-10.08~-6.09) | 378.88(288.53~470.89) | 41.26(31.42~51.28) | -73.59(-79.22~-67.81) | -8.07(-10~-6.1) |
| Italy | 12,671.83(10,718.26~14,992.22) | 583.92(493.9~690.84) | -57.97(-61.82~-54.11) | -2.86(-2.99~-2.72) | 15.1(11.67~18.24) | 0.7(0.54~0.84) | -65.59(-70.43~-61.28) | -5.67(-6.97~-4.34) | 1,363.26(1,059.84~1,644.11) | 62.82(48.84~75.76) | -73.04(-77.47~-69.13) | -5.64(-6.94~-4.31) |
| Jamaica | 3,937.93(3,305.2~4,611.82) | 2,300.44(1,930.81~2,694.12) | -63.48(-68.62~-58.18) | -3.78(-3.84~-3.72) | 12.96(9.06~17.85) | 7.57(5.29~10.43) | -44.28(-51.58~-36.41) | -4.11(-5.7~-2.49) | 1,166.4(817.83~1,605.76) | 681.38(477.76~938.04) | -47.02(-54~-39.97) | -4.1(-5.68~-2.49) |
| Japan | 72,969.71(61,311.06~86,503.48) | 1,591.34(1,337.08~1,886.48) | -59.47(-62.59~-56.57) | -2.88(-2.97~-2.78) | 49.15(43.51~55.28) | 1.07(0.95~1.21) | -51.16(-54.65~-47.57) | -5.61(-7.26~-3.93) | 4,480.07(3,982.14~5,032.25) | 97.7(86.84~109.74) | -64.07(-67.77~-60.7) | -5.56(-7.19~-3.91) |
| Jordan | 22,235.28(18,476.52~26,674.2) | 2,026.33(1,683.79~2,430.86) | -65.15(-69.5~-59.76) | -3.73(-3.82~-3.65) | 164.62(128.68~215.62) | 15(11.73~19.65) | -45.28(-58.03~-31.94) | -5.59(-5.95~-5.22) | 14,736.52(11,528.84~19,288.05) | 1,342.96(1,050.64~1,757.75) | -49.56(-61.32~-36.6) | -5.59(-5.96~-5.22) |
| Kazakhstan | 41,590.51(36,500.88~47,620.52) | 2,134.55(1,873.34~2,444.03) | -70.77(-75.18~-65.77) | -4.69(-4.84~-4.54) | 387.3(310.96~477.07) | 19.88(15.96~24.48) | -73.61(-76.9~-70.03) | -7.47(-8.81~-6.11) | 34,711.4(27,879.43~42,718.63) | 1,781.5(1,430.86~2,192.45) | -75.83(-78.86~-72.57) | -7.46(-8.8~-6.11) |
| Kenya | 540,303.01(474,596.57~612,476.64) | 9,080.44(7,976.17~10,293.41) | -31.38(-36.72~-26.09) | -2.51(-3.21~-1.82) | 3,755.35(2,920.06~4,806.68) | 63.11(49.08~80.78) | -41.94(-54.44~-27.94) | -5.18(-5.61~-4.74) | 336,036.94(261,402.73~429,718.85) | 5,647.5(4,393.19~7,221.94) | -43.6(-55.82~-30.04) | -5.17(-5.61~-4.73) |
| Kiribati | 523.12(443.64~610.96) | 3,660.12(3,103.99~4,274.68) | -58.16(-62.67~-53.37) | -3.54(-3.69~-3.39) | 8.51(5.81~11.67) | 59.55(40.67~81.62) | -31.12(-51.32~-5.42) | -4.08(-4.49~-3.66) | 759.36(519.17~1,040.2) | 5,312.95(3,632.44~7,277.9) | -33.11(-52.57~-7.92) | -4.08(-4.49~-3.66) |
| Kuwait | 5,631.57(4,598.16~6,803.52) | 2,125.01(1,735.06~2,567.23) | -58.6(-64.34~-50.82) | -3.29(-3.37~-3.22) | 44.78(35.94~54.23) | 16.9(13.56~20.46) | -0.03(-15.08~14.32) | -0.57(-4.09~3.08) | 4,007.41(3,218.97~4,846.68) | 1,512.15(1,214.64~1,828.84) | -7.04(-20.89~8.31) | -0.57(-4.09~3.09) |
| Kyrgyzstan | 23,991.56(20,671.29~28,302.4) | 3,021.99(2,603.77~3,564.99) | -73.69(-77.71~-69.15) | -5.1(-5.32~-4.88) | 314.75(263.45~370.84) | 39.65(33.18~46.71) | -71.7(-75.67~-67.39) | -7.67(-9~-6.33) | 28,065.22(23,482.78~33,077.55) | 3,535.11(2,957.9~4,166.47) | -73.74(-77.66~-69.86) | -7.68(-9.01~-6.33) |
| Lao People's Democratic Republic | 36,885.97(31,727.23~42,361.74) | 4,446.59(3,824.71~5,106.7) | -73.06(-76.06~-69.74) | -5.27(-5.55~-4.99) | 1,060.63(750.91~1,446.13) | 127.86(90.52~174.33) | -42.02(-56.31~-25.02) | -6.15(-6.35~-5.95) | 94,864.49(67,183.96~129,251.61) | 11,435.89(8,099.01~15,581.25) | -43.66(-57.27~-27.06) | -6.14(-6.34~-5.94) |
| Latvia | 1,728.8(1,397.63~2,108.52) | 1,844.77(1,491.38~2,249.96) | -65.86(-70.92~-60.22) | -3.61(-4.15~-3.08) | 4.16(3.44~4.97) | 4.44(3.67~5.3) | -18.05(-26.53~-9.2) | -5.17(-6.51~-3.82) | 374.87(310.69~446.95) | 400.01(331.53~476.93) | -34.6(-43.51~-25.09) | -5.16(-6.49~-3.81) |
| Lebanon | 7,349.34(5,871.34~8,932.81) | 1,810.63(1,446.5~2,200.74) | -59.65(-65.16~-52.82) | -3.14(-3.22~-3.06) | 59.64(40.57~87.07) | 14.69(10~21.45) | -32.9(-53~-7.46) | -6.07(-6.42~-5.72) | 5,342.49(3,635.92~7,797.16) | 1,316.21(895.77~1,920.96) | -41.52(-58.6~-17.7) | -6.07(-6.42~-5.72) |
| Lesotho | 15,162.39(13,142.42~17,537.24) | 7,442.96(6,451.39~8,608.73) | -23.07(-31.77~-13) | -1.86(-2.4~-1.31) | 404.91(285.26~537.67) | 198.76(140.03~263.93) | -5.28(-24.86~22.21) | -0.88(-1.36~-0.41) | 36,219.53(25,525.92~48,056.53) | 17,779.55(12,530.24~23,590.13) | -5.32(-24.99~21.96) | -0.88(-1.35~-0.41) |
| Liberia | 40,354.45(35,072.73~46,154.28) | 5,265.54(4,576.37~6,022.31) | -64.76(-69.61~-58.88) | -4.49(-4.66~-4.32) | 568.54(353.03~840.09) | 74.18(46.06~109.62) | -63.2(-72.33~-51.66) | -7.62(-8.5~-6.74) | 50,672.96(31,510.34~74,772.5) | 6,611.92(4,111.54~9,756.48) | -64.11(-73.01~-53.09) | -7.61(-8.5~-6.72) |
| Libya | 8,753.05(7,202.86~10,657.48) | 2,068.91(1,702.5~2,519.05) | -67.62(-72.26~-62.87) | -4.09(-4.17~-4.02) | 63.49(43.33~91.03) | 15.01(10.24~21.52) | -47.91(-62.13~-24.77) | -4.05(-5.44~-2.63) | 5,639.4(3,854.83~8,074.29) | 1,332.95(911.15~1,908.48) | -49.87(-63.43~-27.13) | -4.07(-5.46~-2.65) |
| Lithuania | 3,375.84(2,642.82~4,287.65) | 2,565.34(2,008.31~3,258.24) | -53.92(-61.89~-42.18) | -2.59(-2.92~-2.26) | 5.58(4.6~6.88) | 4.24(3.5~5.23) | -4.26(-20.82~17.01) | -4.96(-6.28~-3.61) | 503.86(415.6~619.5) | 382.89(315.82~470.77) | -25.36(-39.02~-8.03) | -4.94(-6.26~-3.6) |
| Luxembourg | 162.18(131.65~198.51) | 490.51(398.16~600.36) | -60.72(-65.27~-55.63) | -3.02(-3.17~-2.87) | 0.23(0.19~0.29) | 0.7(0.57~0.87) | -59.78(-64.92~-54.63) | -6.04(-8~-4.05) | 21.01(16.97~25.91) | 63.55(51.33~78.37) | -65.83(-70.49~-60.81) | -6.02(-7.94~-4.05) |
| Madagascar | 274,967.96(233,596.62~320,833.21) | 6,726.13(5,714.12~7,848.06) | -49.61(-56.1~-42.38) | -3.7(-4.03~-3.37) | 5,323.13(3,785.57~7,048.22) | 130.21(92.6~172.41) | -32.4(-47.28~-14.57) | -4.56(-4.84~-4.27) | 476,057.8(338,813.98~630,187.97) | 11,645.08(8,287.89~15,415.33) | -33.66(-48.02~-16.67) | -4.55(-4.83~-4.27) |
| Malawi | 195,209.87(169,156.69~224,761.16) | 7,166.15(6,209.74~8,250.98) | -45.56(-51.96~-37.57) | -3.32(-3.68~-2.95) | 2,899.61(1,943.63~3,885.95) | 106.44(71.35~142.65) | -22.41(-41.85~2.7) | -5.79(-6.32~-5.25) | 258,362.15(173,611.33~346,054.61) | 9,484.47(6,373.27~12,703.66) | -25(-43.73~-0.74) | -5.78(-6.31~-5.24) |
| Malaysia | 63,899.37(52,563.1~76,753.01) | 2,599.21(2,138.09~3,122.05) | -70.79(-75.25~-65.93) | -3.93(-4.11~-3.74) | 220.86(167.97~290.4) | 8.98(6.83~11.81) | -40.28(-56.59~-16.39) | -5.05(-6.69~-3.38) | 19,824.23(15,120.67~26,058.8) | 806.38(615.06~1,059.98) | -47.7(-62.08~-26.19) | -5.04(-6.68~-3.38) |
| Maldives | 619.52(523.92~724.79) | 1,950.75(1,649.72~2,282.21) | -71.18(-75.1~-67.09) | -4.6(-4.7~-4.5) | 4.07(3.04~5.33) | 12.82(9.58~16.79) | -49.33(-61.3~-34.37) | -7.59(-8.23~-6.94) | 363.72(272.1~476.15) | 1,145.29(856.8~1,499.29) | -54.04(-64.63~-39.68) | -7.57(-8.2~-6.94) |
| Mali | 321,970.47(280,781.84~367,794.5) | 7,029.34(6,130.1~8,029.78) | -6.4(-18.19~7.96) | -2.05(-2.15~-1.96) | 6,104.63(4,415.23~8,171.01) | 133.28(96.39~178.39) | -11.31(-33.52~15.56) | -3.44(-3.82~-3.07) | 543,004.01(393,698.16~726,829.16) | 11,855(8,595.32~15,868.31) | -12.87(-34.26~12.71) | -3.44(-3.81~-3.07) |
| Malta | 151.66(125.85~182.2) | 689.28(572~828.07) | -54.83(-59.31~-49.52) | -2.56(-2.62~-2.49) | 0.38(0.29~0.49) | 1.73(1.31~2.21) | -48.01(-54.75~-40.49) | -4.2(-5.56~-2.83) | 34.3(26.07~43.77) | 155.9(118.5~198.91) | -52.83(-59.86~-45.34) | -4.19(-5.53~-2.84) |
| Marshall Islands | 281.37(243.99~327.69) | 4,959.42(4,300.59~5,775.91) | -48.69(-54.59~-42.57) | -2.68(-2.82~-2.54) | 4.42(2.92~5.95) | 77.98(51.49~104.9) | -9.1(-29.53~14.7) | -1.92(-2.2~-1.64) | 394.97(261.01~531.03) | 6,961.84(4,600.62~9,359.88) | -11.95(-31.78~11.32) | -1.92(-2.2~-1.64) |
| Mauritania | 41,027.84(35,753.62~46,823.52) | 6,244.71(5,441.94~7,126.85) | -31.34(-38.44~-23.52) | -2.72(-3~-2.45) | 452.03(345.85~581.84) | 68.8(52.64~88.56) | -27.82(-45.54~-3.95) | -4.66(-5.07~-4.26) | 40,313.02(30,871.35~51,785.47) | 6,135.91(4,698.82~7,882.09) | -30.55(-47.44~-8.2) | -4.65(-5.05~-4.25) |
| Mauritius | 1,864.68(1,545.97~2,226.07) | 2,900.18(2,404.47~3,462.26) | -56.51(-62.76~-48.88) | -3.39(-3.46~-3.33) | 12.39(10.01~14.41) | 19.26(15.58~22.41) | -18.43(-27.46~-9.23) | -2.66(-4.8~-0.48) | 1,110.6(899.39~1,291.51) | 1,727.33(1,398.83~2,008.72) | -21.41(-30.02~-12.52) | -2.66(-4.8~-0.47) |
| Mexico | 164,074.01(140,288.17~191,710.84) | 1,661.15(1,420.33~1,940.96) | -71.98(-74.82~-69.08) | -4.62(-4.82~-4.42) | 1,801.9(1,298.38~2,443.46) | 18.24(13.15~24.74) | -62.76(-67.69~-57.89) | -6.79(-7.51~-6.06) | 161,259.64(116,311.25~218,511.81) | 1,632.66(1,177.58~2,212.3) | -64.98(-70.13~-59.82) | -6.78(-7.5~-6.05) |
| Micronesia (Federated States of) | 386.87(331.06~450.77) | 4,085.1(3,495.81~4,759.88) | -63.95(-67.54~-60.24) | -3.82(-4~-3.63) | 4.74(3.28~6.24) | 50.01(34.63~65.91) | -34.94(-50.71~-19.52) | -4.55(-4.9~-4.2) | 423.17(293.44~557.48) | 4,468.43(3,098.61~5,886.69) | -39.02(-53.1~-25.1) | -4.55(-4.9~-4.2) |
| Monaco | 8.31(6.67~10.22) | 513.6(411.91~631.68) | -62.22(-65.79~-57.79) | -3.04(-3.19~-2.89) | 0.04(0.03~0.05) | 2.33(1.79~3.03) | -16.95(-43.53~13.46) | -2.5(-3.46~-1.54) | 3.35(2.57~4.36) | 207.19(158.89~269.42) | -27.88(-51.2~-0.58) | -2.52(-3.46~-1.57) |
| Mongolia | 9,129.74(8,004.81~10,507.41) | 2,336.79(2,048.86~2,689.41) | -79.29(-81.95~-76.48) | -5.46(-5.74~-5.18) | 204.05(143.38~277.69) | 52.23(36.7~71.07) | -65.26(-74.69~-54.03) | -8.45(-9.06~-7.83) | 18,231.07(12,811.56~24,820.26) | 4,666.31(3,279.17~6,352.84) | -68.28(-76.8~-58.13) | -8.43(-9.04~-7.82) |
| Montenegro | 708.57(587.04~839.03) | 1,957.84(1,622.04~2,318.31) | -68.98(-72.43~-65.91) | -3.49(-3.68~-3.29) | 0.65(0.41~0.92) | 1.79(1.13~2.54) | -69.71(-84.15~-52.74) | -7.73(-8.85~-6.6) | 59.05(37.75~83.52) | 163.17(104.31~230.78) | -75.75(-87.13~-62.34) | -7.69(-8.8~-6.57) |
| Morocco | 89,767.37(74,536.55~107,507.91) | 2,761.4(2,292.87~3,307.13) | -65.65(-70.91~-59.01) | -4.58(-4.65~-4.51) | 669.84(412.57~947.9) | 20.61(12.69~29.16) | -56.3(-72.9~-38.44) | -8.02(-8.23~-7.81) | 60,078.92(37,061.74~84,963.79) | 1,848.13(1,140.08~2,613.63) | -59.96(-75~-44.06) | -8.01(-8.22~-7.81) |
| Mozambique | 268,784.5(232,939.04~309,736.24) | 5,189.67(4,497.57~5,980.37) | -53.36(-58.58~-47.47) | -3.88(-4.03~-3.72) | 4,227.94(3,004.42~5,842.23) | 81.63(58.01~112.8) | -39.06(-55.1~-14.01) | -5.99(-6.65~-5.32) | 377,693.21(268,582.96~521,895.99) | 7,292.48(5,185.78~10,076.74) | -40.66(-56.15~-17.02) | -5.98(-6.63~-5.31) |
| Myanmar | 239,334.01(207,537.43~275,249.1) | 4,579.32(3,970.94~5,266.5) | -65.98(-70.4~-61.16) | -4.65(-4.78~-4.52) | 6,256.03(4,538.01~8,161.03) | 119.7(86.83~156.15) | -39.99(-53.59~-23.27) | -5.96(-6.11~-5.81) | 559,365.97(405,804.12~729,719.31) | 10,702.68(7,764.49~13,962.15) | -41.84(-54.86~-25.54) | -5.95(-6.1~-5.8) |
| Namibia | 16,444.1(14,161.62~19,159.52) | 5,905.55(5,085.84~6,880.73) | -41.05(-49.25~-30.98) | -3.16(-3.58~-2.74) | 165.83(109.04~237.5) | 59.55(39.16~85.29) | -24.45(-44.07~1.83) | -3.58(-3.81~-3.36) | 14,784.76(9,729.67~21,174.68) | 5,309.63(3,494.2~7,604.43) | -26.16(-45.67~-0.45) | -3.58(-3.8~-3.35) |
| Nauru | 75.19(64.87~85.86) | 5,378.65(4,640.31~6,142.3) | -55.85(-60.38~-51.18) | -2.8(-2.9~-2.69) | 1.68(1.16~2.29) | 119.84(83.2~164.08) | -9.16(-23.81~10.54) | -1.5(-1.75~-1.26) | 149.15(103.51~204.3) | 10,669.68(7,404.56~14,615.15) | -10.74(-24.93~7.85) | -1.51(-1.75~-1.27) |
| Nepal | 329,892.69(285,504.3~380,226.99) | 10,619.81(9,190.87~12,240.15) | -61.74(-66.36~-56.07) | -4.5(-4.85~-4.14) | 2,346.94(1,712.3~3,262.3) | 75.55(55.12~105.02) | -47.85(-61.27~-33.28) | -7.27(-7.58~-6.95) | 210,365.87(153,746.51~292,128.9) | 6,772.03(4,949.36~9,404.12) | -50.3(-63.03~-36.34) | -7.25(-7.56~-6.94) |
| Netherlands | 3,556.95(2,885.06~4,341.13) | 413.01(334.99~504.06) | -61.13(-65.35~-56.77) | -2.51(-2.72~-2.29) | 9.75(8.34~11.14) | 1.13(0.97~1.29) | -51.82(-58.07~-44.49) | -4.85(-6.79~-2.88) | 876.08(749.57~1,000.29) | 101.72(87.03~116.15) | -58.55(-63.92~-52.6) | -4.84(-6.76~-2.87) |
| New Zealand | 2,235.72(1,906.7~2,632.31) | 715.2(609.95~842.07) | -67.7(-70.95~-64.11) | -3.89(-4.5~-3.28) | 6.5(5.49~7.49) | 2.08(1.76~2.4) | -65.52(-70.43~-60.57) | -6.43(-8.66~-4.15) | 585.49(495.1~674.44) | 187.3(158.38~215.75) | -69.94(-73.79~-65.72) | -6.42(-8.64~-4.14) |
| Nicaragua | 20,622.44(17,415.44~24,342.29) | 3,170.08(2,677.1~3,741.89) | -65.09(-69.71~-59.85) | -4.59(-5.16~-4.02) | 168.13(116.91~230.79) | 25.85(17.97~35.48) | -38.12(-50.86~-24.46) | -6.71(-7.11~-6.3) | 15,077.73(10,490.83~20,679.64) | 2,317.75(1,612.65~3,178.87) | -42.95(-54.71~-29.29) | -6.69(-7.1~-6.29) |
| Niger | 476,150.48(417,543.32~544,449.54) | 9,342.99(8,193~10,683.15) | -41(-49.07~-32.96) | -2.92(-3.09~-2.75) | 11,071.36(7,702.49~15,292.59) | 217.24(151.14~300.07) | -41.97(-55.06~-23.92) | -5.81(-6.35~-5.27) | 982,215.93(684,773.01~1355,217.78) | 19,272.96(13,436.56~26,591.98) | -43.11(-55.94~-25.54) | -5.8(-6.34~-5.26) |
| Nigeria | 3313,127.32(2956,782.51~3699,431.14) | 8,925.97(7,965.93~9,966.72) | -43.8(-47.64~-39.4) | -2.92(-3.05~-2.78) | 100,329.92(68,754.26~136,663.79) | 270.3(185.23~368.19) | -28.77(-45.39~-11.03) | -3.79(-4.08~-3.51) | 8908,162.4(6114,236.4~12126,351.31) | 23,999.68(16,472.5~32,669.87) | -29.74(-46.05~-12.27) | -3.8(-4.08~-3.52) |
| Niue | 4.86(4.17~5.64) | 4,136.54(3,550.08~4,802.79) | -59.69(-63.51~-55.67) | -3.07(-3.12~-3.03) | 0.27(0.23~0.3) | 225.95(193.77~257.54) | -4.06(-19.13~15.31) | 2.98(1.25~4.75) | 23.59(20.22~26.89) | 20,089.36(17,217.24~22,897.64) | 0.52(-15.94~21.14) | 2.97(1.24~4.73) |
| North Macedonia | 1,857.36(1,575.46~2,183.26) | 1,847.79(1,567.34~2,172.01) | -71.14(-74.59~-67.54) | -4.22(-4.52~-3.92) | 3.36(2.17~4.55) | 3.34(2.16~4.52) | -74.68(-85.93~-62.76) | -10.16(-11.32~-8.97) | 303.29(196.54~409.11) | 301.73(195.53~407) | -79.43(-88.43~-70.32) | -10.13(-11.3~-8.96) |
| Northern Mariana Islands | 152.3(127.51~178.17) | 4,748.87(3,975.91~5,555.7) | -47.25(-52.44~-41.09) | -1.77(-1.87~-1.67) | 0.37(0.28~0.48) | 11.62(8.68~15.09) | -31.61(-45.92~-14.55) | -3.23(-4.65~-1.8) | 33.44(25.03~43.43) | 1,042.77(780.43~1,354.28) | -38.05(-51.68~-21.88) | -3.23(-4.64~-1.8) |
| Norway | 1,631.57(1,347.91~1,991.21) | 580.39(479.48~708.32) | -54.15(-57.48~-50.98) | -2.54(-2.82~-2.26) | 1.24(1.04~1.44) | 0.44(0.37~0.51) | -57.25(-60.95~-53.27) | -7.61(-10.15~-4.99) | 112.95(95.59~131.01) | 40.18(34~46.6) | -68.86(-72.34~-64.92) | -7.54(-10.03~-4.98) |
| Oman | 8,132.28(6,620.16~9,846.31) | 1,917.89(1,561.28~2,322.12) | -72.61(-76.2~-67.8) | -4.91(-5.21~-4.61) | 48.75(38.16~61.12) | 11.5(9~14.41) | -42.38(-64.16~-19.86) | -6.49(-6.86~-6.12) | 4,366.59(3,418.3~5,467.57) | 1,029.8(806.16~1,289.46) | -48.47(-68.27~-28.58) | -6.48(-6.86~-6.11) |
| Pakistan | 3622,388.37(3151,882.04~4125,773.14) | 12,185.93(10,603.12~13,879.34) | -49.52(-54.81~-44.24) | -3.05(-3.43~-2.67) | 37,580.37(28,200.28~48,633.4) | 126.42(94.87~163.61) | -28.44(-42.07~-10.05) | -3.51(-3.96~-3.06) | 3356,981.24(2521,010.31~4344,117.66) | 11,293.08(8,480.83~14,613.87) | -30.15(-43.69~-12.25) | -3.52(-3.97~-3.07) |
| Palau | 37.32(32.08~43.99) | 3,940.27(3,387.13~4,644.9) | -65.23(-68.89~-61.09) | -3.72(-3.8~-3.64) | 0.46(0.36~0.59) | 48.98(37.76~62.43) | -24.14(-37.94~-4.47) | -3.18(-3.53~-2.83) | 41.43(31.95~52.78) | 4,374.37(3,373.33~5,573.55) | -27.34(-40.72~-9.15) | -3.19(-3.54~-2.84) |
| Palestine | 13,293.77(10,969.66~15,950.31) | 2,169.5(1,790.21~2,603.04) | -74.76(-78.1~-70.37) | -4.8(-5.14~-4.46) | 81.85(59.01~115.13) | 13.36(9.63~18.79) | -41.6(-61.16~-18.64) | -6.24(-6.48~-6) | 7,309.93(5,275.82~10,283.45) | 1,192.96(861~1,678.23) | -46.59(-64.33~-24.1) | -6.24(-6.48~-6) |
| Panama | 11,748.94(9,408.33~14,955.38) | 3,164.84(2,534.35~4,028.57) | -40.98(-50.3~-27.83) | -2.4(-3~-1.8) | 91.71(69.86~115.37) | 24.7(18.82~31.08) | -6.51(-17.71~9.85) | -2.71(-3.78~-1.63) | 8,206.1(6,259.64~10,313.3) | 2,210.5(1,686.18~2,778.12) | -10.32(-21.21~5.26) | -2.7(-3.77~-1.62) |
| Papua New Guinea | 133,492.51(116,601.45~153,341.72) | 8,774.83(7,664.53~10,079.57) | -48.38(-54.9~-37.44) | -2.6(-2.7~-2.5) | 4,221.28(3,256.22~5,466.87) | 277.48(214.04~359.35) | -24.51(-38.27~-8.14) | -2.4(-2.69~-2.11) | 377,459.61(291,207.55~488,946.95) | 24,811.45(19,141.87~32,139.82) | -25.41(-38.76~-9.02) | -2.4(-2.69~-2.12) |
| Paraguay | 14,840.51(12,457.32~17,687.25) | 2,282.91(1,916.31~2,720.83) | -71.98(-75.88~-67.34) | -5.32(-5.8~-4.84) | 83.02(50.13~121.91) | 12.77(7.71~18.75) | -63.41(-74.42~-50.79) | -6.16(-8.06~-4.23) | 7,404.69(4,479.85~10,856.28) | 1,139.06(689.14~1,670.02) | -65.33(-76.39~-53.41) | -6.17(-8.07~-4.24) |
| Peru | 142,383.7(109,800.56~175,819.93) | 4,314.01(3,326.79~5,327.08) | -68.09(-74.74~-61.34) | -5.32(-5.9~-4.75) | 1,064.8(685.44~1,536.12) | 32.26(20.77~46.54) | -50.35(-64.19~-33.09) | -8.05(-9.69~-6.38) | 95,229.87(61,395.25~137,204.16) | 2,885.32(1,860.18~4,157.08) | -54.51(-67.35~-39.14) | -8.05(-9.68~-6.38) |
| Philippines | 487,277.14(425,772.93~555,102.55) | 4,345.5(3,797.01~4,950.36) | -62.13(-64.31~-59.8) | -4.43(-4.68~-4.19) | 5,722.27(4,500.23~7,260.74) | 51.03(40.13~64.75) | -45.5(-57.29~-35.43) | -5.18(-5.61~-4.75) | 510,048.91(401,517.4~646,737.88) | 4,548.57(3,580.7~5,767.55) | -48.11(-59.69~-38.62) | -5.17(-5.6~-4.74) |
| Poland | 49,000.5(42,941.87~55,682.86) | 2,603.53(2,281.62~2,958.59) | -24.58(-30.54~-18.7) | -1.04(-1.22~-0.86) | 55.53(43.07~68.38) | 2.95(2.29~3.63) | -34.22(-44.12~-23.37) | -5.78(-9.18~-2.25) | 5,030.17(3,929.32~6,175.94) | 267.27(208.78~328.14) | -47.37(-56.85~-37.46) | -5.75(-9.09~-2.28) |
| Portugal | 2,288.07(1,895.22~2,755.71) | 537.96(445.59~647.9) | -74.06(-78.27~-69.61) | -4.34(-4.56~-4.11) | 5.47(4.4~6.57) | 1.29(1.04~1.55) | -61.01(-67.43~-54.04) | -7.94(-11.33~-4.41) | 490.86(395.25~589.34) | 115.41(92.93~138.56) | -70.33(-75.4~-64.23) | -7.92(-11.31~-4.4) |
| Puerto Rico | 2,078.03(1,689.5~2,525.08) | 1,976.78(1,607.19~2,402.05) | -65.08(-69.39~-60.31) | -3.41(-3.56~-3.26) | 4.24(3.45~5.2) | 4.04(3.28~4.95) | -35.73(-43.55~-27) | -4.31(-5.17~-3.45) | 383.15(312.32~468.86) | 364.48(297.1~446.02) | -45.63(-52.92~-38.36) | -4.3(-5.15~-3.44) |
| Qatar | 2,719.56(2,288.01~3,233.3) | 1,475.61(1,241.45~1,754.36) | -68.86(-72.31~-65.1) | -3.98(-4.03~-3.93) | 6.63(4.74~9.18) | 3.6(2.57~4.98) | -40.19(-59.89~-14.06) | -6.59(-6.97~-6.2) | 596.53(427.42~825.48) | 323.67(231.92~447.9) | -52.47(-68.25~-32.94) | -6.58(-6.96~-6.19) |
| Republic of Korea | 22,944.18(18,724.49~27,874.53) | 1,480.37(1,208.11~1,798.48) | -73.99(-77.83~-69.74) | -4.37(-4.47~-4.27) | 10.13(7.36~13.37) | 0.65(0.48~0.86) | -77.47(-82.7~-71.23) | -9.67(-10.14~-9.2) | 937.12(694.98~1,225.64) | 60.46(44.84~79.08) | -83.88(-87.91~-79.05) | -9.6(-10.06~-9.14) |
| Republic of Moldova | 5,065.68(4,237.53~6,049.43) | 3,284.65(2,747.66~3,922.52) | -66.3(-70.27~-61.81) | -3.69(-3.78~-3.59) | 35.86(26.85~47.6) | 23.25(17.41~30.87) | -47.23(-51.85~-42.54) | -5.01(-6.24~-3.77) | 3,217.41(2,412.14~4,270.2) | 2,086.21(1,564.06~2,768.85) | -51.14(-55.9~-46.28) | -5.01(-6.23~-3.76) |
| Romania | 22,737.06(19,981.14~26,033.66) | 2,424.38(2,130.53~2,775.89) | -75.27(-79.07~-71.13) | -4.28(-4.51~-4.04) | 200.24(170.64~230.53) | 21.35(18.19~24.58) | -54.24(-60.06~-48.45) | -6.16(-7.03~-5.29) | 17,944.27(15,294.59~20,648.41) | 1,913.34(1,630.81~2,201.67) | -60.64(-65.8~-55.62) | -6.16(-7.03~-5.28) |
| Russian Federation | 107,409.18(88,982.35~127,265.68) | 1,411.3(1,169.18~1,672.2) | -78.46(-81.27~-75.3) | -5.26(-5.49~-5.03) | 518.34(459.79~572.73) | 6.81(6.04~7.53) | -57.05(-61.46~-53.8) | -6.4(-7.7~-5.09) | 46,496.16(41,236.93~51,366.65) | 610.94(541.83~674.93) | -63.19(-67.28~-59.77) | -6.4(-7.69~-5.09) |
| Rwanda | 107,709.85(91,601.98~125,783.69) | 6,160.26(5,239~7,193.96) | -65.3(-70.47~-59.4) | -4.56(-4.87~-4.25) | 1,386.73(1,017.1~1,800.4) | 79.31(58.17~102.97) | -50.16(-64.8~-27.54) | -6.73(-7.89~-5.57) | 123,278.47(90,550.75~159,953.52) | 7,050.68(5,178.88~9,148.24) | -51.64(-65.78~-29.66) | -6.73(-7.88~-5.56) |
| Saint Kitts and Nevis | 70.98(60.4~82.27) | 2,326.14(1,979.6~2,696.01) | -65.98(-70.02~-62.02) | -3.66(-3.75~-3.56) | 0.65(0.49~0.84) | 21.22(15.91~27.49) | -9.09(-18.11~0.71) | -2.52(-3.58~-1.45) | 57.96(43.5~75.04) | 1,899.56(1,425.64~2,459.1) | -13.49(-23.12~-4.02) | -2.52(-3.58~-1.45) |
| Saint Lucia | 209.62(178.28~245.84) | 2,373.03(2,018.31~2,783.13) | -64.13(-67.63~-60.46) | -3.38(-3.57~-3.2) | 1.13(0.78~1.58) | 12.84(8.84~17.85) | -36.57(-42.66~-29.61) | -2.99(-4.18~-1.78) | 101.79(70.22~141.27) | 1,152.35(794.89~1,599.25) | -38.61(-45.46~-31.34) | -2.98(-4.17~-1.77) |
| Saint Vincent and the Grenadines | 176.57(149.71~207.17) | 2,453.64(2,080.43~2,878.85) | -61.25(-65.13~-56.8) | -3.31(-3.42~-3.19) | 0.92(0.68~1.26) | 12.77(9.49~17.45) | -16.23(-24.1~-6.7) | -3.47(-4.44~-2.49) | 82.42(61.35~112.6) | 1,145.25(852.58~1,564.63) | -23.15(-31.24~-12.8) | -3.47(-4.43~-2.49) |
| Samoa | 1,138.29(989.14~1,314.85) | 3,892.38(3,382.36~4,496.14) | -62.88(-66.14~-59.27) | -3.53(-3.65~-3.41) | 13.25(8.43~19.26) | 45.31(28.84~65.87) | -24.16(-46.49~5.34) | -3.51(-3.92~-3.11) | 1,183.16(754.44~1,718.44) | 4,045.81(2,579.82~5,876.2) | -28.47(-49.28~-1.09) | -3.52(-3.91~-3.12) |
| San Marino | 6.13(5.04~7.54) | 507.27(416.5~623.21) | -61.11(-65.02~-56.99) | -2.94(-3.05~-2.83) | 0.01(0~0.01) | 0.42(0.23~0.65) | -59.25(-75.03~-38.64) | -7.82(-8.12~-7.53) | 0.46(0.26~0.71) | 38.16(21.43~58.75) | -73.65(-84.52~-60.43) | -7.79(-8.15~-7.43) |
| Sao Tome and Principe | 1,188.93(1,023.41~1,376.74) | 4,766.38(4,102.8~5,519.31) | -53.34(-60.5~-42.1) | -4.23(-4.5~-3.97) | 7.73(5.09~11.12) | 30.99(20.41~44.58) | -49.89(-64.85~-33.1) | -7.71(-8.78~-6.62) | 692.02(456.33~995.59) | 2,774.29(1,829.42~3,991.29) | -54.07(-67.88~-38.75) | -7.68(-8.75~-6.6) |
| Saudi Arabia | 36,411.68(28,817.61~45,460.08) | 1,496.51(1,184.39~1,868.39) | -67.87(-72.44~-62.12) | -4.37(-4.42~-4.31) | 77.68(48.7~109.35) | 3.19(2~4.49) | -56.54(-77.38~-37.62) | -10.2(-10.49~-9.9) | 6,973.62(4,378.22~9,785.28) | 286.61(179.94~402.17) | -66.56(-82.17~-50.07) | -10.18(-10.47~-9.88) |
| Senegal | 134,477.87(115,805.09~157,981.83) | 5,920.36(5,098.3~6,955.12) | -32.27(-40.96~-21.96) | -3.06(-3.39~-2.72) | 1,813.99(1,335.45~2,422.35) | 79.86(58.79~106.64) | -30.71(-48.17~-7.82) | -5.2(-6.17~-4.22) | 162,082.53(119,373.6~216,560.99) | 7,135.65(5,255.4~9,534.05) | -33(-49.67~-10.49) | -5.17(-6.14~-4.2) |
| Serbia | 4,546.46(3,749.33~5,548.89) | 1,233.24(1,017.02~1,505.16) | -75(-78.55~-71.22) | -4.41(-4.65~-4.17) | 9.59(6.83~12.79) | 2.6(1.85~3.47) | -61.75(-77.59~-44.7) | -7.93(-10.42~-5.36) | 863.44(616.7~1,149.32) | 234.21(167.28~311.76) | -69.34(-81.89~-55.29) | -7.91(-10.4~-5.34) |
| Seychelles | 274.8(233.55~322.25) | 3,489.71(2,965.88~4,092.28) | -55.3(-59.92~-49.66) | -2.76(-2.84~-2.67) | 2.84(2.23~3.57) | 36.05(28.37~45.32) | -8.59(-22.44~11.05) | -1.22(-1.44~-1.01) | 254.31(200.16~319.64) | 3,229.52(2,541.84~4,059.17) | -10.62(-24.4~8.08) | -1.22(-1.43~-1) |
| Sierra Leone | 117,384.18(102,161.5~133,707.78) | 8,740.76(7,607.24~9,956.26) | -32.75(-40.9~-22.35) | -2.8(-2.97~-2.63) | 1,744.91(1,198.07~2,524.8) | 129.93(89.21~188) | -59.47(-68.72~-46.55) | -6.07(-6.88~-5.24) | 155,369.14(106,686.19~224,977.79) | 11,569.23(7,944.16~16,752.49) | -60.13(-69.44~-47.42) | -6.06(-6.87~-5.24) |
| Singapore | 6,359.12(5,417.24~7,544) | 2,224.02(1,894.61~2,638.41) | -66.71(-71.36~-61.99) | -3.46(-3.8~-3.11) | 6.53(5.31~7.79) | 2.29(1.86~2.72) | -48.17(-54.37~-41.26) | -7.84(-10.26~-5.37) | 592.01(482.63~703.66) | 207.05(168.79~246.1) | -67.37(-72.68~-62.1) | -7.81(-10.18~-5.37) |
| Slovakia | 4,051.83(3,467.01~4,743.89) | 1,416.07(1,211.69~1,657.94) | -75.59(-78.24~-72.64) | -4.43(-4.54~-4.33) | 34(25.9~42) | 11.88(9.05~14.68) | -48.53(-59.36~-36.53) | -4.97(-6.59~-3.32) | 3,044.17(2,321.36~3,760.52) | 1,063.91(811.29~1,314.26) | -53.94(-64.22~-42.47) | -4.96(-6.59~-3.32) |
| Slovenia | 1,333.07(1,072.34~1,645.16) | 1,361.68(1,095.36~1,680.47) | -65.96(-69.77~-62.05) | -3.17(-3.28~-3.05) | 4.4(3.65~5.18) | 4.49(3.73~5.29) | -23.13(-33.12~-12.59) | -5.17(-6.31~-4.02) | 394.94(327.56~464.56) | 403.42(334.59~474.53) | -45.47(-53.66~-35.86) | -5.16(-6.3~-4.02) |
| Solomon Islands | 6,224.2(5,437.19~7,200.45) | 6,528.55(5,703.06~7,552.54) | -52.77(-59.72~-38.18) | -3.13(-3.21~-3.06) | 103.95(77.79~134.39) | 109.03(81.59~140.96) | -12.17(-28.06~7.16) | -3.26(-4.01~-2.51) | 9,300.2(6,965.94~12,018.72) | 9,754.97(7,306.57~12,606.42) | -16.63(-31.87~1.31) | -3.26(-4~-2.52) |
| Somalia | 440,072.45(378,077.72~505,303.34) | 10,657.91(9,156.48~12,237.7) | -34.04(-40.25~-26.55) | -2.38(-2.72~-2.04) | 8,300.44(5,446.83~11,936.75) | 201.02(131.91~289.09) | -30.77(-50.13~-6.86) | -3.74(-4.08~-3.39) | 740,468.08(487,827.54~1065,263.04) | 17,933.05(11,814.46~25,799.1) | -31.68(-51.03~-8.13) | -3.73(-4.07~-3.39) |
| South Africa | 333,534.51(292,956.63~381,724.83) | 6,722.7(5,904.82~7,694.02) | -41.25(-45.63~-36.15) | -2.73(-3.36~-2.1) | 3,540.2(2,752.84~4,393.27) | 71.36(55.49~88.55) | -44.5(-53.89~-34.07) | -4.49(-5.2~-3.78) | 316,713.7(246,346.95~392,695.39) | 6,383.66(4,965.36~7,915.15) | -45.78(-54.95~-35.94) | -4.48(-5.19~-3.76) |
| South Sudan | 157,273.35(137,458.7~180,466.58) | 10,066.56(8,798.29~11,551.09) | -35.54(-41.78~-27.68) | -2.34(-2.47~-2.21) | 4,573.24(3,143.24~8,009.77) | 292.72(201.19~512.68) | -30.76(-46.6~-8.3) | -2.79(-3.27~-2.3) | 406,656.24(280,007.94~711,933.12) | 26,028.77(17,922.41~45,568.56) | -31.24(-46.92~-8.67) | -2.79(-3.27~-2.31) |
| Spain | 12,017.89(9,760.38~14,777.61) | 652.7(530.09~802.58) | -61.44(-66.31~-56.11) | -3.02(-3.15~-2.9) | 11.62(9.93~13.29) | 0.63(0.54~0.72) | -61.26(-66.48~-55.74) | -6.68(-8.64~-4.67) | 1,054.55(903.98~1,204.08) | 57.27(49.1~65.39) | -69.54(-74.34~-64.79) | -6.64(-8.58~-4.65) |
| Sri Lanka | 35,647.11(29,633.87~42,132.66) | 2,276.89(1,892.81~2,691.14) | -64.94(-69.52~-60.24) | -3.98(-4.27~-3.69) | 86.01(58.52~122.96) | 5.49(3.74~7.85) | -38.4(-52.58~-22.25) | -4.62(-5.71~-3.52) | 7,739.62(5,282~11,058.26) | 494.35(337.38~706.32) | -47.41(-59.51~-32.57) | -4.61(-5.69~-3.51) |
| Sudan | 146,651.36(123,363.9~174,794.36) | 2,600.63(2,187.66~3,099.7) | -68.41(-72.55~-63.58) | -4.83(-5.06~-4.6) | 2,400.39(1,656.1~3,420.95) | 42.57(29.37~60.67) | -61.33(-70.82~-48.33) | -7.76(-8.17~-7.35) | 213,953.23(147,557.04~305,135.08) | 3,794.12(2,616.69~5,411.09) | -62.88(-72.04~-50.43) | -7.75(-8.16~-7.35) |
| Suriname | 952.13(816.81~1,109.85) | 2,137.81(1,834~2,491.96) | -62.08(-65.66~-56.84) | -3.47(-3.52~-3.42) | 11.4(7.83~16.09) | 25.59(17.59~36.12) | -34.46(-51.45~-12.92) | -3.78(-4.34~-3.22) | 1,020.1(701.76~1,439.15) | 2,290.44(1,575.67~3,231.33) | -36.95(-53.85~-15.86) | -3.78(-4.34~-3.22) |
| Sweden | 4,394.19(3,444.89~5,577.93) | 753.47(590.7~956.45) | -49.94(-56.87~-40.89) | -2.35(-2.47~-2.23) | 2.77(2.25~3.41) | 0.48(0.39~0.59) | -63.34(-69.05~-55.82) | -7.13(-9.75~-4.43) | 254.31(207.45~310.82) | 43.61(35.57~53.3) | -72.39(-77.18~-66.68) | -7.06(-9.63~-4.43) |
| Switzerland | 1,838.9(1,500.77~2,259.21) | 416.05(339.54~511.14) | -65.72(-69.85~-61.16) | -3.43(-3.52~-3.33) | 2.84(2.31~3.38) | 0.64(0.52~0.76) | -72.24(-76.85~-67.55) | -6.77(-8.64~-4.86) | 256.45(208.84~304.52) | 58.02(47.25~68.9) | -76.31(-80.52~-72.23) | -6.74(-8.6~-4.85) |
| Syrian Arab Republic | 36,943.52(30,091.1~45,545.18) | 3,675.72(2,993.93~4,531.54) | -46.54(-54.2~-36.21) | -2.94(-3.04~-2.84) | 133.7(88.32~186.01) | 13.3(8.79~18.51) | -38.02(-57.9~-12.07) | -6.65(-8.37~-4.9) | 11,887.03(7,871.01~16,528.12) | 1,182.71(783.13~1,644.47) | -46.21(-63.23~-23.59) | -6.66(-8.37~-4.92) |
| Taiwan (Province of China) | 31,079.79(25,472.22~38,373.63) | 3,483.37(2,854.88~4,300.85) | -66.29(-71.35~-60.6) | -3.28(-3.59~-2.97) | 53.24(44.71~61.89) | 5.97(5.01~6.94) | -53.14(-59.8~-46.2) | -4.63(-6.18~-3.06) | 4,805.19(4,033.67~5,583.02) | 538.56(452.09~625.74) | -58.73(-64.84~-52.83) | -4.61(-6.15~-3.05) |
| Tajikistan | 59,014.39(52,793.94~66,468.6) | 4,409.05(3,944.31~4,965.96) | -55.22(-60.1~-50.27) | -3.96(-4.19~-3.72) | 2,427.38(1,674.92~3,328.94) | 181.35(125.14~248.71) | -37.03(-55.84~-20.14) | -4.1(-4.35~-3.85) | 216,727.35(149,644.96~297,196.6) | 16,192(11,180.18~22,203.97) | -38.95(-57.26~-22.49) | -4.09(-4.34~-3.84) |
| Thailand | 101,794.53(84,187.86~121,444.56) | 3,600.76(2,977.96~4,295.84) | -56.03(-61.37~-50.26) | -3.4(-3.57~-3.23) | 423.07(331.83~524.56) | 14.97(11.74~18.56) | -13.45(-36.34~16.93) | -4.82(-5.38~-4.26) | 37,892.36(29,751.56~46,931.75) | 1,340.36(1,052.4~1,660.11) | -24.12(-44.39~2.1) | -4.83(-5.38~-4.27) |
| Timor-Leste | 8,928.23(7,530.3~10,553.45) | 4,830.02(4,073.77~5,709.24) | -71.57(-75.36~-66.58) | -5.06(-5.1~-5.02) | 243.05(182.53~309.41) | 131.49(98.74~167.39) | -34.96(-48.62~-19.63) | -5.83(-6.15~-5.51) | 21,675.07(16,260.96~27,580.4) | 11,725.84(8,796.9~14,920.53) | -37.51(-51.02~-23.42) | -5.82(-6.14~-5.5) |
| Togo | 89,508.92(76,587.09~103,568.05) | 7,633.96(6,531.89~8,833.02) | -32.39(-41.11~-22.28) | -2.69(-2.94~-2.45) | 1,304.17(929.89~1,738.74) | 111.23(79.31~148.29) | -25.14(-43.66~-2.77) | -4.13(-4.4~-3.86) | 115,952.88(82,802.04~154,483.63) | 9,889.29(7,061.95~13,175.47) | -27.06(-44.77~-5.29) | -4.12(-4.39~-3.86) |
| Tokelau | 3.7(3.14~4.33) | 3,735.64(3,169.02~4,374.99) | -67.66(-70.87~-63.77) | -3.9(-4.16~-3.64) | 0.24(0.16~0.29) | 240.48(166.05~292.17) | -13.25(-37.63~14.49) | 2.42(-0.59~5.52) | 21.16(14.58~25.72) | 21,372.71(14,729.97~25,980.6) | -9.53(-34.75~19.65) | 2.41(-0.59~5.5) |
| Tonga | 497.39(428.98~572.01) | 3,452.21(2,977.36~3,970.08) | -59.5(-63.37~-55.64) | -3.14(-3.21~-3.06) | 6.41(4.54~8.46) | 44.51(31.54~58.71) | -7.68(-27.93~18.09) | -2.76(-3.18~-2.33) | 571.57(405.47~753.85) | 3,967.01(2,814.17~5,232.2) | -14.02(-33.78~10.58) | -2.76(-3.19~-2.34) |
| Trinidad and Tobago | 1,501.26(1,263.54~1,773.81) | 1,864.61(1,569.36~2,203.12) | -65.27(-69.35~-60.46) | -3.45(-3.71~-3.18) | 8.63(6.16~11.83) | 10.72(7.65~14.69) | -37.46(-45.9~-27.8) | -3.66(-4.38~-2.93) | 774.8(554.01~1,060.57) | 962.32(688.1~1,317.26) | -40.92(-49.19~-31.27) | -3.61(-4.33~-2.88) |
| Tunisia | 20,964.02(17,311.21~25,104.75) | 2,350.16(1,940.66~2,814.35) | -71.54(-74.97~-67.21) | -4.57(-4.66~-4.48) | 105.06(62.56~157.42) | 11.78(7.01~17.65) | -56.28(-72.4~-39.41) | -7.9(-8.27~-7.53) | 9,413.06(5,616.32~14,099.45) | 1,055.24(629.61~1,580.61) | -60.99(-75.38~-46.02) | -7.9(-8.27~-7.52) |
| Turkey | 105,732.82(86,581.33~127,371.97) | 1,904.45(1,559.5~2,294.22) | -79.8(-82.58~-76.35) | -5.94(-6.21~-5.67) | 447.97(313.62~597.81) | 8.07(5.65~10.77) | -75.17(-85.91~-64.77) | -10.41(-10.82~-10) | 40,096.66(28,076.15~53,478.7) | 722.22(505.71~963.26) | -77.84(-87.22~-68.57) | -10.4(-10.82~-9.99) |
| Turkmenistan | 22,711.16(20,024.11~25,613.36) | 4,208(3,710.13~4,745.73) | -64.58(-69.14~-59.65) | -4.3(-4.55~-4.05) | 780.97(625.74~956.27) | 144.7(115.94~177.18) | -40.08(-44.17~-35.77) | -4.61(-5.46~-3.75) | 69,700.18(55,858.05~85,329.16) | 12,914.28(10,349.56~15,810.07) | -42.32(-46.51~-38.19) | -4.61(-5.46~-3.75) |
| Tuvalu | 42.32(36.15~48.54) | 3,297.68(2,816.36~3,782.28) | -76.66(-78.96~-73.88) | -5.19(-5.23~-5.15) | 0.87(0.63~1.15) | 67.87(48.88~89.78) | -36.25(-51.57~-21.44) | -6.28(-6.52~-6.05) | 77.78(56~102.85) | 6,060.16(4,363.27~8,014.22) | -40.64(-55.59~-27.03) | -6.28(-6.52~-6.05) |
| Uganda | 431,597.51(364,443.38~523,338.86) | 5,897.98(4,980.29~7,151.67) | -53.21(-60.69~-45) | -3.55(-4~-3.1) | 4,847.73(3,010.76~7,116.67) | 66.25(41.14~97.25) | -35.23(-54.07~-9.66) | -5.04(-5.46~-4.62) | 431,171.05(267,996.83~632,160.28) | 5,892.15(3,662.3~8,638.76) | -36.43(-54.86~-11.41) | -5.04(-5.46~-4.62) |
| Ukraine | 29,561.14(24,188.17~35,291.69) | 1,856.62(1,519.17~2,216.53) | -65.01(-70.02~-58.79) | -3.54(-3.67~-3.42) | 121.31(95.68~146.99) | 7.62(6.01~9.23) | -47.93(-54.5~-41.09) | -4.39(-5.77~-3) | 10,892.51(8,605.46~13,194.24) | 684.12(540.48~828.68) | -53.13(-58.99~-47.17) | -4.39(-5.76~-3) |
| United Arab Emirates | 7,567.06(5,966.9~9,411.54) | 1,748.89(1,379.06~2,175.19) | -58.11(-63.78~-51.9) | -3.1(-3.19~-3) | 20.99(15.76~27) | 4.85(3.64~6.24) | -43.08(-59.82~-20.93) | -6.58(-7.49~-5.67) | 1,883.63(1,414.85~2,422.36) | 435.34(327~559.85) | -54.55(-69.43~-36.94) | -6.57(-7.47~-5.67) |
| United Kingdom | 27,847.63(23,683.9~32,678.67) | 761.99(648.05~894.18) | -40.09(-44.11~-35.69) | -1.85(-1.98~-1.71) | 53.73(44.11~61.3) | 1.47(1.21~1.68) | -51.32(-56.09~-46.86) | -5.23(-8.67~-1.65) | 4,844.39(3,977.44~5,523.83) | 132.56(108.83~151.15) | -58.13(-63.14~-53.72) | -5.2(-8.65~-1.61) |
| United Republic of Tanzania | 683,093.37(592,651.07~795,942.02) | 7,721.92(6,699.53~8,997.6) | -37.57(-46.05~-28.95) | -3.19(-3.54~-2.84) | 10,073.36(7,141.57~13,563.57) | 113.87(80.73~153.33) | -44.78(-57.92~-29.46) | -5.81(-6.16~-5.45) | 897,857.88(637,061.35~1208,296.51) | 10,149.69(7,201.56~13,658.99) | -46.2(-58.87~-31.32) | -5.8(-6.16~-5.45) |
| United States of America | 175,979.09(152,856.18~202,142.4) | 946.42(822.07~1,087.13) | -49.67(-53.64~-44.94) | -2.41(-2.6~-2.22) | 318.02(275.15~364.18) | 1.71(1.48~1.96) | -50.6(-56.35~-45.51) | -4.2(-5.74~-2.63) | 28,620.67(24,877.78~32,713.37) | 153.92(133.79~175.93) | -54.91(-60.63~-49.89) | -4.2(-5.72~-2.66) |
| United States Virgin Islands | 78.49(64.15~96.14) | 2,002.74(1,636.95~2,453.03) | -59.24(-62.89~-55.47) | -2.95(-3.11~-2.79) | 0.12(0.08~0.17) | 3.03(1.96~4.29) | -44.68(-62.86~-16.34) | -5.52(-5.94~-5.1) | 10.69(7~15.09) | 272.78(178.6~384.93) | -53.88(-69.36~-32.34) | -5.51(-5.93~-5.1) |
| Uruguay | 3,925.24(3,264.54~4,708.39) | 2,023.31(1,682.74~2,426.99) | -62.47(-66.42~-57.72) | -3.65(-4.27~-3.03) | 10.71(8.34~13.47) | 5.52(4.3~6.94) | -38.08(-46.14~-29.35) | -5.27(-5.99~-4.55) | 962.53(752.89~1,209.15) | 496.15(388.08~623.27) | -46.1(-53.71~-38.27) | -5.27(-5.98~-4.54) |
| Uzbekistan | 134,347.67(119,002.05~151,544.22) | 3,502.98(3,102.85~3,951.36) | -62.94(-67.12~-58) | -4.19(-4.46~-3.91) | 4,700.2(3,823.75~5,747.18) | 122.55(99.7~149.85) | -38.6(-43.77~-33.73) | -4.22(-4.7~-3.74) | 420,327.71(342,045.43~513,735.95) | 10,959.61(8,918.48~13,395.13) | -41.45(-46.33~-36.94) | -4.22(-4.7~-3.73) |
| Vanuatu | 2,223.32(1,943.64~2,567.37) | 5,274.58(4,611.06~6,090.8) | -41.06(-47.47~-34.25) | -2.51(-2.6~-2.43) | 28.11(20.04~37.31) | 66.69(47.55~88.51) | -20.58(-36~-0.45) | -2.62(-3.55~-1.69) | 2,513(1,795.05~3,333.75) | 5,961.82(4,258.55~7,908.95) | -23.47(-38.75~-3.07) | -2.63(-3.54~-1.7) |
| Venezuela (Bolivarian Republic of) | 59,542.04(51,002.6~69,962.04) | 2,725.12(2,334.29~3,202.02) | -63.48(-68.75~-57.07) | -3.64(-3.77~-3.51) | 563.09(403.99~755.31) | 25.77(18.49~34.57) | -36.63(-44.04~-27.76) | -2.98(-6.44~0.6) | 50,374.92(36,170.68~67,479.72) | 2,305.56(1,655.46~3,088.41) | -37.87(-45.05~-29.19) | -2.98(-6.43~0.59) |
| Viet Nam | 240,798.62(208,281.28~279,380.84) | 2,957.57(2,558.18~3,431.44) | -68.2(-72.04~-64.53) | -4.51(-4.68~-4.33) | 2,209.02(1,599.1~2,881.1) | 27.13(19.64~35.39) | -36.82(-50.59~-20.06) | -6.05(-6.34~-5.76) | 197,765.93(143,324.6~257,595.99) | 2,429.02(1,760.36~3,163.88) | -42.2(-54.66~-26.21) | -6.05(-6.34~-5.76) |
| Yemen | 153,875.39(130,345.49~182,684.7) | 3,275.82(2,774.9~3,889.14) | -63.35(-68.57~-56.66) | -4.77(-4.84~-4.69) | 2,535.03(1,788.08~3,371.49) | 53.97(38.07~71.77) | -49.39(-64.6~-28.47) | -6.29(-6.59~-6) | 226,514.81(159,809.55~301,033.94) | 4,822.23(3,402.15~6,408.65) | -52.66(-66.81~-33.51) | -6.29(-6.58~-5.99) |
| Zambia | 155,576.83(131,619.8~182,514.19) | 5,318.8(4,499.76~6,239.72) | -60(-65.33~-53.03) | -4.29(-4.83~-3.75) | 2,543.45(1,766.76~3,454.91) | 86.95(60.4~118.11) | -44.07(-58.4~-23.67) | -6.33(-6.59~-6.07) | 226,162.09(157,077.37~306,861.92) | 7,731.94(5,370.1~10,490.87) | -45.98(-60.09~-27.14) | -6.33(-6.59~-6.07) |
| Zimbabwe | 157,909.72(138,395.81~182,558.84) | 7,146.15(6,263.06~8,261.64) | -24.93(-32.47~-15.7) | -1.94(-2.38~-1.5) | 3,825.72(2,956.35~4,733.27) | 173.13(133.79~214.2) | -7.65(-24.04~11.36) | -1.35(-1.87~-0.84) | 341,626.18(264,065.32~422,550.95) | 15,460.18(11,950.19~19,122.4) | -8.47(-24.99~10.32) | -1.36(-1.87~-0.84) |
