# Supplementary material for: A 32-year trend analysis of lower respiratory infections in children under 5: insights from the global burden of disease study 2021
Source: Front Public Health. 2025 Jan 22;13:1483179. doi: 10.3389/fpubh.2025.1483179 (PMC11794078; doi:10.3389/fpubh.2025.1483179)
Supplement: Supplementary file 2 [file Supplementary_file_2.docx]

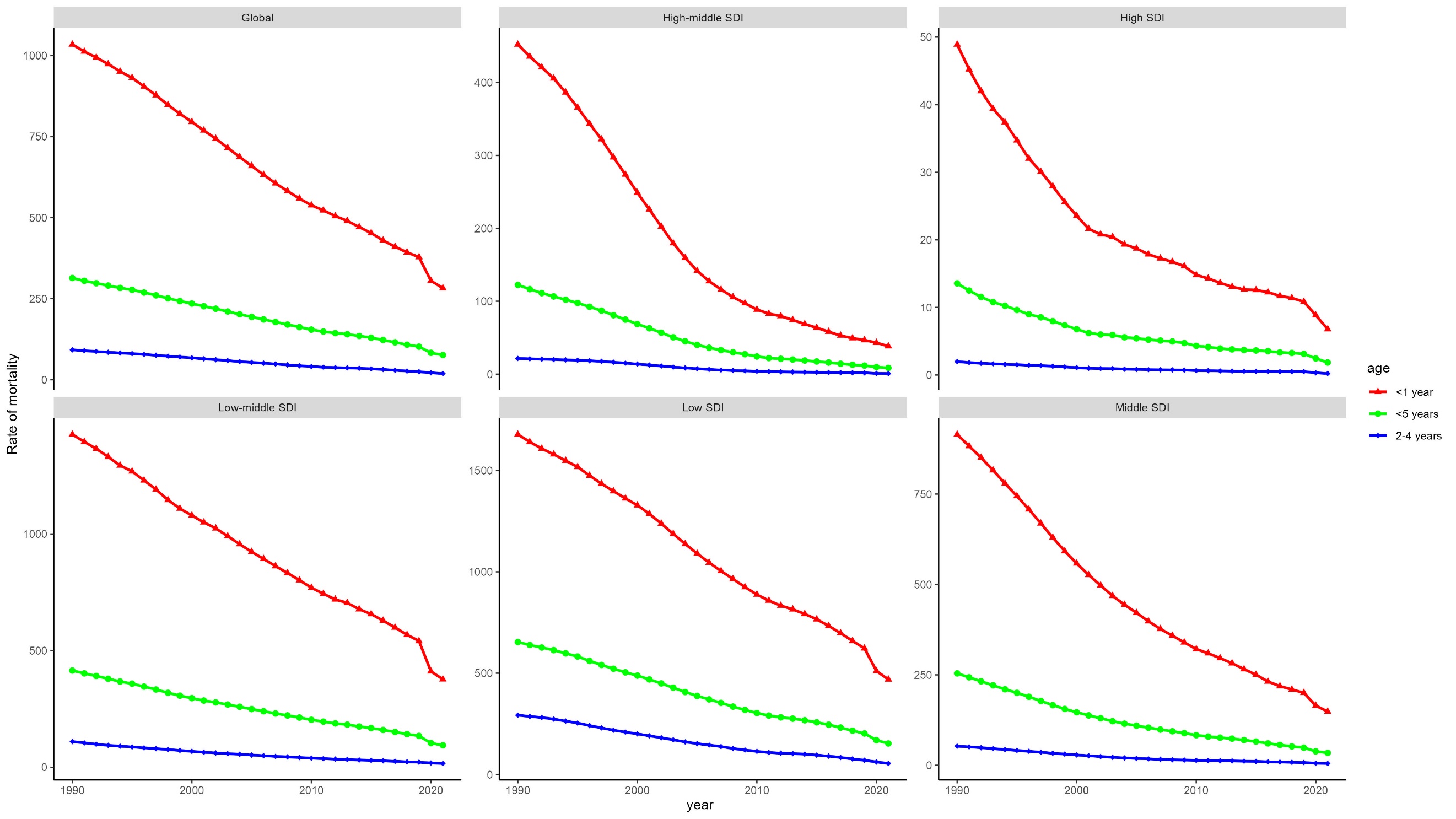


Figure S1 Global and five SDI regions mortality cases and rate in 2021


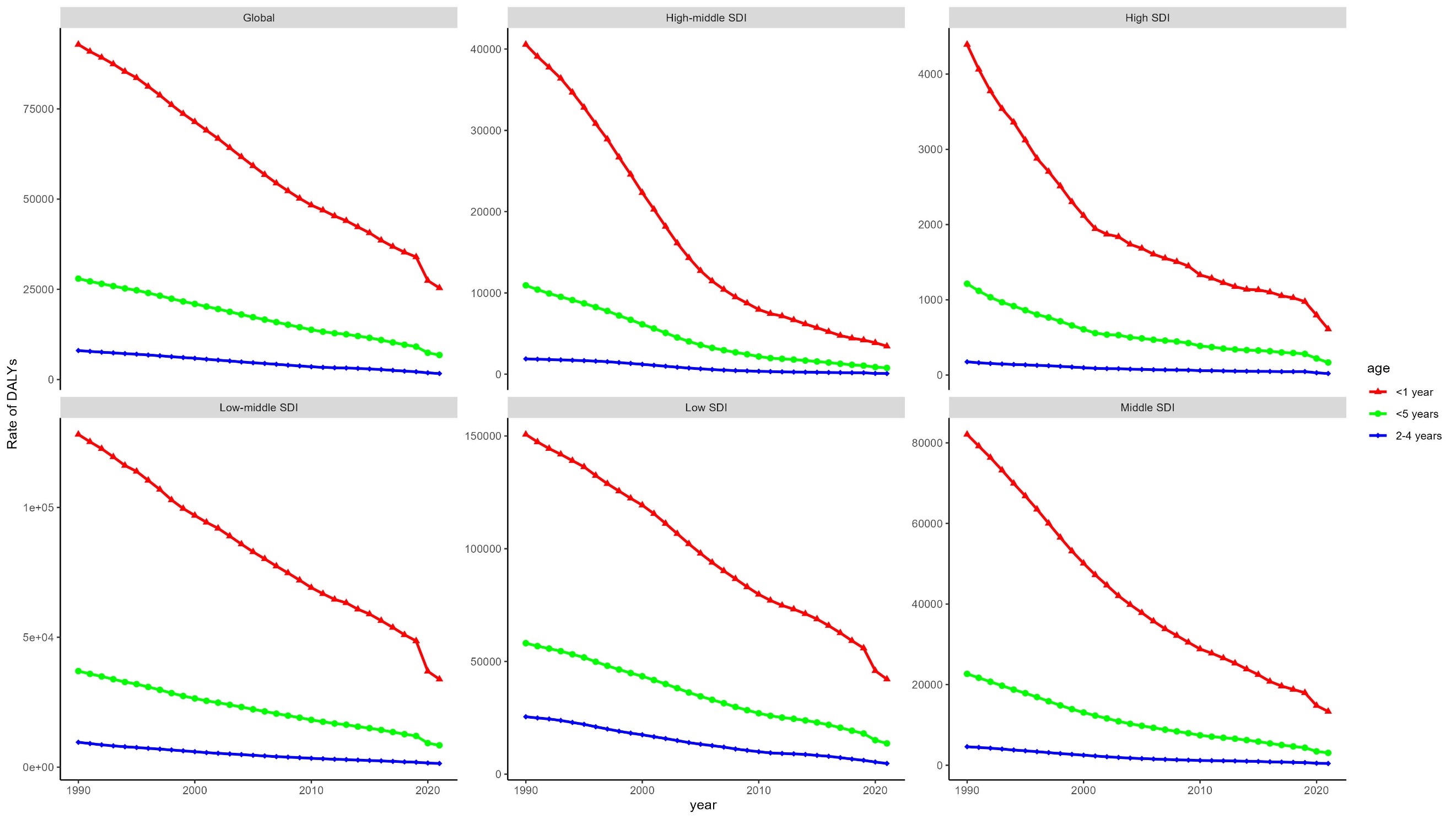


Figure S2 Global and five SDI regions DALYs cases and rate in 2021


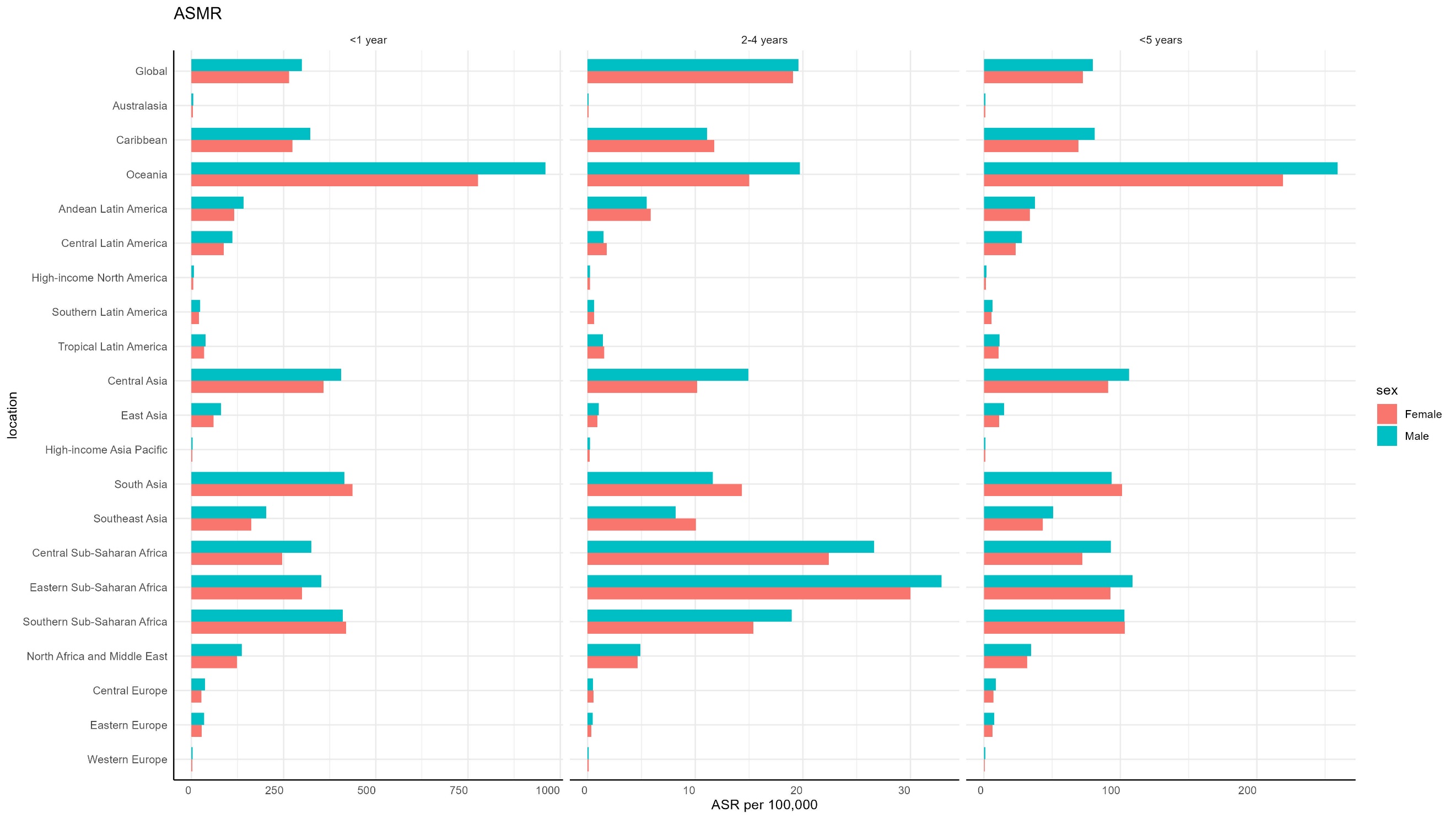
 Figure S3 ASMR caused by LRIs in children of different genders<1 year old, 2-4 years old, and<5 years old in 2021


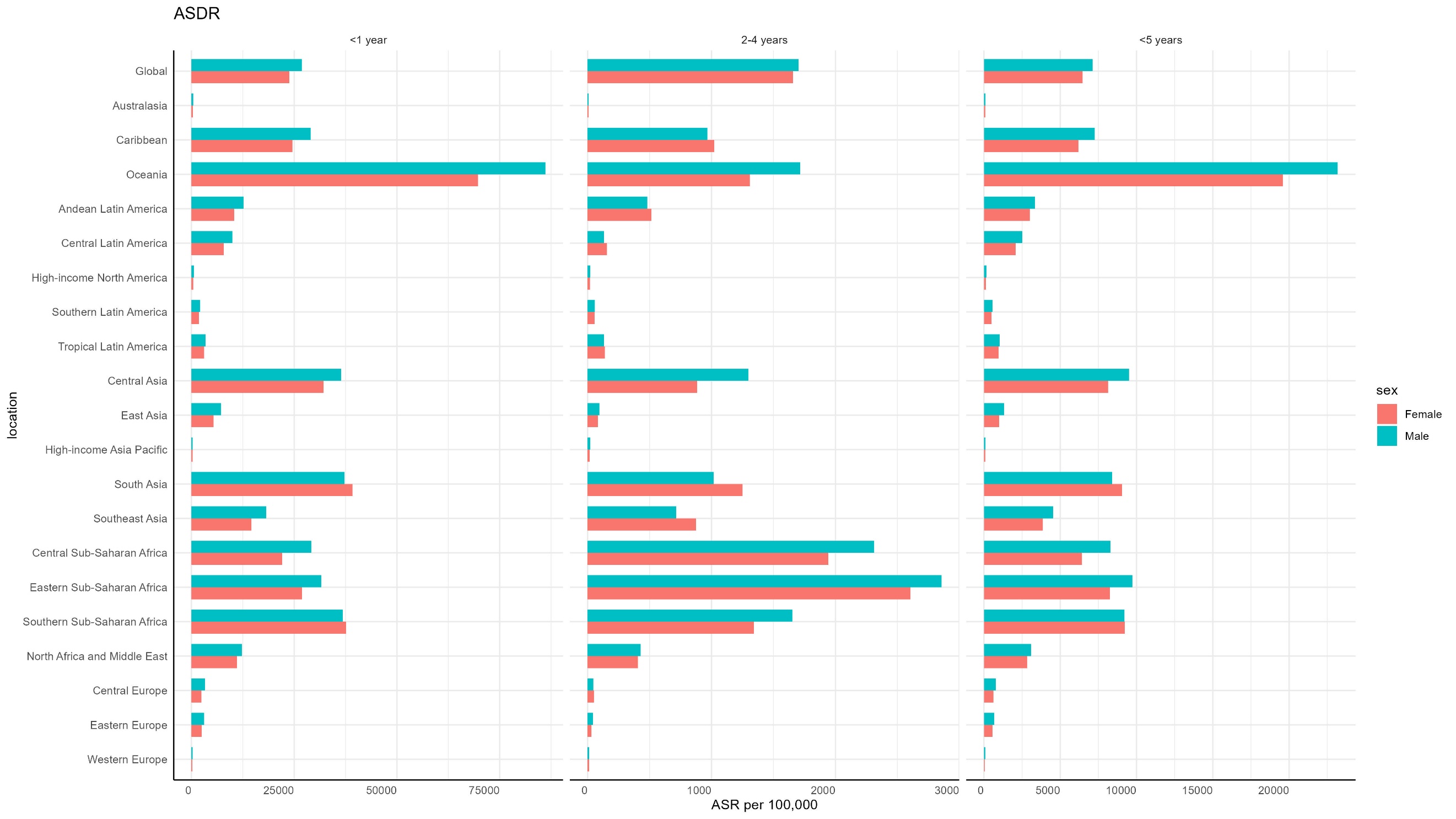


Figure S4 ASDR caused by LRIs in children of different genders <1 year old, 2-4 years old, and<5 years old in 2021


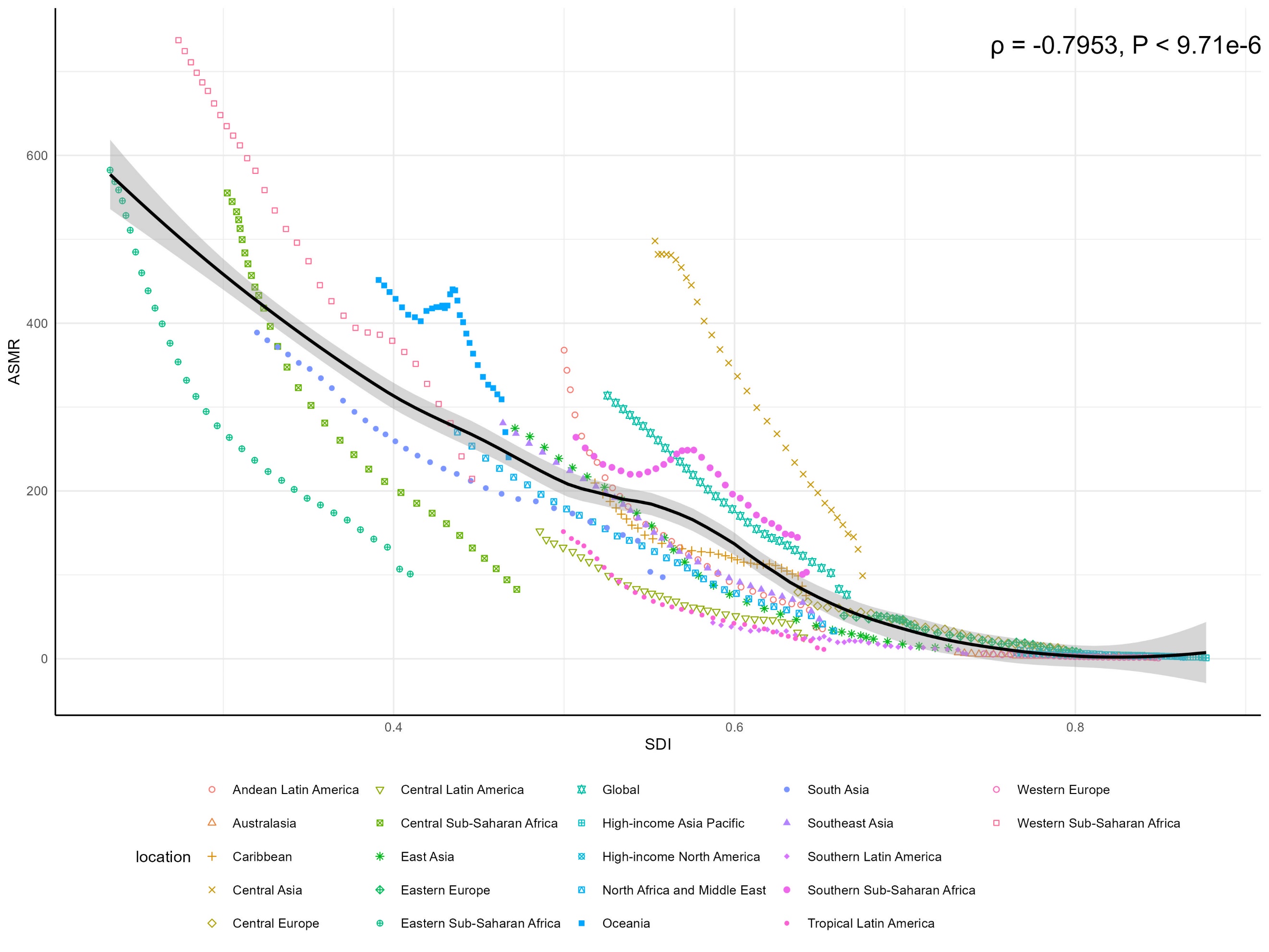


Figure S5 Correlations between ASMR of LRIs in children under 5 years old and SDI at the global and 21 regional levels from 1990 to 2021. ASMR, age-standardized mortality rate; SDI, socio-demographic index.


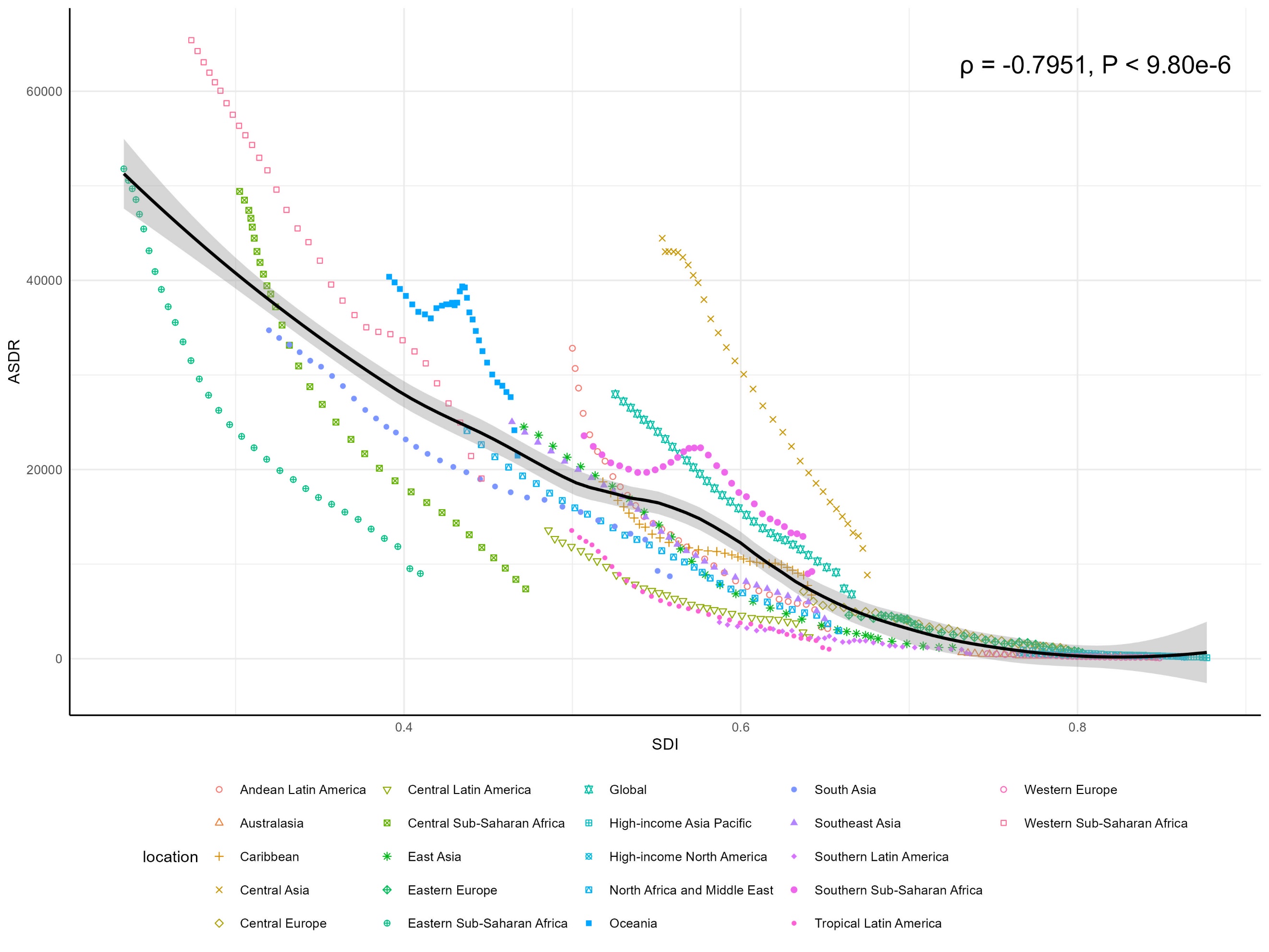


Figure S6 Correlations between ASDR of LRIs in children under 5 years old and SDI at the global and 21 regional levels from 1990 to 2021. ASDR, age-standardized DALYs rate; SDI, socio-demographic index.


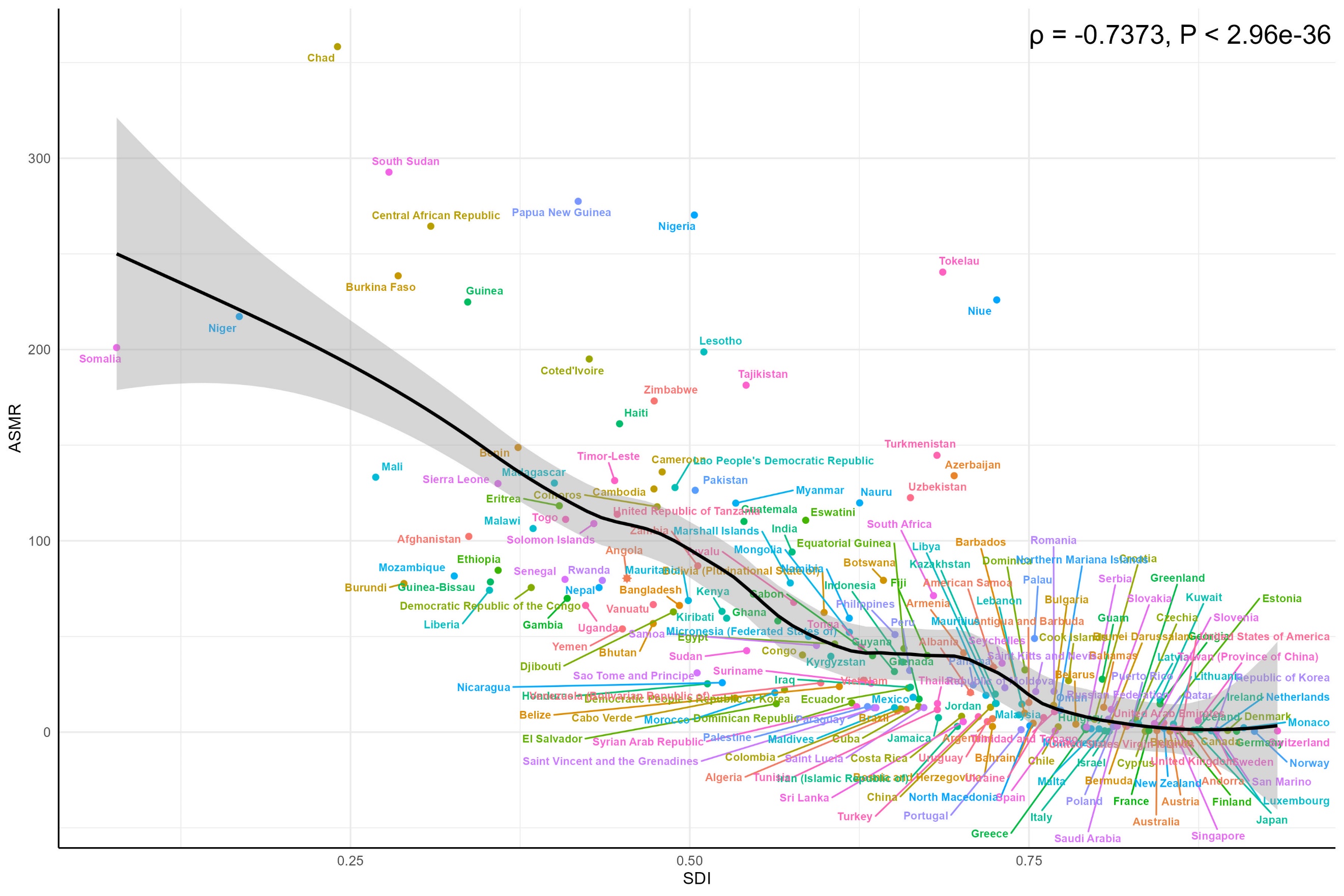


Figure S7 Correlations between ASMR of LRIs in children under 5 years old and SDI in 204 countries and territories, in 2021 ASMR, age-standardized mortality rate; SDI, socio-demographic index.


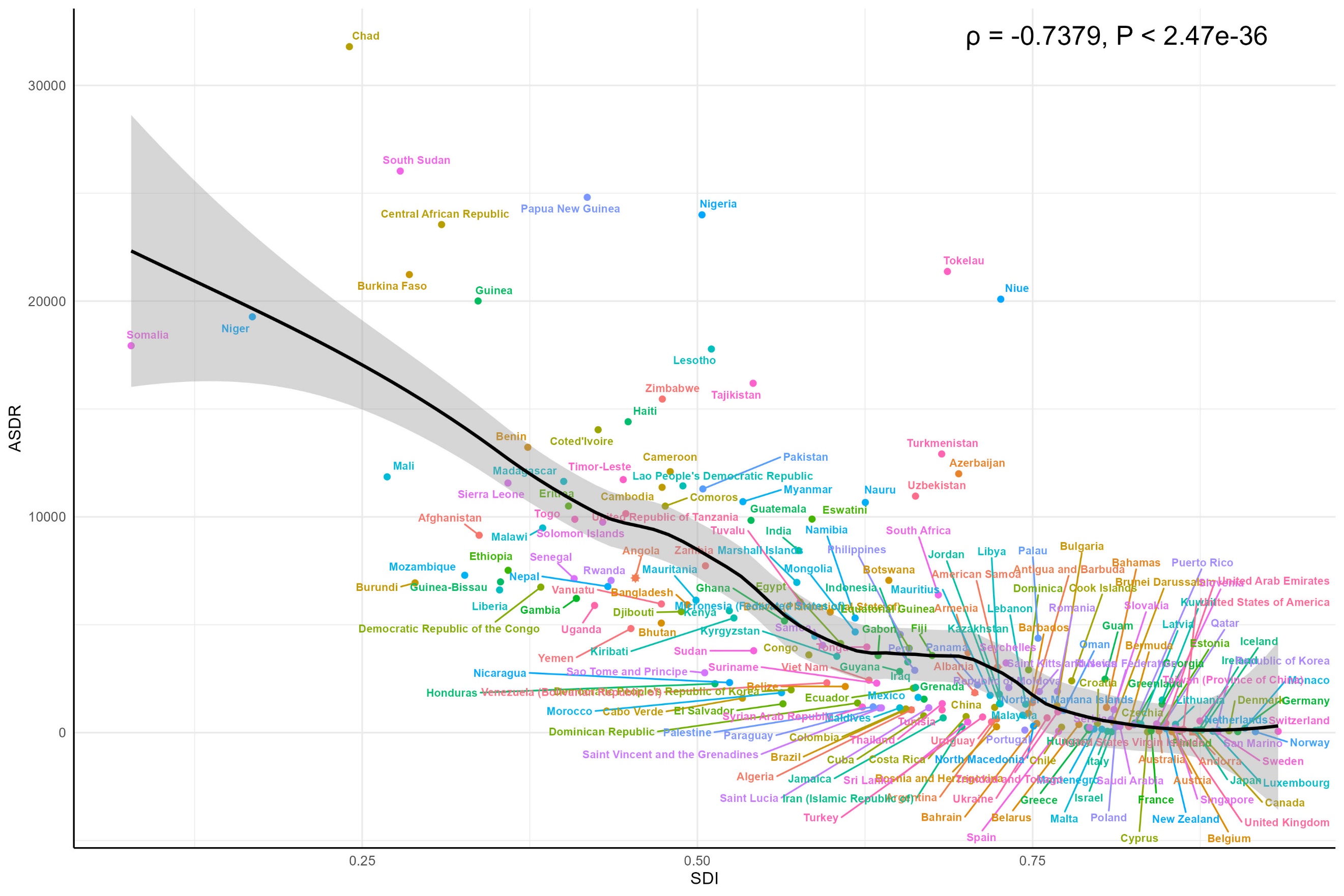


Figure S8 Correlations between ASDR of LRIs in children under 5 years old and SDI in 204 countries and territories, in 2021 ASDR, age-standardized DALYs rate; SDI, socio-demographic index.
